# Application of Chitosan/Poly(vinyl alcohol) Stabilized Copper Film Materials for the Borylation of α, β-Unsaturated Ketones, Morita-Baylis-Hillman Alcohols and Esters in Aqueous Phase †

**DOI:** 10.3390/molecules28145609

**Published:** 2023-07-24

**Authors:** Bojie Li, Wu Wen, Wei Wen, Haifeng Guo, Chengpeng Fu, Yaoyao Zhang, Lei Zhu

**Affiliations:** 1 School of Chemistry and Materials Science, Hubei Key Laboratory of Quality Control of Characteristic Fruits and Vegetables, Hubei Engineering University, Xiaogan 432000, Chinaghf02062023@163.com (H.G.);; 2 School of Materials Science and Engineering, Hubei University, Wuhan 430062, China

**Keywords:** chitosan/PVA-stabilized copper nanoparticles, copper catalysis, heterogeneous catalyst, aqueous phase, recycle and reuse

## Abstract

A chitosan/poly(vinyl alcohol)-stabilized copper nanoparticle (CP@Cu NPs) was used as a heterogeneous catalyst for the borylation of α, β-unsaturated ketones, MBH alcohols, and MBH esters in mild conditions. This catalyst not only demonstrated remarkable efficiency in synthesizing organoboron compounds but also still maintained excellent reactivity and stability even after seven recycled uses of the catalyst. This methodology provides a gentle and efficient approach to synthesize the organoboron compounds by efficiently constructing carbon–boron bonds.

## 1. Introduction

Organoboron compounds are a significant class of organic intermediates that are capable of reacting with various nucleophilic reagents and could be conveniently converted into various chemical bonds, such as C–C, C–O, and C–N bonds, which are very important in organic synthesis [1,2,3]. For this reason, in the past decades, various transition metals, including rhodium [4], palladium [5,6], platinum [7], cobalt [8], nickel [9], and copper [10,11,12], have been used as catalysts to synthesize the organoboron compounds. Recently, metal nanoparticles have received much attention because of their large surface area, which could increase catalytic efficiency [13,14]. However, metal nanoparticles sometimes have some drawbacks in the aqueous phase; for example, they are prone to aggregation and precipitation. To overcome these problems, an appropriate carrier was considered to load these metal nanoparticles, which could make these loaded nano-catalytic materials have several good advantages, including easy recovery and uniform dispersion [15,16].

Montmorillonite [17,18,19], activated carbon [20,21,22], and chitosan [23,24,25,26] are commonly used as carriers for heterogeneous catalytic materials in organic synthesis. Among these materials, chitosan is especially preferred due to its numerous amino and hydroxyl groups. These functional groups could coordinate well with various metals to perform a good catalytic activity. So far, several transition metals have been reported using chitosan as a support, including gold [27], silver [28], ruthenium [29], palladium [30,31,32], platinum [33], and copper [34,35,36]. Compared with these precious metals, copper has received more attention for its low price and lower toxicity.

**Scheme 1:**
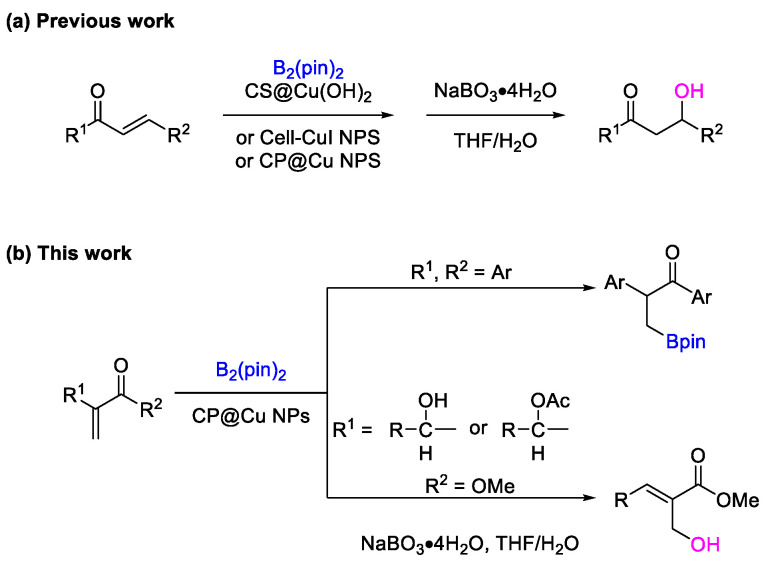
Copper-catalyzed borylation reactions of α, β-unsaturated compounds.

In our previous research, we found that the borylation reactions of chalcone derivatives could be carried out smoothly, and the corresponding target products could also be obtained in good yields when CS@Cu(OH)_2_ [37], Cell-CuI NPs [38], or CP@Cu NPs [39] were used as catalysts (Scheme 1a). Under oxidative conditions, the resulting organoborons could give rise to the desired β-hydroxy-substituted carbonyl compounds, which are widely found in active molecules [40,41,42]. Compared with other supports, chitosan has inherent advantages due to its green property, abundance, stability, and ability of chelation [26]. With our continuous efforts in exploring applications of chitosan-supported metal catalysts, we were interested in developing a chitosan composite film of stabilized copper nanoparticles and its application for the synthesis of useful organoboron compounds. In particular, chitosan/poly(vinyl alcohol) composite films loaded with copper nanoparticles (CP@Cu NPs) were found to exhibit high reactivity, as well as excellent reusability and stability. Not only did α, β-unsaturated ketones have good reactivity in borylation reactions, but also α, β-unsaturated esters and amides could react smoothly when CP@Cu NPs was used as a catalyst in the reactions, and even after the catalyst was reused seven times, it still showed very good catalytic activity. However, in our previous work, the CP@Cu NPs were limited to the borylation reactions of 1,2-disubstituted α, β-unsaturated compounds, whereas the borylation reactions of 1,1-disubstituted unsaturated compounds were not explored, including the borylation reactions of MBH alcohols, and esters were also not involved. Morita-Baylis-Hillman alcohols or esters have aroused much attention in organic synthesis as valuable synthons and intermediates for the preparation of many important cyclic and acyclic compounds. Thus, their ready availability and condensed functional groups make them particularly attractive. In recent years, considering the importance of MBH alcohols and esters in organic synthesis, more and more research groups pay attention to their applications, especially as electrophilic reagents in borylation reactions [43,44,45,46,47,48,49,50,51,52,53]. These methods still have some shortcomings; for instance, the precious metal palladium as a catalyst is needed in reactions [43,51,52,53]. Even with copper as a catalyst, the reaction substrate range is quite limited, and only MBH alcohols or esters are compatible in these methods [44,45,46,47,48,49,50]. And more importantly, because all of the above methods are homogeneous reactions, the catalysts in reactions are difficult to be separated and reused after the reactions, which resulted in waste and heavy metal residues. Herein, in this work, we used CP@Cu NPs as a heterogeneous catalyst and 1,1-disubstituted α, β-unsaturated compounds (including α, β-unsaturated ketones, MBH alcohols, and esters) as substrates. The borylation reactions of these compounds could be achieved under mild conditions. Considering that some organoboron compounds are not very stable, the corresponding β-hydroxy-substituted carbonyl compounds were obtained via direct oxidation. Finally, the activity and stability of the catalysts were proved by the recovery experiments (Scheme 1b).

## 2. Results and Discussion

### 2.1. Catalysis of CP@Cu NPs in the Borylation Reaction of α, β-Unsaturated Ketones

The initial experiments commenced with α, β-unsaturated ketone **II-1** (0.2 mmol) as a model substrate. CP@Cu NPs (10.0 mg, 9.0 mol%) was used as a catalyst by using B_2_(pin)_2_ (0.4 mmol, 2.0 equiv) as a boron source in 2.0 mL of solvents. First, various organic solvents were investigated, and no additives were added (Table 1, entries 1–7). When THF and toluene were used as solvents in this reaction, no reaction happened (Table 1, entries 1–2). Surprisingly, when ether was used as a solvent, the reaction occurred, and the target product was obtained in 8% yield (Table 1, entry 3). We continued to explore other solvents, such as MeOH, acetone, and H_2_O; the reaction could still happen, but the yields increased not obviously (Table 1, entries 4–6). In our previous work, we found that when we used mixed solvents in the reaction, excellent yields could be gained [37,38,39]. Considering the role of protons in this reaction, we used MeOH and H_2_O as mixed solvents (MeOH/H_2_O = 3:1), and the yield was increased to 28% yield (Table 1, entry 7). Next, we intended to examine the effects of additives in the reactions, mainly various organic bases, including 2,2-bipyridine, DMAP, 2-cyanopyridine, 2-chloropyridine, 2-bromopyridine, 2,6-dibromopyridine, and 4-picoline (Table 1, entries 8–14). We found that when we used 4-picoline as an additive in the reaction, the reaction worked very well, and the yield could obviously increase to 60% yield (Table 1, entry 14). Inspired by this result, we considered that the ratio of MeOH and H_2_O may have some contribution to these reactions. When we changed the ratio of the mixed solvents, different results were observed (Table 1, entries 15–18). In particular, when the ratio of MeOH to H_2_O was 1:1, the best result 92% yield could be obtained (Table 1, entry 16). In the organic synthesis, the reaction time is also one of the important factors that would affect the yield; therefore, we carried out the examination of the reaction time (Table 1, entries 19–21 vs. 16), and it was found that the reaction efficiency was still the highest when the reaction time was 12 h (Table 1, entry 16) and the yield was decreased whether the reaction time was shortened or prolonged. In order to study the effect of the amounts of additives on the reactions, we reduced or increased the amounts of additives and found that it actually had an effect on the reaction, and the yields were reduced to a certain extent (Table 1, entries 22–23 vs. 16). Finally, we investigated the catalyst loading in the reactions. When the catalyst loading was reduced to 4.5 mol%, the yield was still 92% (Table 1, entry 24), but when the amount of catalyst was increased to 13.5 mol%, the yield decreased to 90% (Table 1, entry 25). Therefore, we chose to use 4.5 mol% of catalyst loading to carry out the reactions from the view of economy. Thus, by a series of optimizations of the conditions, the optimal conditions of this research were 4.5 mol% CP@Cu NPs as a catalyst, 2.0 equiv of B_2_(pin)_2_ as a boron source, and 6.0 mol% of 4-picoline as an additive, and the whole reaction was conducted in 2.0 mL of mixed solvents (MeOH/H_2_O = 1:1) at room temperature for 12 h (Table 1, entry 24). 

**Scheme 2:**
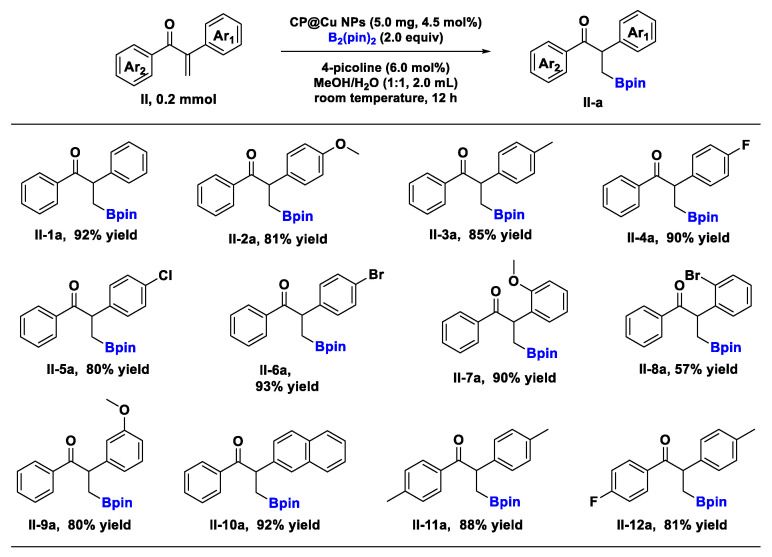
Screening substrate scope of CP@Cu NPs in the borylation reaction of α, β-unsaturated ketones. Reaction conditions: **II** (0.2 mmol), B_2_(pin)_2_ (2.0 equiv), CP@Cu NPs (5.0 mg, 4.5 mol%), 4-picoline (6.0 mol%) in 2.0 mL of mixed solvents (MeOH/H_2_O =1:1) at room temperature for 12 h.

With the optimal experimental conditions in hand, we continued to investigate the universality of the reaction, and the results are summarized in Scheme 2. We mainly examined the effects of the substituents on the benzene ring of 1,1-disubstituted α, β-unsaturated ketones on the yields (Scheme 2). We first investigated the para-substituted functional groups on the benzene ring Ar_1_; when the substituents were methoxyl and methyl, the yields of the borylation were slightly decreased, and the possible reason was that both methoxyl and methyl were electron-donating groups that had an effect on the electrophilicity of the substrates (**II-2a-II-3a**, 81–85% yields). When the substituents were changed to fluorine and bromine, the yields were very good (**II-4a**, 90% yield; **II-6a**, 93% yield). However, when chlorine was used as a substituent, the yield was decreased to 80% yield (**II-5a**), mainly because it had less electron absorption than fluorine and bromine. Next, we examined the ortho-substituents on the benzene ring Ar_1_; the electron-donating group methoxyl had a better yield than the electron-withdrawing group bromine (**II-7a**, 90% yield, vs. **II-8a**, 57% yield). We also investigated the reactivity of meto-position of the benzene ring Ar_1_, and the yields of the reactions were still good (**II-9a-II-10a**, 80–92% yields). Last, we found that the substituents on the para-position of the benzene ring Ar_2_ had little effect on the yields; neither was it the electron-donating group methyl, nor the electron-withdrawing group fluorine (**II-11a-II-12a**, 81–88% yields, vs. **II-3a**, 81% yield). 

### 2.2. Catalysis of CP@Cu NPs in the Borylation Reaction of MBH Alcohols and Esters 

MBH alcohols and esters are very important intermediates in organic synthesis, and there is not much research on the borylation reactions of these compounds at present. Therefore, in this work, we planned to use them as reaction substrates for condition optimization. The same as the above condition optimizations, we selected MBH alcohols **III-1** (0.2 mmol) or MBH esters **IV-1** (0.2 mmol) as a model substrate, CP@Cu NPs (5.0 mg, 4.5 mol%) as a catalyst, and B_2_(pin)_2_ (0.4 mmol, 2.0 equiv) as a boron source in 2.0 mL of solvents; the whole reaction was conducted at room temperature for 12 h, and no additives were needed (Table 2). According to the above experimental results we achieved, we believed that proton solvents were beneficial to this reaction, so we just chose methanol and water as the solvents for screening. First, when we used methanol as a solvent, both MBH alcohol **III-1** and **IV-1** could react smoothly, and considering that the intermediates **III-1a** and **IV-1a** were not very stable in these conditions, we directly further oxidized these intermediates to the corresponding β-hydroxy substituted products using excessive NaBO_3_•4H_2_O as an oxidant (entry1, 31% yield; entry 2, 45% yield). And when H_2_O was used as a solvent, both reaction yields were not improved (entry 3, 33% yield; entry 4, 20% yield). Then, based on our previous experiment results, the mixed solvents were beneficial to this reaction, and we considered using methanol and water as the mixed solvents for conditional screening. We investigated the ratio of methanol to water in a mixed solvent and found that the highest yield could be obtained while the ratio of methanol to water was 2:1, and the target products could be obtained in 93% (entry 7 for **III-1b**) and 92% (entry 8 for **IV-1b**) isolated yields. Finally, we investigated the catalyst loading and found that the yields of the reactions did not change much. From the economic point of view, 4.5 mol% of catalyst loading was still the best choice in the reactions. Therefore, the optimal conditions for this reaction were MBH alcohols **III-1** (0.2 mmol) or MBH esters **IV-1** (0.2 mmol) as a model substrate, CP@Cu NPs (5.0 mg, 4.5 mol%) as a catalyst, and B_2_(pin)_2_ (0.4 mmol, 2.0 equiv) as a boron source in 2.0 mL of mixed solvent (MeOH/H_2_O = 2:1), and the whole reaction was conducted at room temperature for 12 h (entry 7 for **III-1b**, entry 8 for **IV-1b**).

**Scheme 3:**
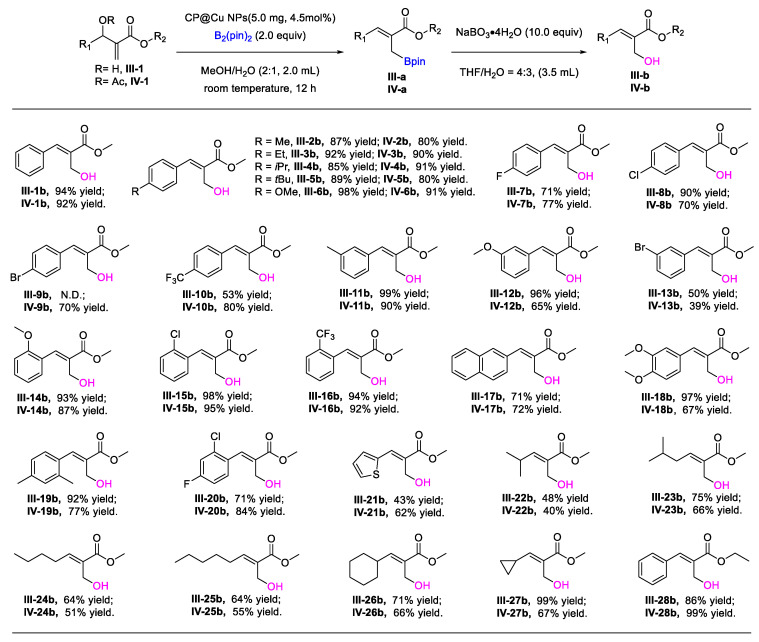
Screening substrate expansion scope of CP@Cu NPs in the borylation reaction of MBH alcohols and esters. Reaction conditions: **III-1** and **IV-1** (0.2 mmol), B_2_Pin_2_ (0.4 mmol), CP@Cu (5.0 mg), 2.0 mL of mixed solvents with MeOH/H_2_O =3:1 for **III-1** reaction and MeOH/H_2_O =2:1 for **IV-1** reaction, at room temperature for 12 h. N.D. = no detection.

With the optimized conditions in hand, we investigated the universality of the borylation reactions of MBH alcohols and esters, and the results are summarized in Scheme 3. We first examined the reactions as group R^1^ was different; when R^1^ was 4-methylphenyl, 4-ethylphenyl, 4-isopropylphenyl, 4-tert-butylphenyl, and 4-methoxyphenyl, the effect of the substituent on the reaction was not significant, whether they were MBH alcohols (**III-2b-III-6b**, 85–98% yields) or esters (**IV-2b-IV-6b**, 80–91% yields). However, when group R^1^ was 4-fluorophenyl, 4-chlorophenyl, 4-bromophenyl, and 4-trifluoromethylphenyl, the reaction results were not very good compared with the model reaction. Especially for MBH alcohols, when R^1^ was 4-bromophenyl, the target product was not detected (**III-9b**), and when R^1^ was 4-trifluoromethylphenyl, the yield was not good because of its strong electron absorption (**III-10b**, 53% yield). When R^1^ consisted of 3-substituted benzene rings, the electronic effect of the benzene ring had a great influence on the reactions. When the benzene ring was connected with electron-donating groups, such as 3-methyl and 3-methoxy, the yields were better, but for the electron-deficient group, for example, 3-bromophenyl, the yield had a great influence (**III-13b**, 50% yield; **IV-13b**, 39% yield). However, when R^1^ was 2-substituted phenyl, the electronic effect of the aromatic rings had no effect on the reactions, no matter whether they were electron-absorbing substituents or electron-giving substituents (**III-14b-III-16b**, 93–98% yields; **IV-14b-IV-16b**, 87–95% yields). For the disubstituted benzenes of R^1^, no matter whether they were electron-absorbing substituents or electron-giving substituents, the reactions could still have good yields (**III-17b-III-20b**, 71–97% yields; **IV-17b-IV-20b**, 67–80% yields). To our delight, when R^1^ was the 2-thiophene substituent, the reactions could still take place, and the target products could be obtained in medium yields (**III-21b**, 43% yield; **IV-21b**, 62% yield). Next, we continued to investigate the reactions when R_1_ consisted of alkyl groups. From the reaction results, we found that the alkyl substituents could occur smoothly, and the target products could be synthesized in medium to excellent yields (**III-22b-III-27b**, 48–99% yields; **IV-22b-IV-27b**, 40–66% yields). Finally, we investigated the reaction of R^2^ and found that when R^2^ was ethyl, the reaction activity was still very good, and the corresponding target product could be obtained with good yields (**III-28b**, 86% yield; **IV-28b**, 99% yield). 

### 2.3. Recycling Experiments of CP@Cu NPs in Borylation Reactions

**Figure 1:**
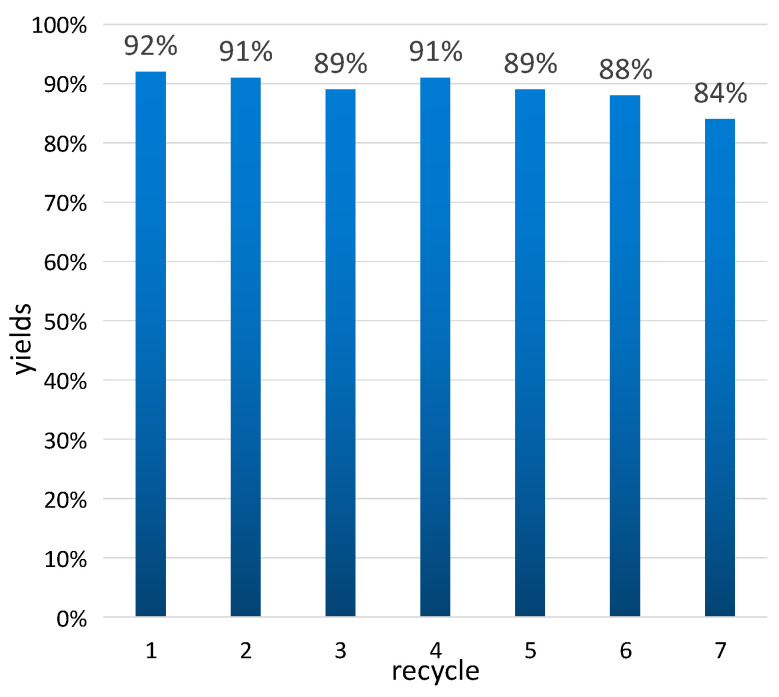
The recycle experiments.

The main advantage of heterogeneous catalysis in organic synthesis was that the catalyst in the system could be easily recovered and reused. Such a type of operation could not only increase the catalytic efficiency of the catalyst and reduce the cost of the reactions but also avoid the heavy metal residue to the environment. In this work, to assess the reusability and stability of the CP@Cu NPs in borylation reactions, we used MBH alcohols **III-1** as a substrate and CP@Cu NPs as a catalyst. After the completion of the reaction, the catalyst CP@Cu NPs could be recycled with a simple operation. The results showed that the activity of the catalyst stayed very well, and the yield of the product could also still be up to 84% even in the seventh experiment, which confirmed that the catalyst could be recyclable (Figure 1). Notably, the yields of the eighth and ninth cycles were still 83% and 82%, respectively. The slight decrease in the yields that was observed in the recycling experiments was probably due to the formation of a byproduct, which may be absorbed onto the surface of CP@Cu NPs. It must also be mentioned that the catalyst could be reactivated by washing with 10% aq. NaOH solution and dried again after the reaction. By using this process, an average of ~90% yield could be obtained after each cycle. Furthermore, ICP tests of recycled catalyst were carried out, and almost no detectable copper leaching was observed. These results strongly indicated that the CP@Cu NPs was a highly active heterogeneous catalyst for this borylation process.

## 3. Materials and Methods

### 3.1. Materials

Chitosan/poly(vinyl alcohol) composite film-supported copper nanoparticles (CP@Cu NPs) were prepared, according to the procedures reported [39]. The characterization of CP@Cu NPs was described in the supplementary materials. Bis(pinacolato)diboron (B_2_(pin)_2_, AR), methanol (MeOH, AR), ethanol (EtOH, AR), acetone (AR), tetrahydrofuran (THF, AR), ether (Et_2_O, AR), 2,2-bipyridine (AR), 4-dimethylaminopyridine (DMAP, AR), 2-cyanopyridine (AR), 2-chloropyridine (AR), 2-bromopyridine (AR), and 4-picoline (AR) were obtained commercially from Energy Chemical (Shanghai, China).

### 3.2. Synthesis of α, β-Unsaturated Ketones ***II***

In step 1, a mixture of substituted phenylacetonitrile (10 mmol), substituted phenylboronic acid (20 mmol), Pd (OAc)_2_ (112.3 mg, 0.5 mmol), 2,2′-dipyridine (156.2 mg, 1.0 mmol), TFA (11.4 g, 100 mmol), and H_2_O (4 mL) were added into THF (20 mL). Then the mixture was refluxed under nitrogen atmosphere for 2–3 days. The residue was extracted with EtOAc (20 mL) three times. After evaporation of solvent, the crude mixture was purified by flash column chromatograph to afford the intermediate compounds.

In step 2, to the compounds (5 mmol) obtained from step 1, formaldehyde (0.60 g, 20 mmol), piperidine (42.1 mg, 0.5 mmol), AcOH (60.1 mg, 1.0 mmol), and MeOH (5 mL) were added. The mixture was then refluxed for 6 h. After the completion of this reaction, evaporation was carried out to remove MeOH. The residue was washed with CH_2_Cl_2_ to collect the organic layer, which was washed with brine, dried over Na_2_SO_4_, and concentrated in vacuo. The desired ketones **II** [54,55] were obtained by further purification by silica gel chromatography.

### 3.3. Synthesis of MBH Alcohols ***III***

Different substituted benzaldehyde (10 mmol), methyl acrylate (1.72 g, 20 mmol) or ethyl acrylate (2.00 g, 20 mmol), and DABCO (1.12 g,10 mmol) were successively added into a 50 mL flask under air. After stirring for 3–7 days at room temperature, the reaction mixture was filtered, and the filtrate was extracted with EtOAc (20 mL) three times. The crude mixture was purified by silica gel chromatography to afford the desired MBH alcohols **III** [56].

### 3.4. Synthesis of MBH Esters ***IV***

Different substituted MBH alcohols **III** (10 mmol), acetic anhydride (1.23 g, 12 mmol), 4-DMAP (122.2 mg, 1 mmol), and DCM (10 mL) were successively added to a 50 mL flask under air. The reaction was monitored by TLC. After completion of reaction, the mixture was filtered, and the filtrate was extracted with EtOAc (20 mL) three times. Then the crude mixture was purified by silica gel chromatography to afford the corresponding MBH esters **IV** [56].

### 3.5. Analytical Methods

The purification of products was accomplished by using flash column chromatography on silica gel (200–300 mesh) or preparative TLC. Nuclear magnetic resonance (NMR) spectra were recorded on a Bruker Avance III 400 MHz spectrometer (Karlsruhe, Germany) operating at 400 MHz for ^1^H and 100 MHz for ^13^C NMR in CDCl_3_ unless otherwise noted. CDCl_3_ served as the internal standard (δ = 7.26 ppm) for ^1^H NMR and (δ = 77.0 ppm) for ^13^C NMR.

### 3.6. Copper-Catalyzed Borylation Reactions

The reaction procedure is depicted in Scheme 1b. Nano-sized copper loaded onto the membrane material could enable the borylation reaction of α, β-unsaturated ketones, MBH alcohols, and esters under very mild conditions. Because of the difference in reactivity, the borylation of α, β-unsaturated ketones with B_2_(pin)_2_ required the additional bases, whereas the borylation of MBH alcohols and esters could be conducted smoothly without the bases.

#### 3.6.1. Borylation Reactions of α, β-Unsaturated Ketones

At room temperature, 0.2 mmol of α, β-unsaturated ketones **II**, 0.4 mmol of B_2_(pin)_2_, 6 mol% of base, 10.0 mg of CP@Cu NPs as a catalyst, and 2.0 mL of solvent were added in the reaction system. The whole reactions were stirred at room temperature for 12 h, and after completion of the reaction, the mixture was filtered, and the desired products **II-a** were obtained by being purified with column chromatography and characterized by ^1^H NMR and ^13^C NMR (see supplementary materials).

#### 3.6.2. Borylation Reactions of MBH Alcohols and Esters

At room temperature, a reaction mixture containing 0.2 mmol of MBH alcohols or esters (**III** or **IV**), 0.4 mmol of B_2_(pin)_2_, 10.0 mg of CP@Cu NPs, and 2.0 mL of solvent were prepared, and the whole reactions were stirred at room temperature. After the completion of the reactions, the organic phase was separated, and the crude intermediates **III-a** or **IV-a** were added to a mixture of THF-H_2_O containing an excess of sodium perborate, and the mixture continued to be stirred for 4 h. When the reaction finished, the desired products **III-b** or **IV-b** were obtained, purified by column chromatography, and characterized by ^1^H NMR and ^13^C NMR (see supplementary materials).

### 3.7. General Procedure for ICP Test of CP@Cu NPs

Chitosan/poly(vinyl alcohol) composite film supported copper nanoparticles (CP@Cu NPs) (~20 mg) were placed in a clean test tube and heated with H_2_SO_4_ (1 mL) at 200 °C. After 30 min, several drops of concentrated HNO_3_ were added carefully, and the tube was shaken occasionally. HNO_3_ was continuously added until a clear solution was obtained, and the excess amount of HNO_3_ was allowed to evaporate under heating. After the solution was cooled to room temperature, 1 mL of aqua regia was added carefully. The effervescence of gas was observed, and the solution became clearer. The solution was then transferred to a volumetric flask and increased up to 50 mL with water, which was submitted for ICP analysis.

### 3.8. General Procedure for the Sample Preparation for ICP Analysis to Determine Metal Leaching

After the reaction was finished, the reaction mixture was filtered. The filtrate obtained was concentrated and diluted with 10 mL of THF. Then, 50% *v*/*v* of the crude THF solution (5 mL) was then passed through a membrane filter (0.25 or 0.45 µm) into a clean test tube. After the evaporation of the solvent, the solid obtained in the test tube was heated to 200 °C, and 1.0 mL of concentrated H_2_SO_4_ was added. Following a procedure similar to that described above, concentrated HNO_3_ was added at regular intervals until the resulting solution was clear. After the solution was cooled to room temperature, 1 mL of aqua regia was added carefully. The effervescence of the gas was observed, and the solution became clearer. The solution was then transferred to a volumetric flask and increased up to 50 mL with water, which was submitted for ICP analysis.

## 4. Conclusions

In summary, we reported the preparation of a chitosan-loaded copper catalyst (CP@Cu NPs) and its application in the borylation of α, β-unsaturated ketones, MBH alcohols, and esters with B_2_(pin)_2_ as a boron source. The whole reaction conditions were very mild, and no additives were even needed in the borylation of the MBH alcohols and esters. It demonstrated that the substrate scope of this newly developed method was very broad (more than 40 examples) with very high activity of the catalyst (up to 99% yield). Remarkably, a single heterogeneous catalyst could efficiently catalyze three types of substrates including the borylation of α, β-unsaturated ketones, MBH alcohols, and esters. Moreover, this newly developed strategy could largely solve the recovery of copper catalysts, providing a green and economic way for the efficient synthesis of organoboron compounds in the aqueous phase.

## Figures and Tables

**Table 1:** Optimization of CP@Cu NPs in the borylation reaction of α, β-unsaturated ketones ^a^.

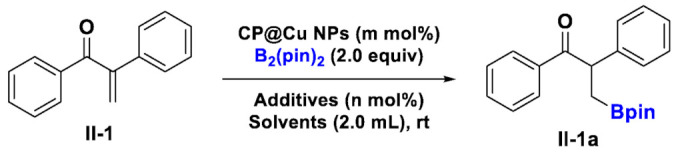					
**Entries**	**CP@Cu NPs**	**Solvents (2.0 mL)**	**Additives**	**Time (h)**	**NMR Yields (%)**
1	(9.0 mol%)	THF	-	12	N.R.
2	(9.0 mol%)	Toluene	-	12	N.R.
3	(9.0 mol%)	Et_2_O	-	12	8
4	(9.0 mol%)	MeOH	-	12	16
5	(9.0 mol%)	Acetone	-	12	8
6	(9.0 mol%)	H_2_O	-	12	19
7	(9.0 mol%)	MeOH/H_2_O = 3:1	-	12	28
8	(9.0 mol%)	MeOH/H_2_O = 3:1	2,2-bipyridine	12	35
9	(9.0 mol%)	MeOH/H_2_O = 3:1	DMAP	12	33
10	(9.0 mol%)	MeOH/H_2_O = 3:1	2-cyanopyridine	12	54
11	(9.0 mol%)	MeOH/H_2_O = 3:1	2-chloropyridine	12	57
12	(9.0 mol%)	MeOH/H_2_O = 3:1	2-bromopyridine	12	50
13	(9.0 mol%)	MeOH/H_2_O = 3:1	2,6-dibromopyridine	12	48
14	(9.0 mol%)	MeOH/H_2_O = 3:1	4-picoline	12	60
15	(9.0 mol%)	MeOH/H_2_O = 2:1	4-picoline	12	72
16 ^b^	(9.0 mol%)	MeOH/H_2_O = 1:1	4-picoline	12	92
17	(9.0 mol%)	MeOH/H_2_O = 1:2	4-picoline	12	89
18	(9.0 mol%)	MeOH/H_2_O = 1:4	4-picoline	12	80
19	(9.0 mol%)	MeOH/H_2_O = 1:1	4-picoline	4	79
20	(9.0 mol%)	MeOH/H_2_O = 1:1	4-picoline	8	87
21 ^b^	(9.0 mol%)	MeOH/H_2_O = 1:1	4-picoline	16	90
22 ^b,c^	(9.0 mol%)	MeOH/H_2_O = 1:1	4-picoline	12	90
23 ^b,d^	(9.0 mol%)	MeOH/H_2_O = 1:1	4-picoline	12	90
24 ^b^	(4.5 mol%)	MeOH/H_2_O = 1:1	4-picoline	12	92
25 ^b^	(13.5 mol%)	MeOH/H_2_O = 1:1	4-picoline	12	90

Reaction conditions: ^a^ **II-1** (0.2 mmol), B_2_Pin_2_ (0.4 mmol), CP@Cu NPS (10.0 mg, 9.0 mol%), additives (6.0 mol%, 0.012 mmol), solvents (2.0 mL) at room temperature. ^b^ Isolated yield. ^c^ 4-picoline (5.0 mol%). ^d^ 4-picoline (7.0 mol%). N.R. = no reaction.

**Table 2:** Optimization of CP@Cu NPs in the borylation reaction of MBH alcohols and esters ^a^.

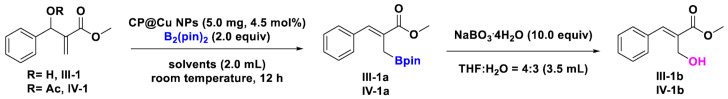				
**Entries**	**Substrates**	**CP@Cu NPs**	**Solvents (2.0 mL)**	**NMR Yield** **s (%)**
1	**III-1**	5.0 mg	MeOH	31
2	**IV-1**	5.0 mg	MeOH	45
3	**III-1**	5.0 mg	H_2_O	33
4	**IV-1**	5.0 mg	H_2_O	20
5	**III-1**	5.0 mg	MeOH/H_2_O =3:1	94
6	**IV-1**	5.0 mg	MeOH/H_2_O =3:1	90
7 ^b^	**III-1**	5.0 mg	MeOH/H_2_O =2:1	93
8 ^b^	**IV-1**	5.0 mg	MeOH/H_2_O =2:1	92
9	**III-1**	5.0 mg	MeOH/H_2_O =1:1	90
10	**IV-1**	5.0 mg	MeOH/H_2_O =1:1	86
11	**III-1**	5.0 mg	MeOH/H_2_O =1:2	89
12	**IV-1**	5.0 mg	MeOH/H_2_O =1:2	83
13	**III-1**	5.0 mg	MeOH/H_2_O =1:3	88
14	**IV-1**	5.0 mg	MeOH/H_2_O =1:3	85
15	**III-1**	10.0 mg	MeOH/H_2_O =2:1	93
16	**IV-1**	10.0 mg	MeOH/H_2_O =2:1	91

Reaction conditions: ^a^ **III-1** and **IV-1** (0.2 mmol), B_2_(pin)_2_ (0.4 mmol), CP@Cu (5.0 mg, 4.5 mol%), solvents (2.0 mL) at room temperature for 12 h. ^b^ Isolated yields.

## Supplementary Materials

The following supporting information can be downloaded at: https://www.mdpi.com/article/10.3390/molecules28145609/s1.
