# Supplementary material for: Application of Chitosan/Poly(vinyl alcohol) Stabilized Copper Film Materials for the Borylation of α, β-Unsaturated Ketones, Morita-Baylis-Hillman Alcohols and Esters in Aqueous Phase
Source: Molecules. 2023 Jul 24;28(14):5609. doi: 10.3390/molecules28145609 (PMC10386186; doi:10.3390/molecules28145609)
Supplement: Supplementary file 1 [file molecules-28-05609-s001.zip › molecules-2501886-supplementary.pdf]

Supplementary information

Application of Chitosan/Poly(vinyl alcohol) Stabilized Copper Film Materials for the Borylation of α, β-Unsaturated Ketones, MBH Alcohols and Esters in Aqueous Phase†

Bojie Li [1], Wu Wen [1,2], Wei Wen [1,2], Haifeng Guo [1,2], Chengpeng Fu [1,2], Yaoyao Zhang [1,*], Lei Zhu [1,2,*]

[1] School of Chemistry and Materials Science, Hubei Key Laboratory of Quality Control of Characteristic Fruits and Vegetables, Hubei Engineering University, Xiaogan 432000, China.

[2] School of Materials Science and Engineering, Hubei University, Wuhan 430062, China.

* Correspondence: Lei.zhu@hbeu.edu.cn (L.Z.), yaoyaozhang@hbeu.edu.cn (Y. Z.)

† Dedicated to the 80th anniversary celebration of Hubei Engineering University.

Table of Contents

1. General Procedure for the Preparation of CP@Cu NPs 1

2. IR spectra of CP@Cu NPs 2

3. SEM image of CP@Cu NPs...3

4. NMR data of products (**II-1a- II-12a** and **III-1b- III-28b**)...................4

5. ^{1}H NMR and ^{13}C NMR spectra of products 177

1. General Procedure for the Preparation of CP@Cu NPs

200 mg of chitosan powder was dissolved in 10 mL of acetic acid solution (2%, v/v) and stirred at room temperature for 5 h. At the same time, 400 mg of poly (vinyl alcohol) was dissolved in 10 mL of water and stirred at 80 °C for 12 h. The two solutions obtained were mixed and stirred at room temperature for another 0.5 h; then, 32 μL of glutaraldehyde solution (25%, w/w) was added, and stirring was continued for 5 min. In order to form the chitosan/poly (vinyl alcohol) composite film, the mixed solution described above was transferred to a Petri dish and dried at 40 °C for 12 h. After completion of this procedure, 0.1 mol/L of NaOH solution was added to the above composite film and allowed to soak for 5 min; then, this was washed until it was neutral by using water and dried over 12 h at 40 °C. After immersing the composite film in 0.2 mol/L $CuCl_2$ solution for 2.5 h, the excess Cu^{2+} and Cl^{-} were removed by washing with water, and then drying took place at 40 °C for 12 h. Finally, the chitosan/poly (vinyl alcohol)-composite-film-supported copper nanoparticles (CP@Cu NPs) were obtained by reducing with 0.05 mol/L of $NaBH_4$ solution, and they were then submitted for ICP analysis. The copper loading of the CP@Cu NPs was found to be 1.78 mmol/g.

2. IR spectra of CP@Cu NPs

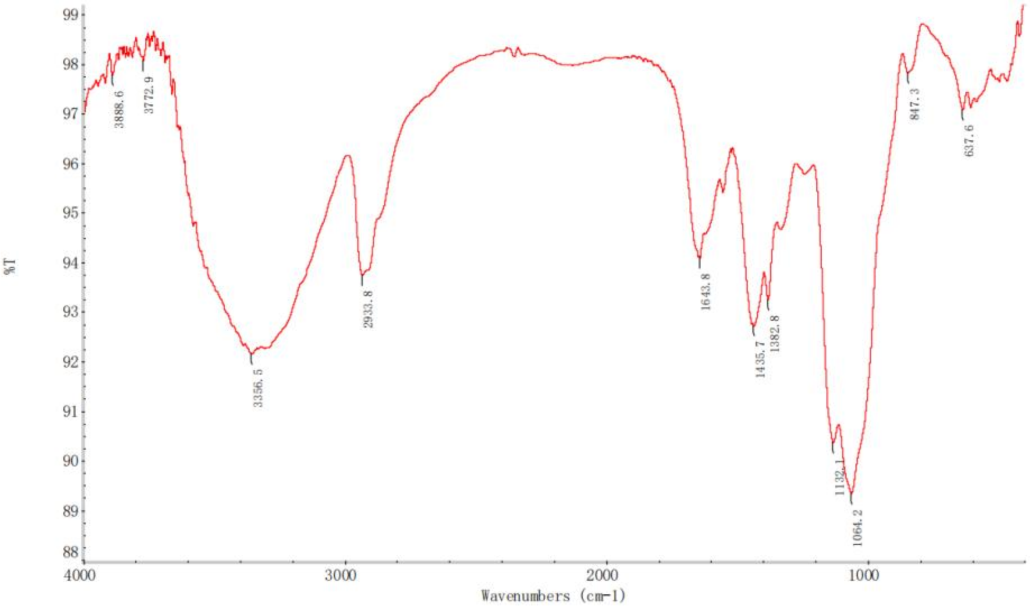

Figure S1 IR spectra of CP@Cu NPs

3. SEM image of CP@Cu NPs

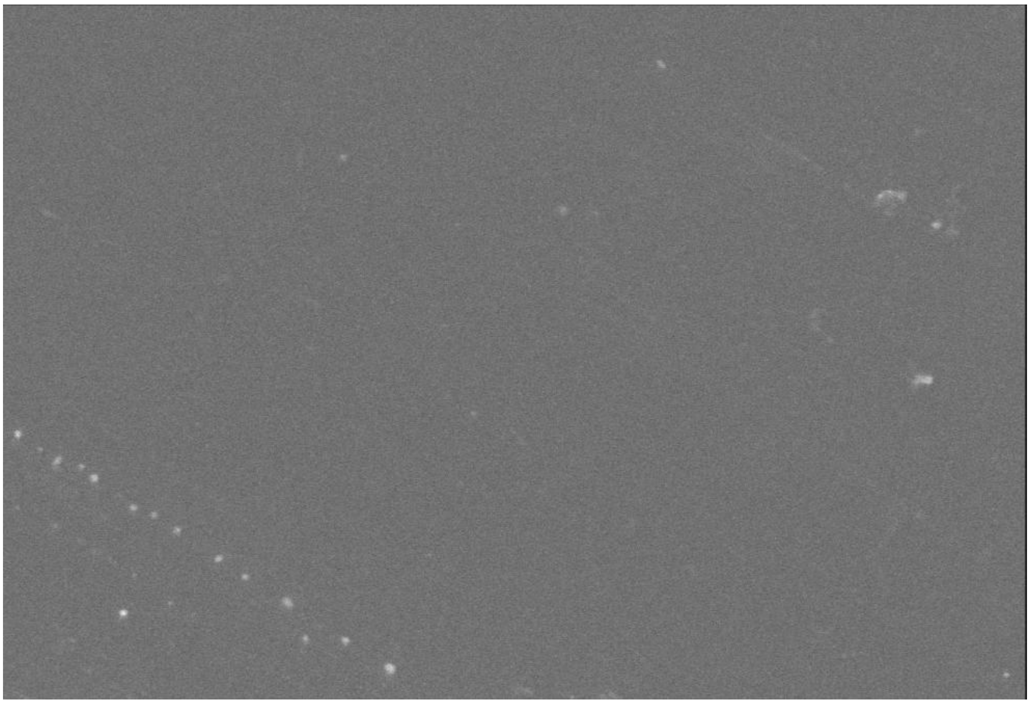

Figure S2 SEM image of CP@Cu NPs

4. NMR data of products (**II-1a- II-12a** and **III-1b- III-28b**)

II-1a: 1,2-diphenyl-3-(4,4,5,5-tetramethyl-1,3,2-dioxaborolan-2-yl)propan-1-one

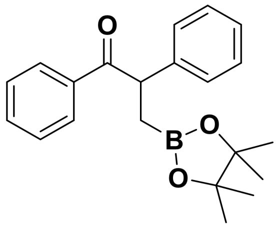

White solid

^{1}H NMR (400 MHz, Chloroform-d) δ 8.00 – 7.85 (m, 2H), 7.48 – 7.40 (m, 1H), 7.34 (t, *J* = 7.5 Hz, 2H), 7.30 – 7.21 (m, 4H), 7.18 – 7.11 (m, 1H), 4.85 – 4.69 (m, 1H), 1.58 (dd, *J* = 15.9, 9.2 Hz, 1H), 1.34 (dd, *J* = 15.9, 6.7 Hz, 1H), 1.19 (s, 6H), 1.12 (s, 6H).

^{13}C NMR (100 MHz, Chloroform-d) δ 200.74, 142.00, 136.65, 132.48, 128.89, 128.86, 128.30, 128.00, 126.69, 83.23, 50.17, 24.80, 24.59.

II-2a:

2-(4-methoxyphenyl)-1-phenyl-3-(4,4,5,5-tetramethyl-1,3,2-dioxaborolan-2-yl)propan-1-one

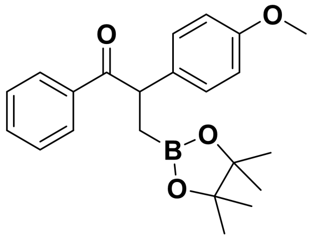

White solid

^{1}H NMR (400 MHz, Chloroform-d) δ 7.97 – 7.88 (m, 2H), 7.48 – 7.40 (m, 1H), 7.38 – 7.31 (m, 2H), 7.23 – 7.12 (m, 2H), 6.83 – 6.71 (m, 2H), 4.79 – 4.70 (m, 1H), 3.73 (s, 3H), 1.55 (dd, *J* = 15.9, 9.1 Hz, 1H), 1.32 (dd, *J* = 15.9, 6.9 Hz, 1H), 1.19 (s, 6H), 1.13 (s, 6H).

^{13}C NMR (100 MHz, Chloroform-d) δ 200.93, 158.28, 136.66, 134.01, 132.43, 129.03, 128.88, 128.30, 114.23, 83.21, 55.19, 49.22, 24.80, 24.62.

II-3a:

1-phenyl-3-(4,4,5,5-tetramethyl-1,3,2-dioxaborolan-2-yl)-2-(*p*-tolyl)propan-1-one

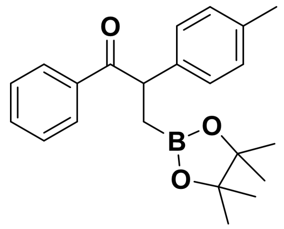

White solid

^{1}H NMR (400 MHz, Chloroform-d) δ 7.97 – 7.87 (m, 2H), 7.47 – 7.39 (m, 1H), 7.38 – 7.29 (m, 2H), 7.15 (d, *J* = 8.1 Hz, 2H), 7.05 (d, *J* = 7.9 Hz, 2H), 4.81 – 4.71 (m, 1H), 2.25 (s, 3H), 1.57 (dd, *J* = 15.9, 9.5 Hz, 1H), 1.30 (dd, *J* = 15.9, 6.5 Hz, 1H), 1.19 (s, 6H), 1.13 (s, 6H).

^{13}C NMR (100 MHz, Chloroform-d) δ 200.88, 139.03, 136.68, 136.25, 132.42, 129.59, 128.91, 128.28, 127.81, 83.21, 49.81, 24.81, 24.60, 21.05.

II-4a:

2-(4-fluorophenyl)-1-phenyl-3-(4,4,5,5-tetramethyl-1,3,2-dioxaborolan-2-yl)propan-1-one

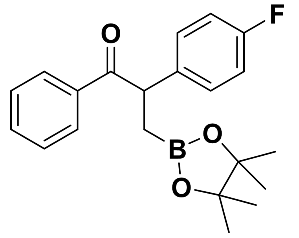

White solid

^{1}H NMR (400 MHz, Chloroform-d) δ 7.96 – 7.88 (m, 2H), 7.50 – 7.43 (m, 1H), 7.41 – 7.33 (m, 2H), 7.28 – 7.20 (m, 2H), 6.99 – 6.89 (m, 2H), 4.84 – 4.75 (m, 1H), 1.55 (dd, *J* = 16.0, 8.8 Hz, 1H), 1.34 (dd, *J* = 15.9, 7.2 Hz, 1H), 1.18 (s, 6H), 1.13 (s, 6H).

^{13}C NMR (100 MHz, Chloroform-d) δ 200.64, 162.86, 160.43, 137.59, 137.56, 136.42, 132.66, 129.60, 129.52, 128.84, 128.40, 115.80, 115.58, 83.31, 49.12, 24.76, 24.61.

II-5a:

2-(4-chlorophenyl)-1-phenyl-3-(4,4,5,5-tetramethyl-1,3,2-dioxaborolan-2-yl)propan-1-one

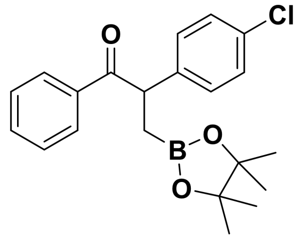

White solid

^{1}H NMR (400 MHz, Chloroform-d) δ 7.90 (d, J = 7.1 Hz, 2H), 7.50 – 7.43 (m, 1H),

7.40 – 7.32 (m, 2H), 7.22 (d, J = 1.3 Hz, 4H), 4.83 – 4.74 (m, 1H), 1.55 (dd, J = 16.0, 8.9 Hz, 1H), 1.32 (dd, J = 16.0, 6.9 Hz, 1H), 1.18 (s, 6H), 1.13 (s, 6H).

^{13}C NMR (100 MHz, Chloroform-d) δ 200.38, 140.43, 136.35, 132.73, 132.55, 129.39, 129.01, 128.84, 128.42, 83.35, 49.33, 24.77, 24.62.

II-6a:

2-(4-bromophenyl)-1-phenyl-3-(4,4,5,5-tetramethyl-1,3,2-dioxaborolan-2-yl)propan-1-one

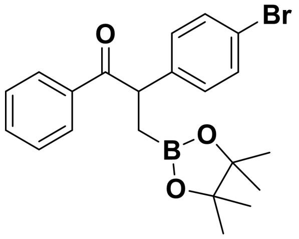

White solid

^{1}H NMR (400 MHz, Chloroform-d) δ 7.95 – 7.84 (m, 2H), 7.46 (d, J = 7.3 Hz, 1H), 7.42 – 7.33 (m, 4H), 7.21 – 7.10 (m, 2H), 4.82 – 4.68 (m, 1H), 1.55 (dd, J = 16.0, 9.0 Hz, 1H), 1.31 (dd, J = 16.0, 6.9 Hz, 1H), 1.18 (s, 6H), 1.13 (s, 6H).

^{13}C NMR (100 MHz, Chloroform-d) δ 200.29, 140.97, 136.33, 132.75, 131.96, 129.76, 128.85, 128.43, 120.67, 83.35, 49.41, 24.77, 24.63.

II-7a:

2-(2-methoxyphenyl)-1-phenyl-3-(4,4,5,5-tetramethyl-1,3,2-dioxaborolan-2-yl)propan-1-one

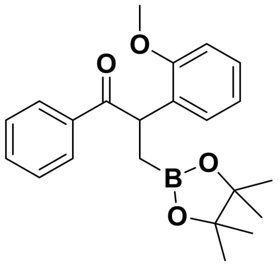

White solid

^{1}H NMR (400 MHz, Chloroform-d) δ 7.93 – 7.87 (m, 2H), 7.44 – 7.38 (m, 1H), 7.30 (t, J = 7.5 Hz, 2H), 7.17 – 7.07 (m, 2H), 6.86 – 6.75 (m, 2H), 5.16 – 5.04 (m, 1H), 3.82 (s, 3H), 1.48 (dd, J = 15.7, 10.0 Hz, 1H), 1.28 – 1.24 (m, 1H), 1.22 (s, 6H), 1.15 (s, 6H).

^{13}C NMR (100 MHz, Chloroform-d) δ 201.64, 155.70, 136.66, 132.23, 130.99, 128.67, 128.63, 128.10, 127.84, 121.01, 110.72, 83.06, 55.29, 43.66, 24.87, 24.55.

II-8a:

2-(2-bromophenyl)-1-phenyl-3-(4,4,5,5-tetramethyl-1,3,2-dioxaborolan-2-yl)propan-1-one

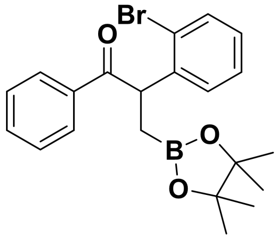

White solid

^{1}H NMR (400 MHz, Chloroform-d) δ 7.96 – 7.83 (m, 2H), 7.61 – 7.53 (m, 1H), 7.48 – 7.42 (m, 1H), 7.39 – 7.32 (m, 2H), 7.17 – 7.11 (m, 1H), 7.08 – 6.98 (m, 2H), 5.25 – 5.06 (m, 1H), 1.40 (dd, *J* = 15.8, 10.2 Hz, 1H), 1.29 (dd, *J* = 15.8, 5.6 Hz, 1H), 1.22 (s, 6H), 1.16 (s, 6H).

^{13}C NMR (100 MHz, Chloroform-d) δ 200.53, 141.83, 136.15, 133.20, 132.73, 129.00, 128.85, 128.42, 128.30, 128.12, 123.81, 83.25, 49.64, 24.85, 24.55.

II-9a:

2-(3-methoxyphenyl)-1-phenyl-3-(4,4,5,5-tetramethyl-1,3,2-dioxaborolan-2-yl)propan-1-one

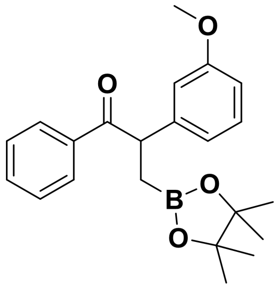

Colorless oil

^{1}H NMR (400 MHz, Chloroform-d) δ 7.97 – 7.89 (m, 2H), 7.48 – 7.41 (m, 1H), 7.35 (t, *J* = 7.5 Hz, 2H), 7.17 (t, *J* = 7.9 Hz, 1H), 6.86 (d, *J* = 7.7 Hz, 1H), 6.80 (t, *J* = 2.1 Hz, 1H), 6.73 – 6.63 (m, 1H), 4.80 – 4.71 (m, 1H), 3.74 (s, 3H), 1.58 (dd, *J* = 15.9, 9.5 Hz, 1H), 1.32 (dd, *J* = 15.9, 6.4 Hz, 1H), 1.20 (s, 6H), 1.14 (s, 6H).

^{13}C NMR (100 MHz, Chloroform-d) δ 200.60, 159.84, 143.62, 136.59, 132.53, 129.88, 128.90, 128.32, 120.39, 113.37, 112.19, 83.25, 55.16, 50.30, 24.83, 24.59.

II-10a:

2-(naphthalen-2-yl)-1-phenyl-3-(4,4,5,5-tetramethyl-1,3,2-dioxaborolan-2-yl)propan-1-one

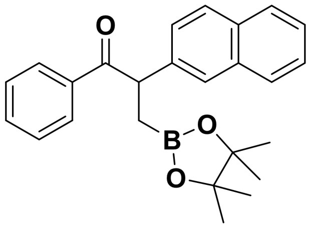

White solid

^{1}H NMR (400 MHz, Chloroform-d) δ 7.99 – 7.92 (m, 2H), 7.78 – 7.68 (m, 4H), 7.44 – 7.36 (m, 4H), 7.34 – 7.26 (m, 2H), 5.04 – 4.86 (m, 1H), 1.68 (dd, *J* = 16.0, 9.2 Hz, 1H), 1.42 (dd, *J* = 16.0, 6.7 Hz, 1H), 1.19 (s, 6H), 1.12 (s, 6H).

^{13}C NMR (100 MHz, Chloroform-d) δ 200.69, 139.65, 136.62, 133.67, 132.56, 132.28, 128.96, 128.73, 128.36, 127.75, 127.64, 126.70, 126.16, 126.09, 125.69, 83.30, 50.35, 24.81, 24.67.

II-11a: 3-(4,4,5,5-tetramethyl-1,3,2-dioxaborolan-2-yl)-1,2-di-*p*-tolylpropan-1-one

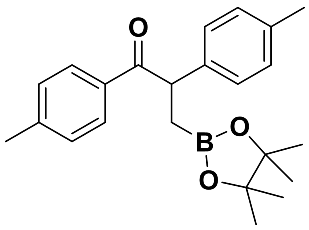

White solid

^{1}H NMR (400 MHz, Chloroform-d) δ 7.83 (d, *J* = 8.3 Hz, 2H), 7.18 – 7.10 (m, 4H), 7.05 (d, *J* = 7.8 Hz, 2H), 4.78 – 4.68 (m, 1H), 2.32 (s, 3H), 2.25 (s, 3H), 1.55 (dd, *J* = 15.9, 9.4 Hz, 1H), 1.29 (dd, *J* = 15.9, 6.5 Hz, 1H), 1.19 (s, 6H), 1.13 (s, 6H).

^{13}C NMR (100 MHz, Chloroform-d) δ 200.39, 143.11, 139.32, 136.14, 134.04, 129.56, 129.06, 129.00, 127.77, 83.17, 49.68, 24.82, 24.59, 21.62, 21.07.

II-12a:

1-(4-fluorophenyl)-3-(4,4,5,5-tetramethyl-1,3,2-dioxaborolan-2-yl)-2-(*p*-tolyl)propan-1-one

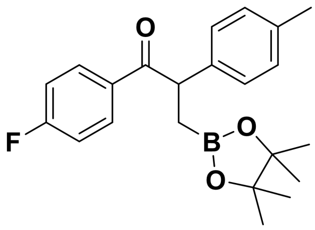

White solid

^{1}H NMR (400 MHz, Chloroform-d) δ 7.83 (d, *J* = 8.3 Hz, 2H), 7.29 – 7.20 (m, 2H), 7.16 (d, *J* = 8.0 Hz, 2H), 6.93 (t, *J* = 8.7 Hz, 2H), 4.82 – 4.70 (m, 1H), 2.34 (s, 3H), 1.53 (dd, *J* = 15.9, 8.7 Hz, 1H), 1.33 (dd, *J* = 15.9, 7.2 Hz, 1H), 1.18 (s, 6H), 1.12 (s,

6H).

^{13}C NMR (100 MHz, Chloroform-d) δ 200.15, 162.81, 160.38, 143.43, 137.85, 137.82, 133.79, 129.56, 129.48, 129.10, 129.01, 115.75, 115.54, 83.27, 48.97, 24.77, 24.61, 21.63.

III-1b: Methyl (*E*)-2-(hydroxymethyl)-3-phenylacrylate

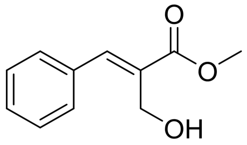

Colorless oil

^{1}H NMR (400 MHz, Chloroform-d); δ = 7.84 (s, 1H), 7.47 – 7.34 (m, 5H), 4.49 (s, 2H), 3.86 (s, 3H).

^{13}C NMR (100 MHz, Chloroform-d); δ = 168.41, 142.63, 134.46, 130.83, 129.55, 129.23, 128.57, 57.90, 52.19.

III-2b: Methyl (*E*)-2-(hydroxymethyl)-3-(*p*-tolyl)acrylate

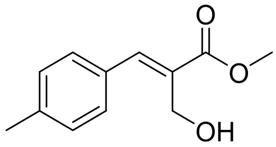

Colorless oil

^{1}H NMR (400 MHz, Chloroform-d); δ = 7.81 (s, 1H), 7.38 (d, *J* = 8.1 Hz, 2H), 7.23 (d, *J* = 7.9 Hz, 2H), 4.50 (s, 2H), 3.85 (s, 3H), 2.38 (s, 3H).

^{13}C NMR (100 MHz, Chloroform-d); δ = 168.54, 142.76, 139.59, 131.61, 129.95, 129.67, 129.32, 57.98, 52.13, 21.38.

III-3b: Methyl (*E*)-3-(4-ethylphenyl)-2-(hydroxymethyl)acrylate

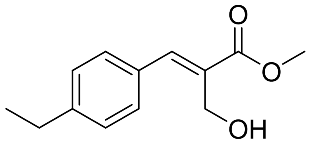

White solid

^{1}H NMR (400 MHz, Chloroform-d); δ = 7.82 (s, 1H), 7.40 (d, *J* = 7.9 Hz, 2H), 7.26 – 7.23 (m, 2H), 4.51 (s, 2H), 3.86 (s, 3H), 2.71 (q, *J* = 7.6 Hz, 2H), 1.27 (t, *J* = 7.6 Hz, 3H).

^{13}C NMR (100 MHz, Chloroform-d); δ = 168.57, 145.88, 142.78, 131.86, 129.99, 129.77, 128.15, 58.02, 52.14, 28.73, 15.36.

III-4b: Methyl (*E*)-2-(hydroxymethyl)-3-(4-isopropylphenyl)acrylate

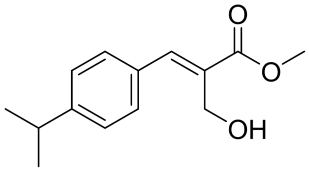

Colorless oil

^{1}H NMR (400 MHz, Chloroform-d); δ = 7.81 (s, 1H), 7.41 (d, *J* = 8.0 Hz, 2H), 7.28 – 7.26 (m, 2H), 4.51 (s, 2H), 3.86 (s, 3H), 2.97 (dq, *J* = 13.8, 6.9 Hz, 1H), 1.27 (d, *J* = 6.9 Hz, 6H).

^{13}C NMR (100 MHz, Chloroform-d); δ = 168.58, 150.46, 142.75, 132.00, 130.01, 129.79, 126.72, 58.02, 52.14, 34.01, 23.80.

III-5b: Methyl (*E*)-3-(4-(*tert*-butyl)phenyl)-2-(hydroxymethyl)acrylate

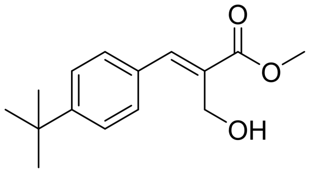

Colorless oil

^{1}H NMR (400 MHz, Chloroform-d); δ = 7.82 (s, 1H), 7.45 – 7.40 (m, 4H), 4.51 (s, 2H), 3.86 (s, 3H), 1.33 (s, 9H).

^{13}C NMR (100 MHz, Chloroform-d); δ = 168.57, 152.69, 142.66, 131.62, 130.09, 129.53, 125.56, 57.98, 52.13, 34.78, 31.16.

III-6b: Methyl (*E*)-2-(hydroxymethyl)-3-(4-methoxyphenyl)acrylate

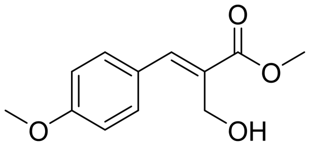

Colorless oil

1H NMR (400 MHz, Chloroform-d); δ = 7.97 (s, 1H), 7.43 – 7.33 (m, 2H), 6.99 (t, *J* = 7.6 Hz, 1H), 6.91 (dd, *J* = 8.3, 1.0 Hz, 1H), 4.43 (s, 2H), 3.86 (s, 6H).

^{13}C NMR (100 MHz, Chloroform-d); δ = 168.67, 160.58, 142.65, 131.56, 128.68, 127.01, 114.09, 58.02, 55.34, 52.10.

III-7b: Methyl (*E*)-3-(4-fluorophenyl)-2-(hydroxymethyl)acrylate

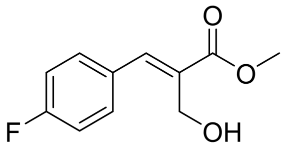

Colorless oil

^{1}H NMR (400 MHz, Chloroform-d); δ = 7.79 (s, 1H), 7.49 – 7.45 (m, 2H), 7.13 (t, *J* = 8.6 Hz, 2H), 4.46 (s, 2H), 3.86 (s, 3H).

^{13}C NMR (100 MHz, Chloroform-d); 168.32, 164.48, 161.99, 141.66, 131.72, 131.63, 130.62, 130.57, 115.90, 115.69, 57.83, 52.30.

III-8b: Methyl (*E*)-3-(4-chlorophenyl)-2-(hydroxymethyl)acrylate

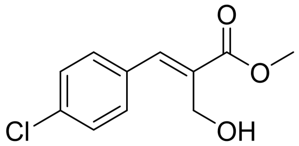

Colorless oil

^{1}H NMR (400 MHz, Chloroform-d); δ = 7.77 (s, 1H), 7.42 – 7.37 (m, 4H), 4.44 (s, 2H), 3.86 (s, 3H).

^{13}C NMR (100 MHz, Chloroform-d); δ = 168.15, 141.38, 135.38, 132.87, 131.29, 130.90, 128.87, 57.72, 52.28.

III-9b: Methyl (*E*)-3-(4-bromophenyl)-2-(hydroxymethyl)acrylate

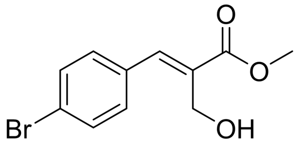

Colorless oil

^{1}H NMR (400 MHz, Chloroform-d); δ = 7.74 (s, 1H), 7.55 – 7.52 (m, 2H), 7.35 – 7.32 (m, 2H), 4.43 (s, 2H), 3.85 (s, 3H).

^{13}C NMR (100 MHz, Chloroform-d); δ = 168.13, 141.41, 133.30, 131.81, 131.37, 131.11, 123.69, 57.68, 52.28.

III-10b: Methyl (*E*)-2-(hydroxymethyl)-3-(4-(trifluoromethyl)phenyl)acrylate

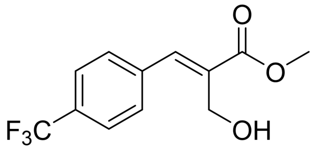

Colorless oil

^{1}H NMR (400 MHz, Chloroform-d); δ = 7.82 (s, 1H), 7.67 (d, *J* = 8.2 Hz, 2H), 7.58 – 7.55 (m, 2H), 4.43 (s, 2H), 3.87 (s, 3H).

^{13}C NMR (100 MHz, Chloroform-d); δ = 1 167.91, 140.84, 137.96, 137.95, 132.79, 131.05, 130.73, 129.71, 125.18, 57.56, 52.36.

III-11b: Methyl (*E*)-2-(hydroxymethyl)-3-(*m*-tolyl)acrylate

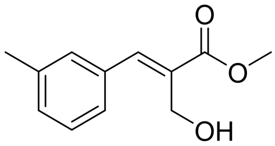

Colorless oil

^{1}H NMR (400 MHz, Chloroform-d); δ = 7.81 (s, 1H), 7.34 (t, *J* = 7.9 Hz, 1H), 7.05 –

7.00 (m, 2H), 6.94 – 6.91 (m, 1H), 4.49 (s, 2H), 3.86 (s, 3H), 3.83 (s, 3H).

^{13}C NMR (100 MHz, Chloroform-d); δ = 168.37, 159.60, 142.56, 135.77, 131.08, 129.62, 121.94, 115.10, 114.66, 57.98, 55.29, 52.22.

III-12b: Methyl (*E*)-2-(hydroxymethyl)-3-(3-methoxyphenyl)acrylate

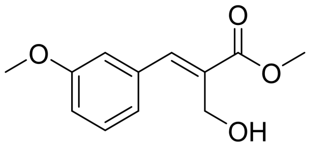

Colorless oil

^{1}H NMR (400 MHz, Chloroform-d); δ = 7.81 (s, 1H), 7.32 – 7.18 (m, 4H), 4.49 (s, 2H), 3.86 (s, 3H), 2.38 (s, 3H).

^{13}C NMR (100 MHz, Chloroform-d); δ = 168.47, 142.78, 138.26, 134.41, 130.63, 130.15, 130.04, 128.48, 126.64, 57.98, 52.17, 21.39.

III-13b: Methyl (*E*)-3-(3-bromophenyl)-2-(hydroxymethyl)acrylate

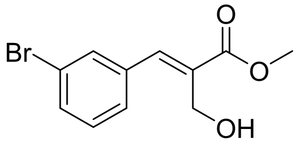

Colorless oil

^{1}H NMR (400 MHz, Chloroform-d); δ = 7.74 (s, 1H), 7.59 (d, J = 1.7 Hz, 1H), 7.52 – 7.50 (m, 1H), 7.41 – 7.38 (m, 1H), 7.31 – 7.27 (m, 1H), 4.45 (s, 2H), 3.87 (s, 3H).

^{13}C NMR (100 MHz, Chloroform-d); δ = 168.00, 140.84, 136.48, 132.24, 132.14, 132.10, 130.12, 128.02, 122.66, 57.72, 52.34.

III-14b: Methyl (*E*)-2-(hydroxymethyl)-3-(2-methoxyphenyl)acrylate

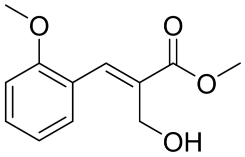

Colorless oil

^{1}H NMR (400 MHz, Chloroform-d); δ = 7.97 (s, 1H), 7.42 – 7.34 (m, 2H), 7.01 – 6.90 (m, 2H), 4.43 (s, 2H), 3.86 (s, 6H).

^{13}C NMR (100 MHz, Chloroform-d); δ = 168.46, 157.58, 138.54, 130.84, 130.63, 130.59, 123.54, 120.51, 110.50, 58.46, 55.50, 52.09.

III-15b: Methyl (*E*)-3-(2-chlorophenyl)-2-(hydroxymethyl)acrylate

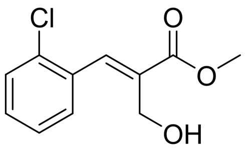

Colorless oil

^{1}H NMR (400 MHz, Chloroform-d); δ = 7.79 (s, 1H), 7.48 – 7.45 (m, 2H), 7.12 – 7.08 (m, 2H), 4.46 (s, 2H), 3.86 (s, 3H).

^{13}C NMR (100 MHz, Chloroform-d); δ = 168.28, 164.46, 161.97, 141.61, 131.68, 131.60, 130.63, 115.86, 115.65, 57.79, 52.24.

III-16b: Methyl (*E*)-2-(hydroxymethyl)-3-(2-(trifluoromethyl)phenyl)acrylate

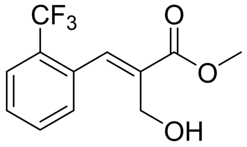

Colorless oil

^{1}H NMR (400 MHz, Chloroform-d); δ = 8.00 (d, *J* = 2.5 Hz, 1H), 7.73 (d, *J* = 7.8 Hz, 1H), 7.59 (t, *J* = 7.2 Hz, 1H), 7.49 (dd, *J* = 11.8, 7.5 Hz, 2H), 4.29 (s, 2H), 3.89 (s, 3H).

^{13}C NMR (100 MHz, Chloroform-d); δ = 167.73, 138.66, 133.29, 133.06, 131.88, 130.78, 128.81, 128.56, 126.10, 126.04, 125.99, 125.94, 58.14, 52.40.

III-17b: Methyl (*E*)-2-(hydroxymethyl)-3-(naphthalen-2-yl)acrylate

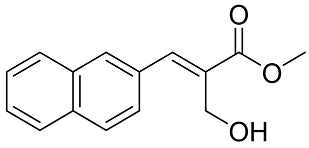

Colorless oil

^{1}H NMR (400 MHz, Chloroform-d); δ = 7.99 (s, 1H), 7.95 (s, 1H), 7.89 – 7.83 (m, 3H), 7.57 – 7.51 (m, 3H), 4.58 (s, 2H), 3.89 (s, 3H).

^{13}C NMR (100 MHz, Chloroform-d); δ = 168.45, 142.73, 133.35, 133.01, 131.93, 130.89, 129.60, 128.52, 128.26, 127.65, 127.06, 126.66, 126.56, 58.05, 52.25.

III-18b: Methyl (*E*)-3-(3,4-dimethoxyphenyl)-2-(hydroxymethyl)acrylate

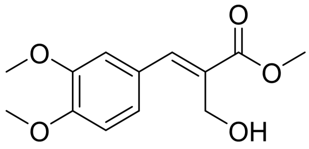

Colorless oil

^{1}H NMR (400 MHz, Chloroform-d); δ = 7.80 (s, 1H), 7.11 – 7.09 (m, 2H), 6.92 (d, *J* = 8.8 Hz, 1H), 4.52 (s, 2H), 3.92 (d, *J* = 4.3 Hz, 6H), 3.86 (s, 3H).

^{13}C NMR (100 MHz, Chloroform-d); δ = 168.61, 150.15, 148.81, 143.02, 128.86, 127.27, 123.36, 112.61, 110.90, 58.07, 55.92, 55.89, 52.15.

III-19b: Methyl (*E*)-3-(2,4-dimethylphenyl)-2-(hydroxymethyl)acrylate

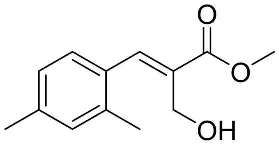

Colorless oil

^{1}H NMR (400 MHz, Chloroform-d); δ = 7.88 (s, 1H), 7.26 – 7.21 (m, 1H), 7.04 (d, *J* = 7.5 Hz, 2H), 4.40 (s, 2H), 3.87 (s, 3H), 2.34 (s, 3H), 2.28 (s, 3H).

^{13}C NMR (100 MHz, Chloroform-d); δ = 168.45, 141.53, 139.30, 137.04, 131.00, 130.79, 130.67, 129.27, 126.59, 58.33, 52.15, 21.26, 19.91.

III-20b: Methyl (*E*)-3-(2-chloro-4-fluorophenyl)-2-(hydroxymethyl)acrylate

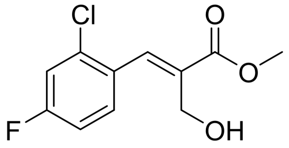

Colorless oil

^{1}H NMR (400 MHz, Chloroform-d); δ = 7.85 (s, 1H), 7.55 (dd, *J* = 8.6, 6.0 Hz, 1H), 7.20 (dd, *J* = 8.4, 2.6 Hz, 1H), 7.06 (td, *J* = 8.3, 2.6 Hz, 1H), 4.35 (s, 2H), 3.87 (s, 3H).

^{13}C NMR (100 MHz, Chloroform-d); δ = 167.86, 164.11, 161.59, 138.29, 135.35, 132.41, 132.24, 132.15, 129.30, 129.27, 117.31, 117.06, 114.43, 114.22, 58.01, 52.39.

III-21b: Methyl (*E*)-2-(hydroxymethyl)-3-(thiophen-2-yl)acrylate

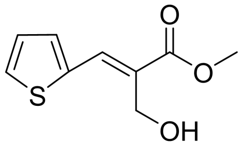

Colorless oil

^{1}H NMR (400 MHz, Chloroform-d); δ = 7.88 (s, 1H), 7.54 (d, *J* = 5.1 Hz, 1H), 7.38 (d, *J* = 3.4 Hz, 1H), 7.13 (dd, *J* = 5.1, 3.7 Hz, 1H), 4.70 (s, 2H), 3.85 (s, 3H).

^{13}C NMR (100 MHz, Chloroform-d); δ = 168.35, 137.05, 134.39, 133.24, 130.51, 127.79, 127.18, 58.04, 52.24.

III-22b: Methyl (*E*)-2-(hydroxymethyl)-4-methylpent-2-enoate

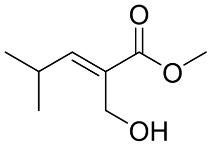

Colorless oil

^{1}H NMR (400 MHz, Chloroform-d); δ = 6.69 (d, *J* = 10.1 Hz, 1H), 4.34 (s, 2H), 3.78 (s, 3H), 1.80 – 1.70 (m, 1H), 1.05 (d, *J* = 6.6 Hz, 6H).

^{13}C NMR (100 MHz, Chloroform-d); δ = 168.28, 152.29, 128.57, 57.45, 51.87, 27.72,

22.37.

III-23b: Methyl (*E*)-2-(hydroxymethyl)-5-methylhex-2-enoate

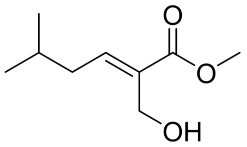

Colorless oil

^{1}H NMR (400 MHz, Chloroform-d); δ = 6.92 (t, *J* = 7.9 Hz, 1H), 4.32 (s, 2H), 3.77 (s, 3H), 2.18 (dd, *J* = 7.9, 6.9 Hz, 2H), 1.80 – 1.70 (m, 1H), 0.94 (d, *J* = 6.7 Hz, 6H).

^{13}C NMR (100 MHz, Chloroform-d); δ = 168.01, 144.93, 131.31, 57.26, 51.85, 37.24, 28.31, 22.38.

III-24b: Methyl (*E*)-2-(hydroxymethyl)hept-2-enoate

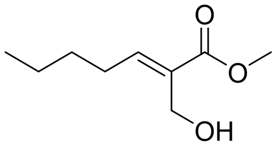

Colorless oil

^{1}H NMR (400 MHz, Chloroform-d); δ = 7.00 (t, *J* = 7.8 Hz, 1H), 4.32 (s, 2H), 3.76 (s, 3H), 2.29 (q, *J* = 7.5 Hz, 2H), 1.46 – 1.30 (m, 4H), 0.91 (t, *J* = 7.2 Hz, 3H).

^{13}C NMR (100 MHz, Chloroform-d); δ = 168.08, 146.14, 130.72, 57.22, 51.88, 30.87, 28.07, 22.37, 13.83.

III-25b: Methyl (*E*)-2-(hydroxymethyl)oct-2-enoate

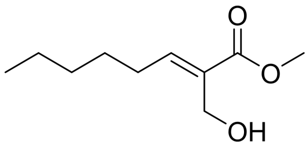

Colorless oil

^{1}H NMR (400 MHz, Chloroform-d); δ = 6.90 (t, *J* = 7.8 Hz, 1H), 4.32 (s, 2H), 3.76 (s, 3H), 2.28 (q, *J* = 7.6 Hz, 2H), 2.28 – 2.22 (m, 2H), 1.47 – 1.30 (m, 2H), 1.30 – 1.27 (m, 4H), 0.89 – 0.85 (m, 3H).

^{13}C NMR (100 MHz, Chloroform-d); δ = 168.09, 146.20, 130.69, 57.22, 51.88, 31.43, 28.46, 28.33, 22.43, 13.96.

III-26b: Methyl (*E*)-3-cyclohexyl-2-(hydroxymethyl)acrylate

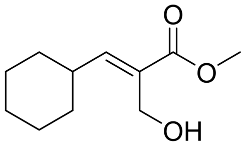

Colorless oil

^{1}H NMR (400 MHz, Chloroform-d); δ = 6.70 (d, *J* = 10.0 Hz, 1H), 4.31 (s, 2H), 3.75

(s, 3H), 2.56 (s, 1H), 2.47 – 2.41 (m, 1H), 1.74 – 1.61 (m, 4H), 1.34 – 1.10 (m, 6H).

^{13}C NMR (100 MHz, Chloroform-d); δ = 168.28, 150.86, 128.95, 57.38, 51.79, 37.39, 32.26, 25.59, 25.28.

III-27b: Methyl (*E*)-3-cyclopropyl-2-(hydroxymethyl)acrylate

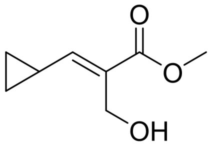

Colorless oil

^{1}H NMR (400 MHz, Chloroform-d); δ = 5.52 (d, *J* = 10.9 Hz, 1H), 4.21 (d, *J* = 0.8 Hz, 2H), 3.81 (s, 3H), 2.68 – 2.59 (m, 1H), 1.00 (dd, *J* = 8.0, 2.3 Hz, 2H), 0.58 (dd, *J* = 4.5, 2.2 Hz, 2H).

^{13}C NMR (100 MHz, Chloroform-d); δ = 167.93, 152.77, 127.67, 65.36, 51.48, 12.24, 9.09.

III-28b: Ethyl (*E*)-2-(hydroxymethyl)-3-phenylacrylate

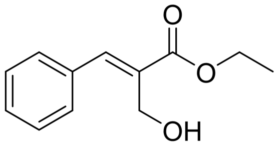

Colorless oil

^{1}H NMR (400 MHz, Chloroform-d); δ = 7.83 (s, 1H), 7.47 – 7.34 (m, 5H), 4.48 (s, 2H), 4.33 (q, *J* = 7.1 Hz, 2H), 2.82 (s, 1H), 1.38 (t, *J* = 7.2 Hz, 3H).

^{13}C NMR (100 MHz, Chloroform-d); δ = 167.92, 142.28, 134.43, 130.98, 129.49, 129.08, 128.47, 61.08, 57.75, 14.22.

5. ^{1}H NMR and ^{13}C NMR spectra of products

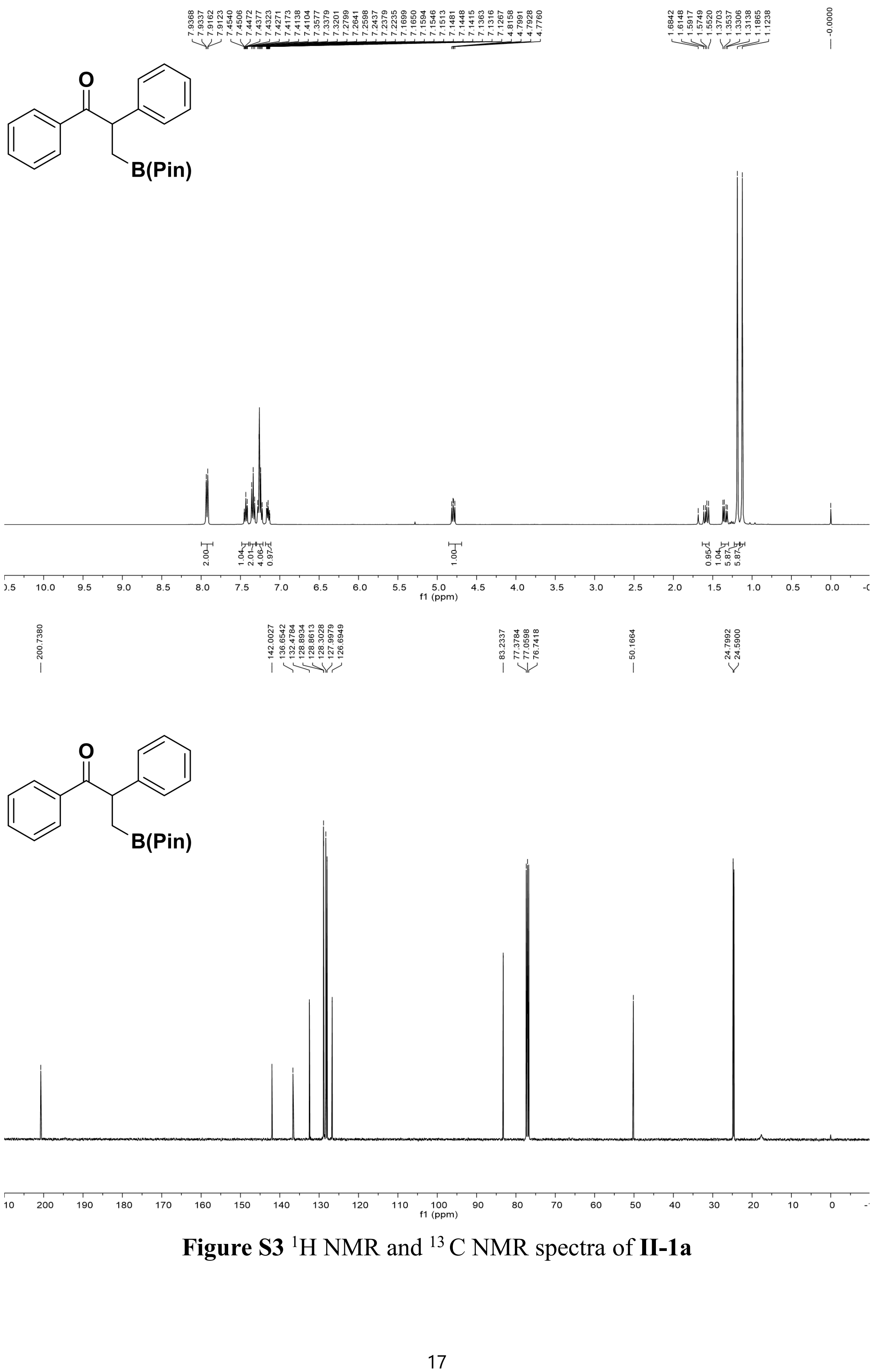

Figure S3 ^{1}H NMR and ^{13}C NMR spectra of **II-1a**

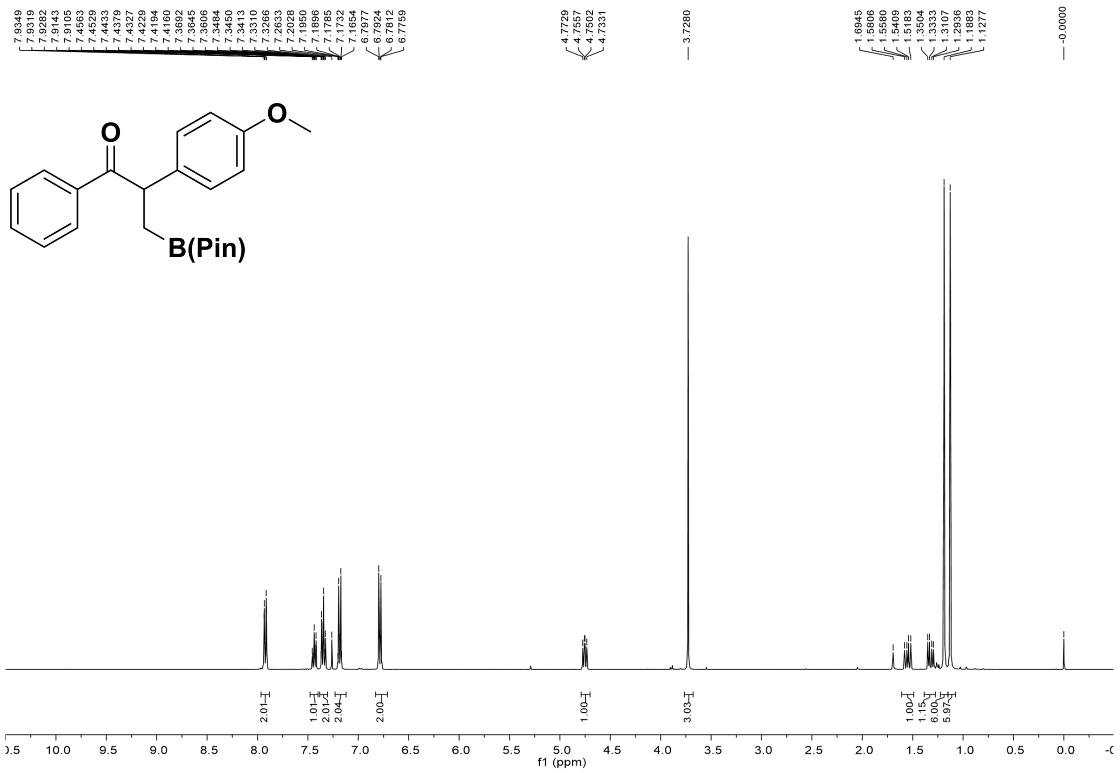

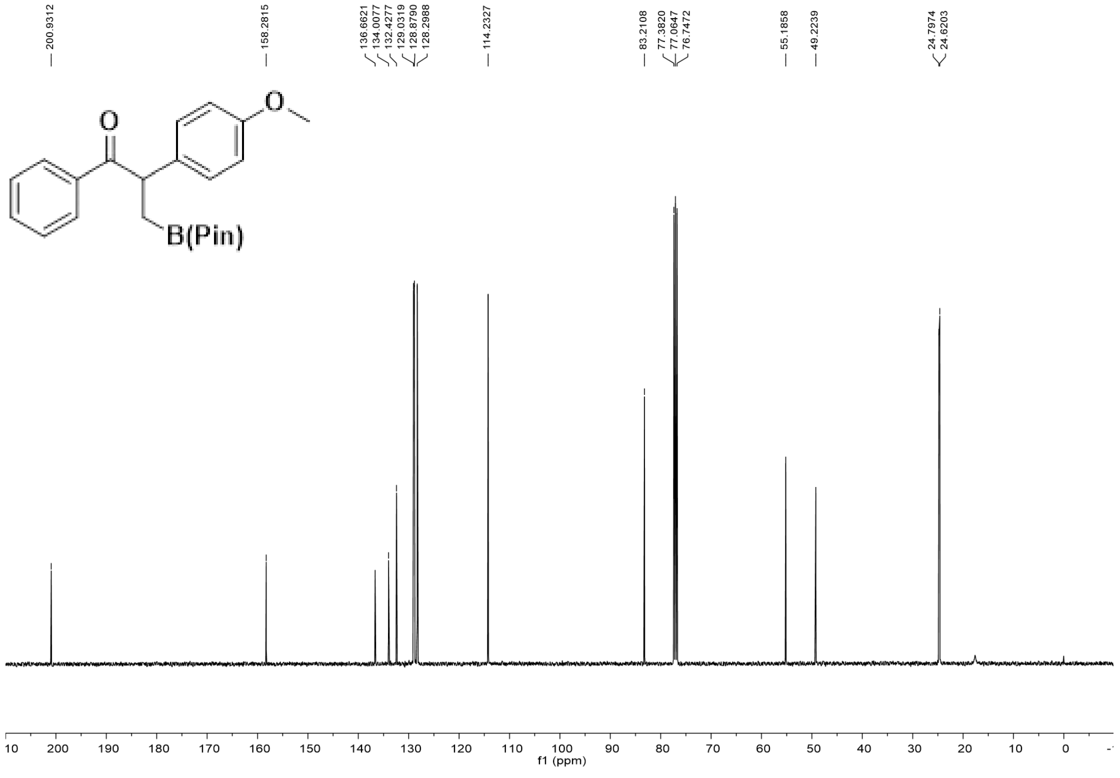

Figure S4 ^{1}H NMR and ^{13}C NMR spectra of **II-2a**

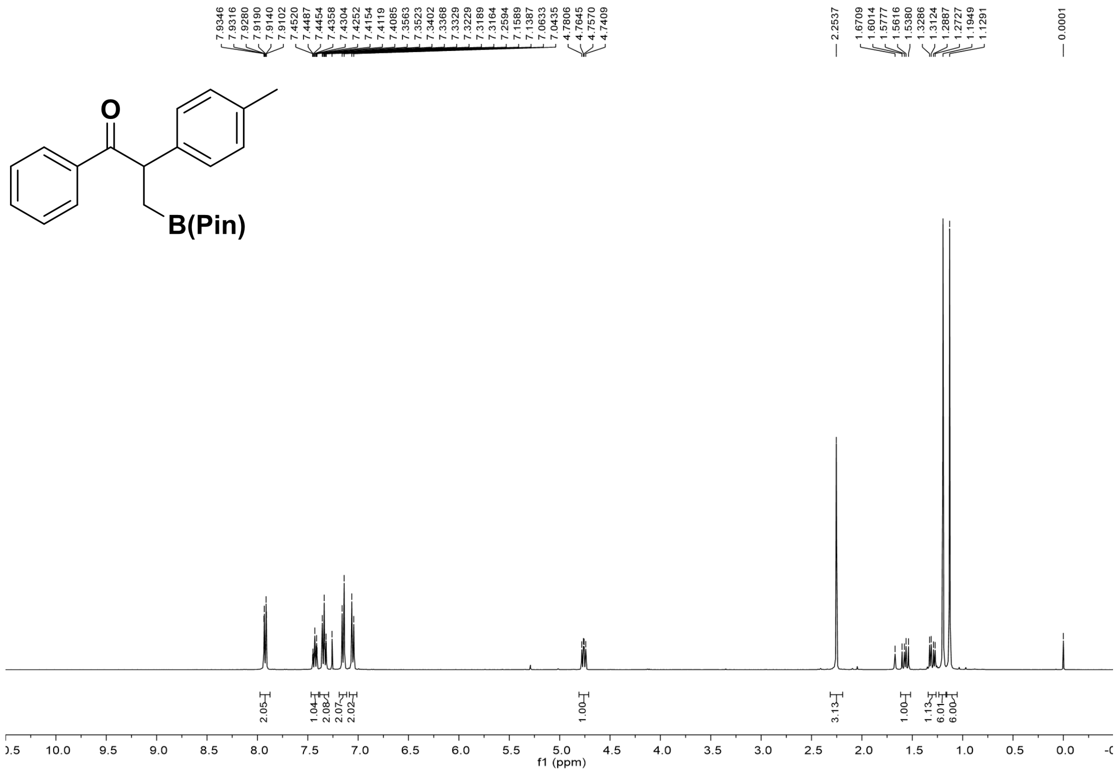

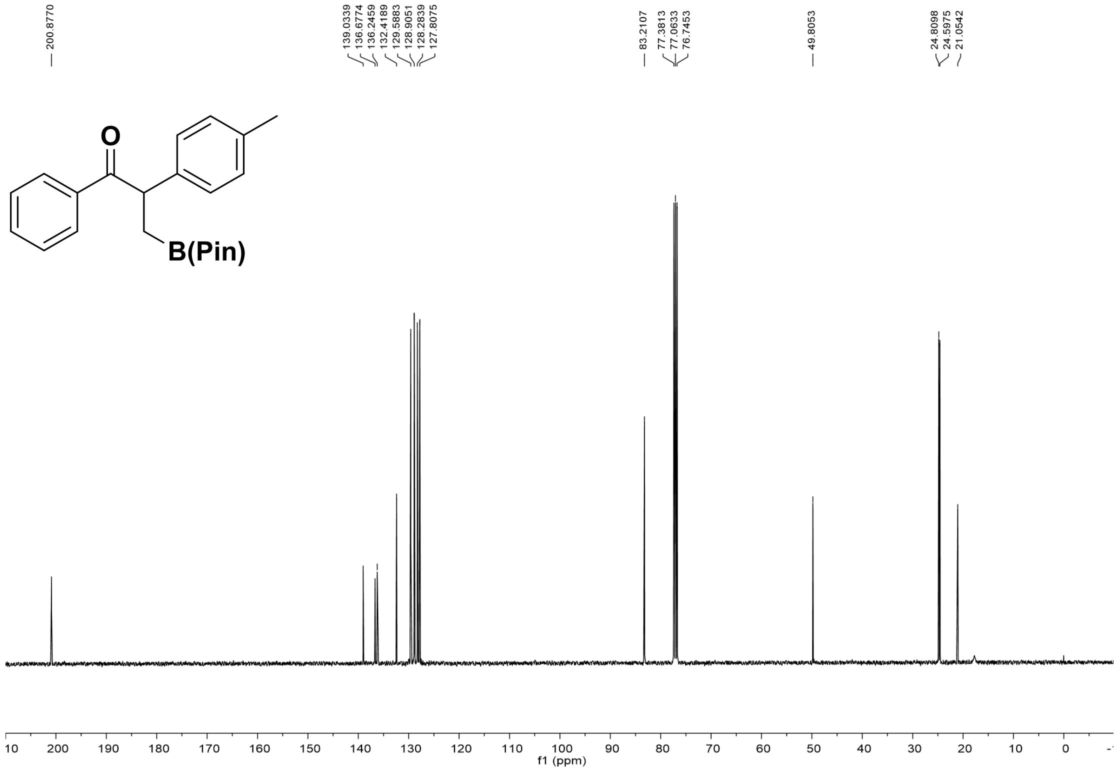

Figure S5 ^{1}H NMR and ^{13}C NMR spectra of **II-3a**

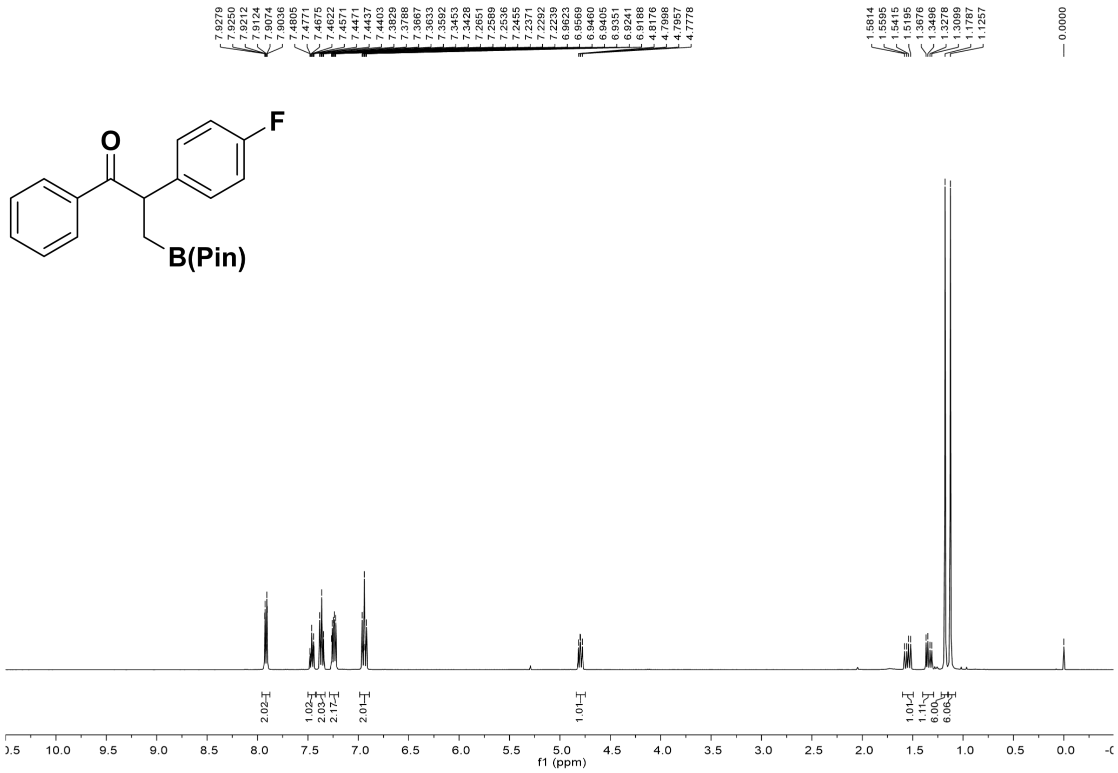

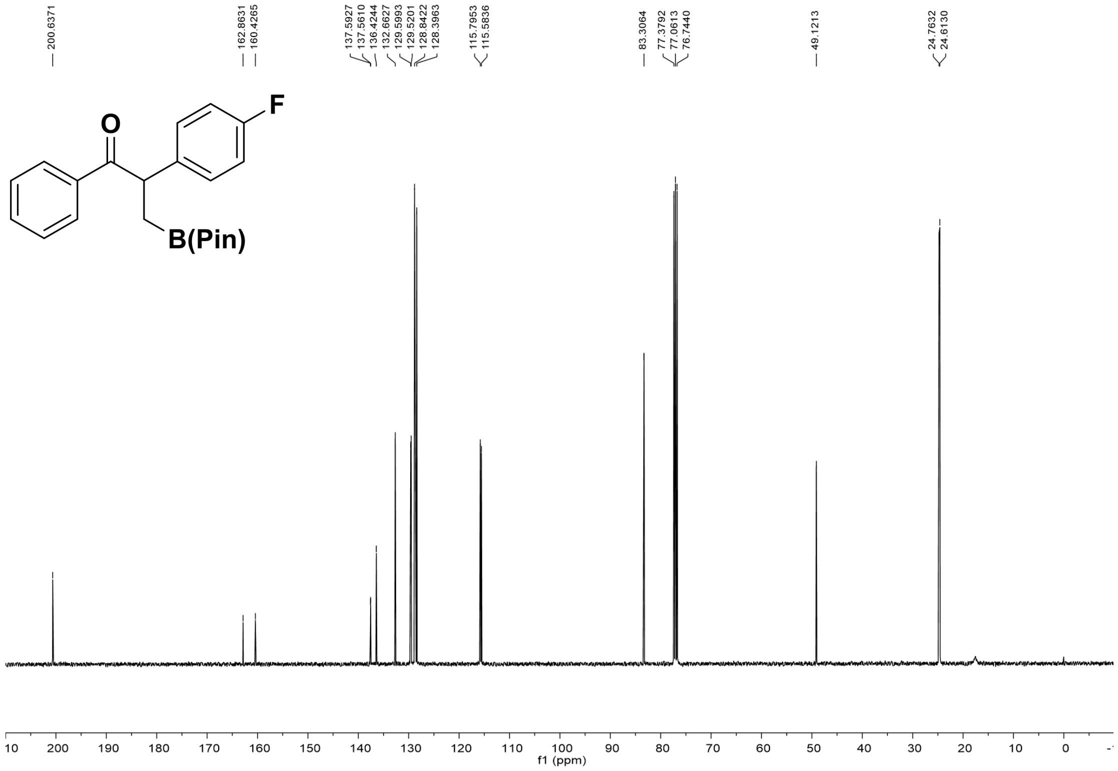

Figure S6 ^{1}H NMR and ^{13}C NMR spectra of **II-4a**

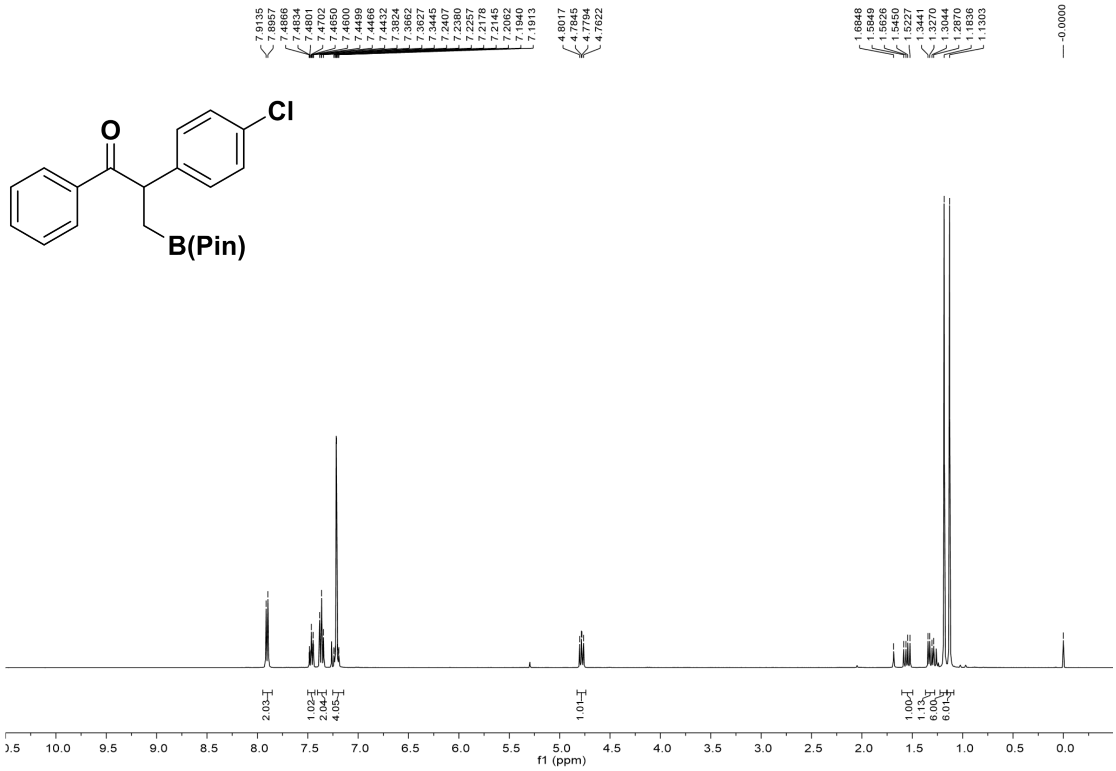

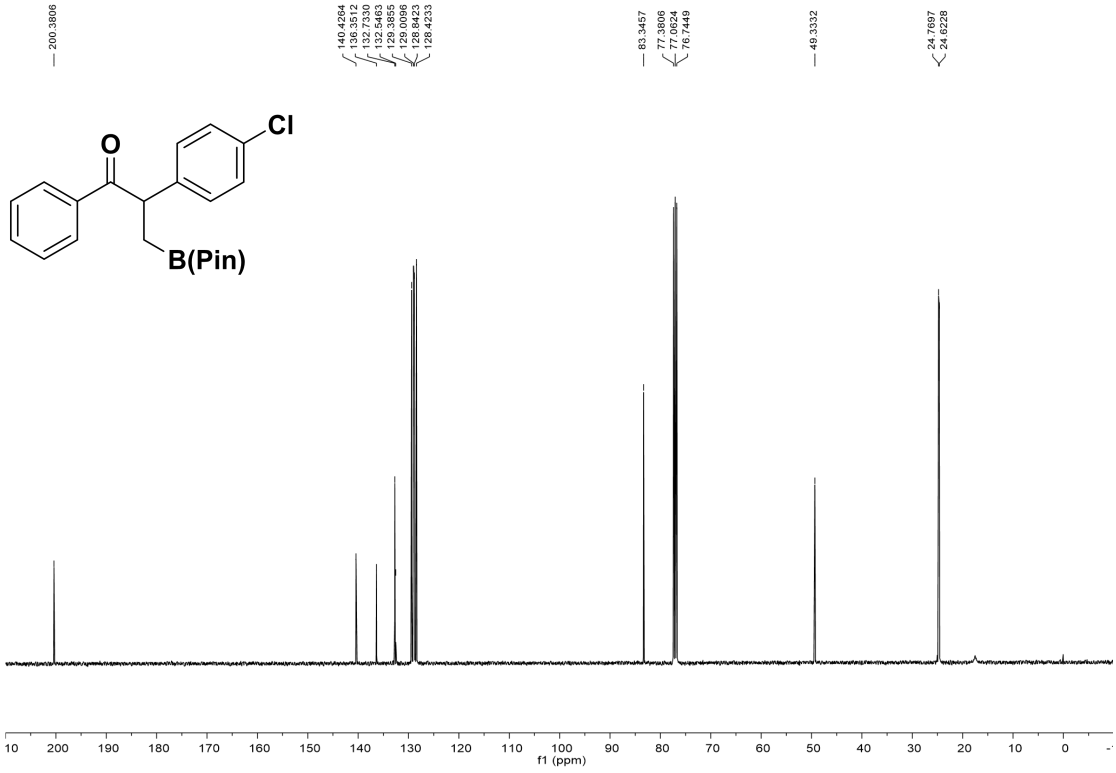

Figure S7 ^{1}H NMR and ^{13}C NMR spectra of **II-5a**

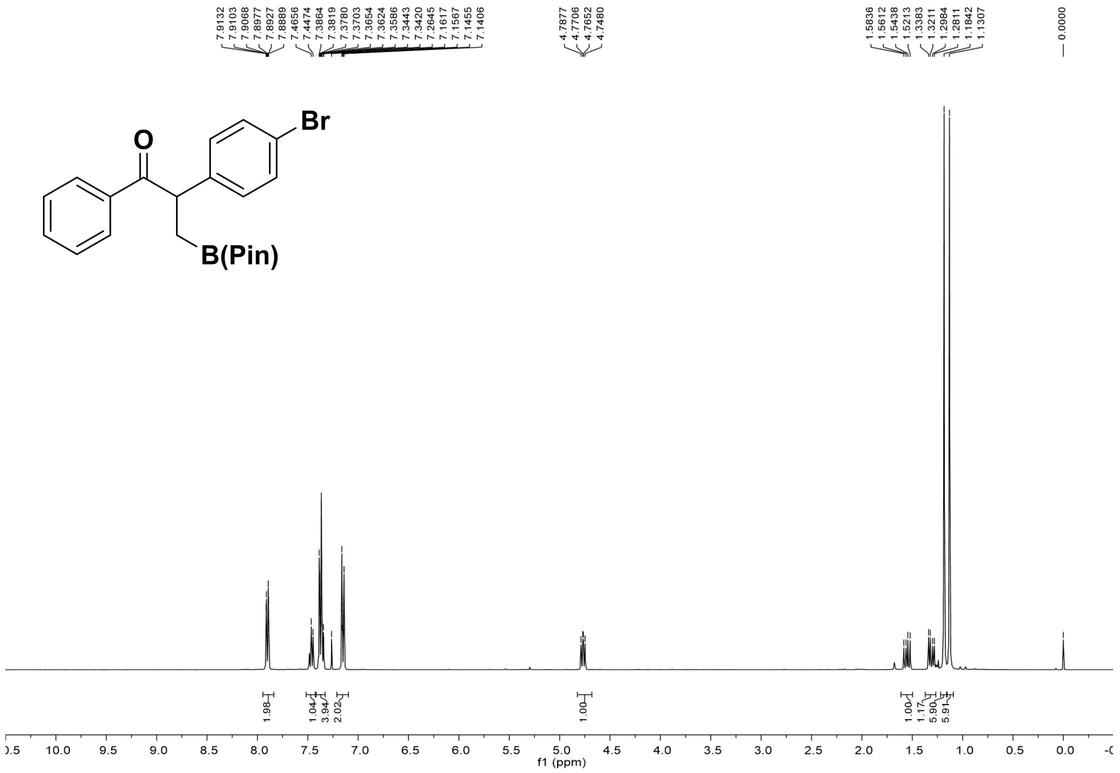

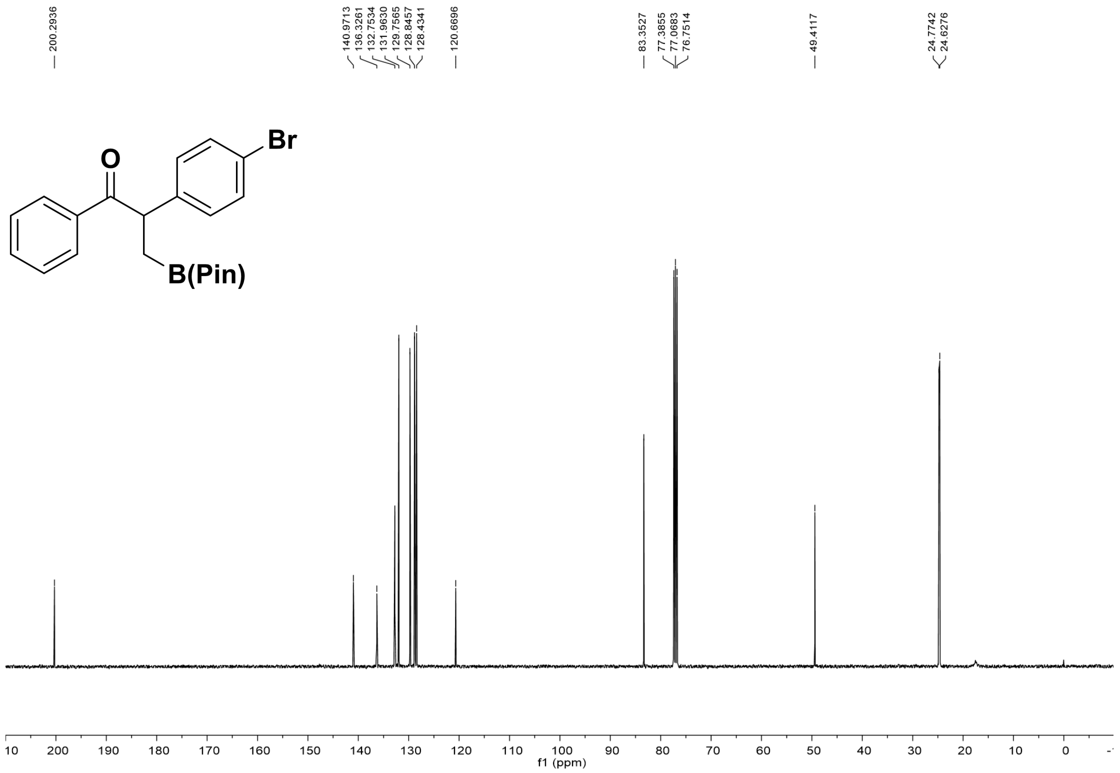

Figure S8 ^{1}H NMR and ^{13}C NMR spectra of **II-6a**

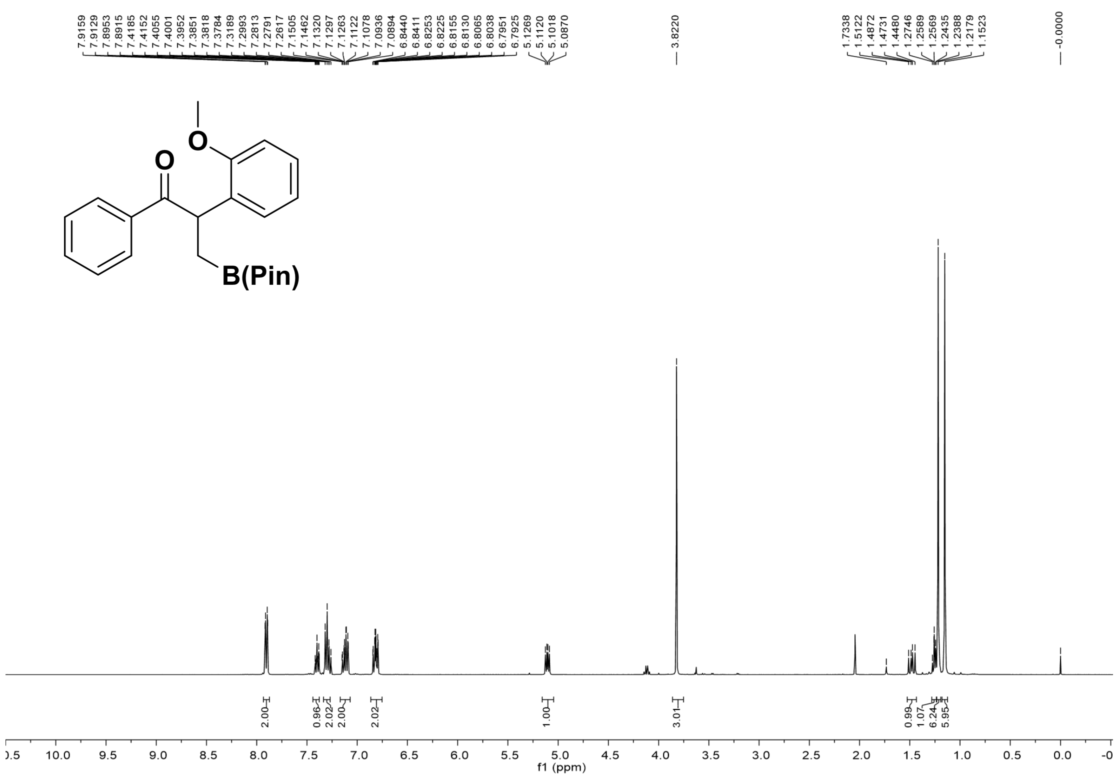

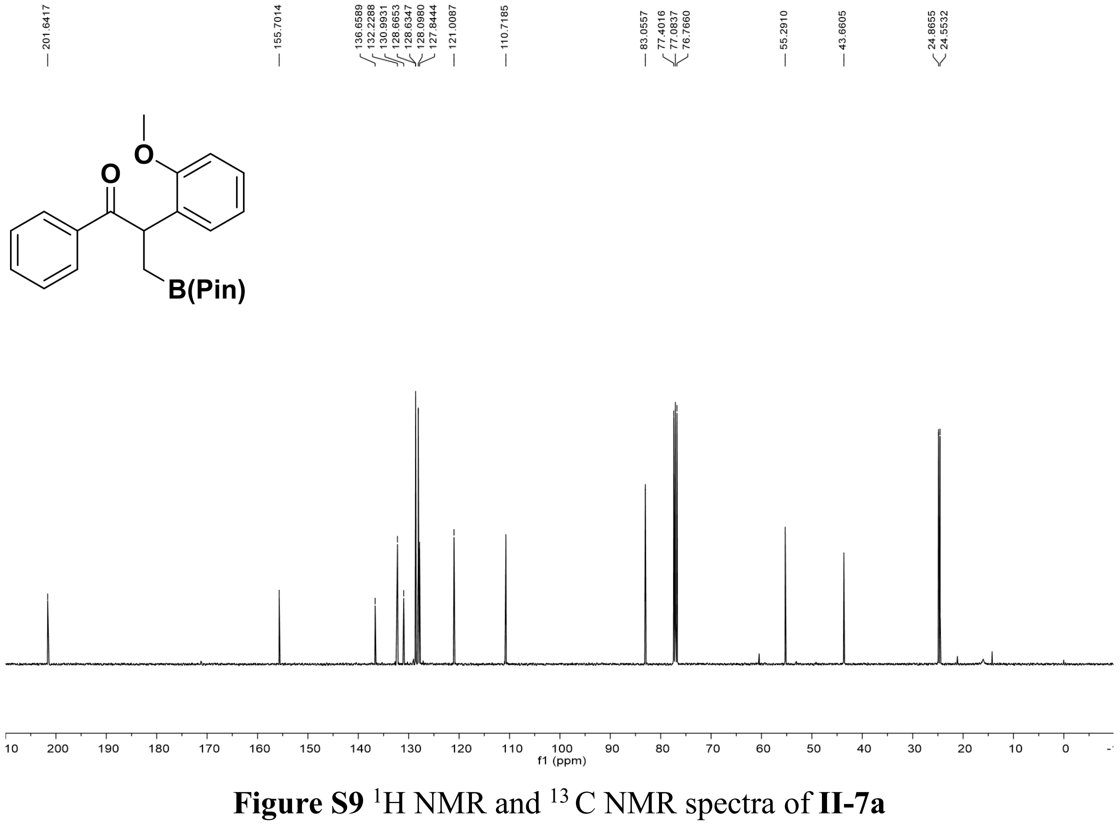

Figure S9 ^{1}H NMR and [13] C NMR spectra of **II-7a**

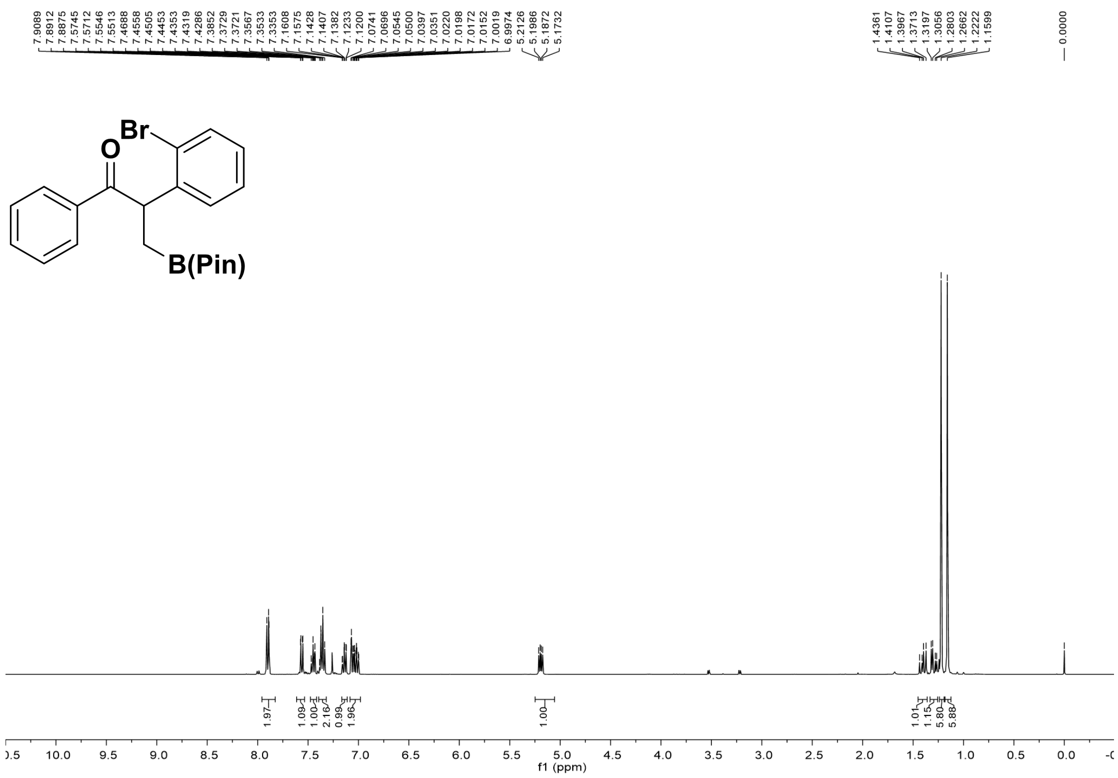

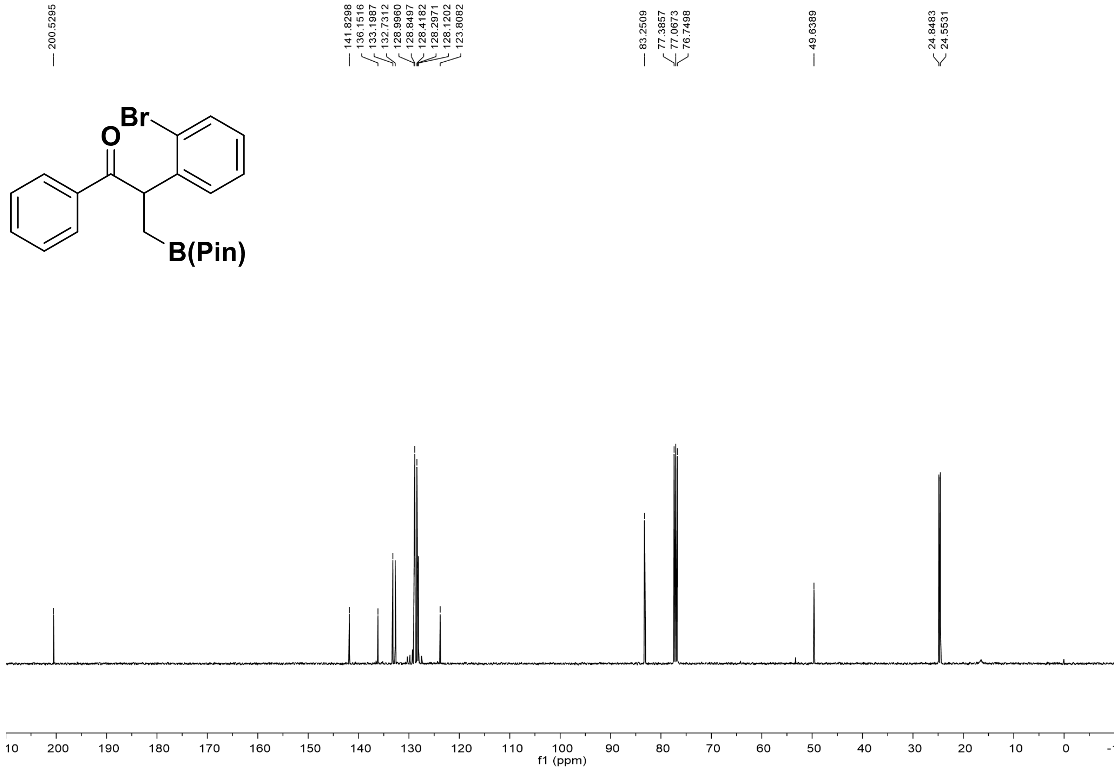

Figure S10 ^{1}H NMR and ^{13}C NMR spectra of **II-8a**

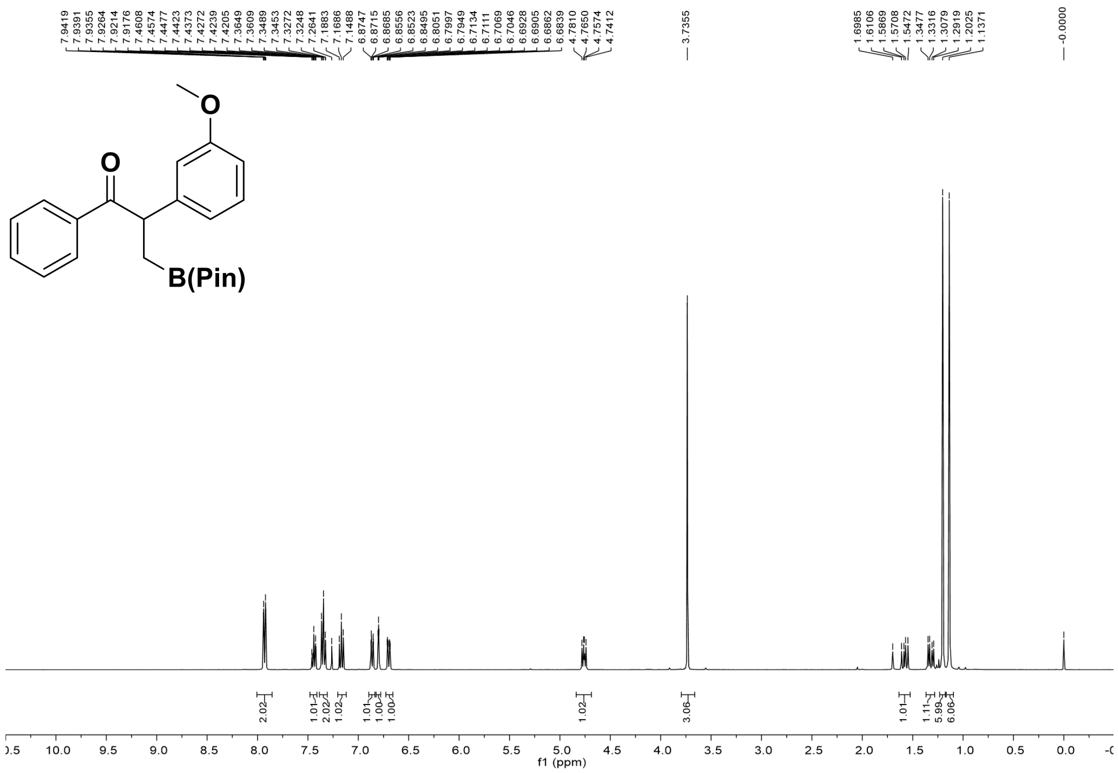

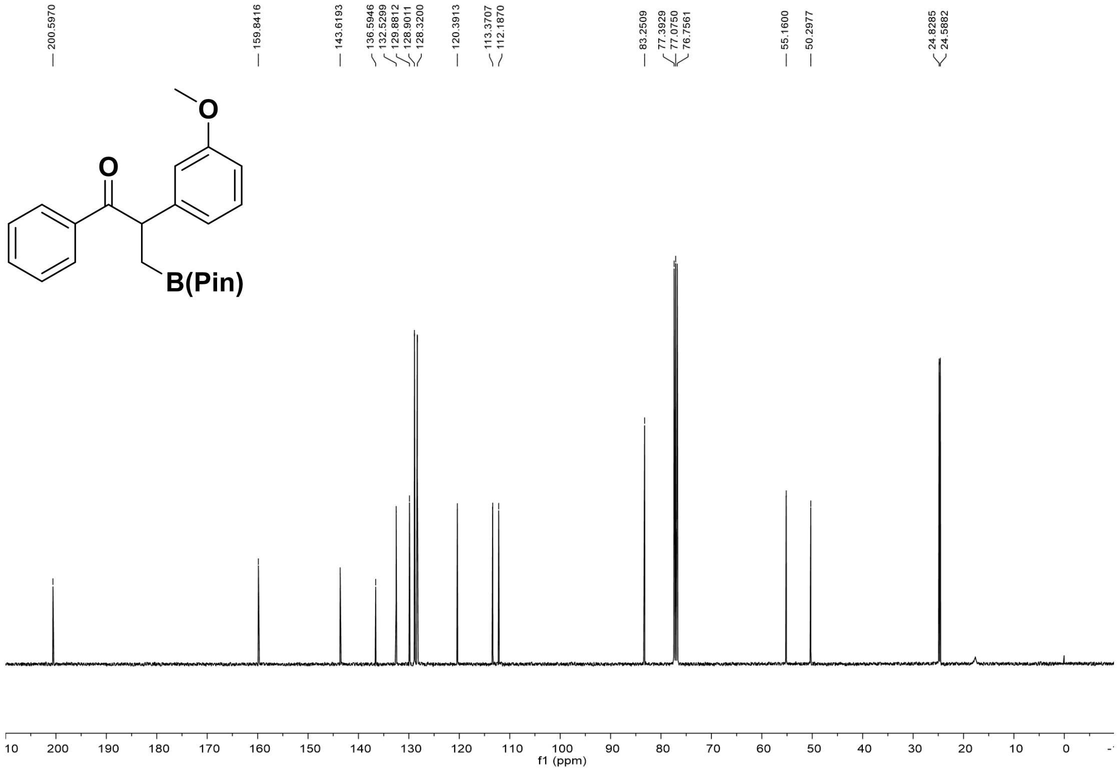

Figure S11 ^{1}H NMR and ^{13}C NMR spectra of **II-9a**

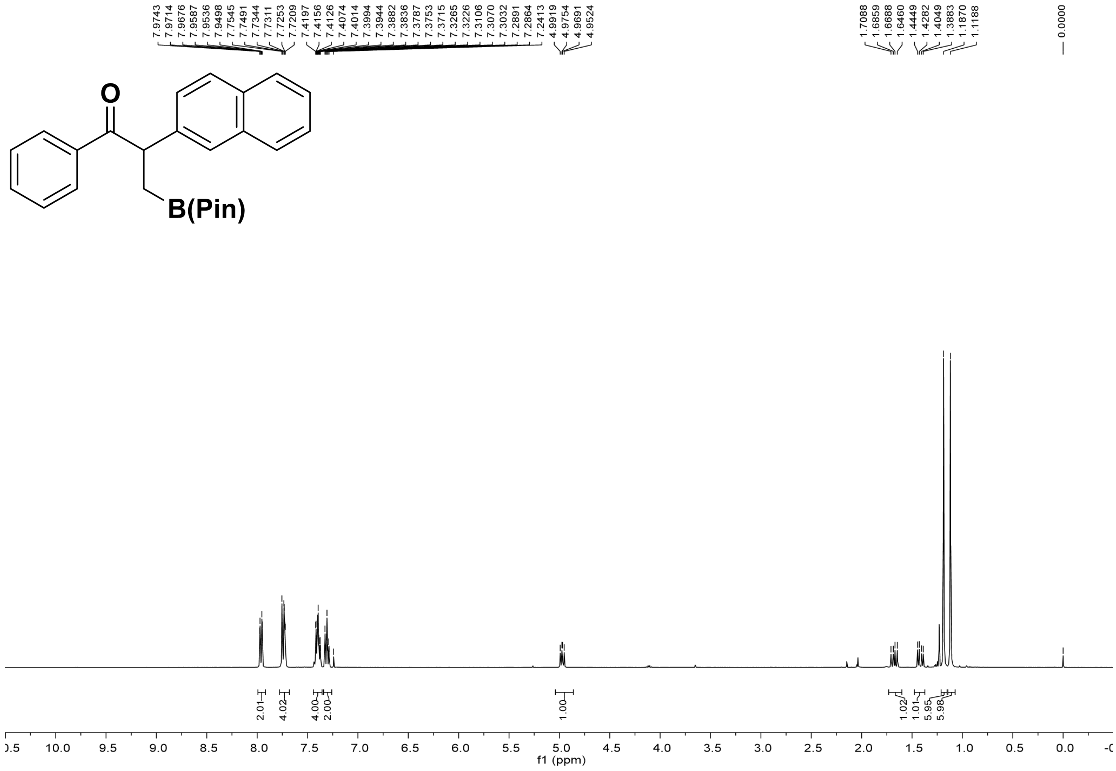

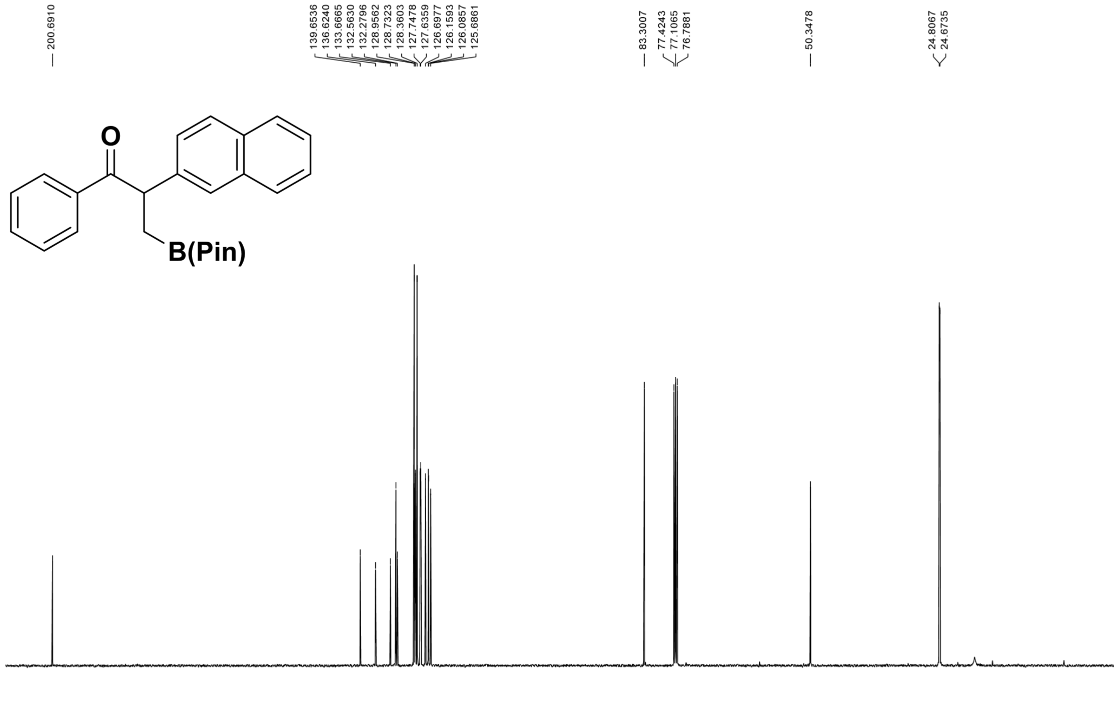

Figure S12 ^{1}H NMR and ^{13}C NMR spectra of **II-10a**

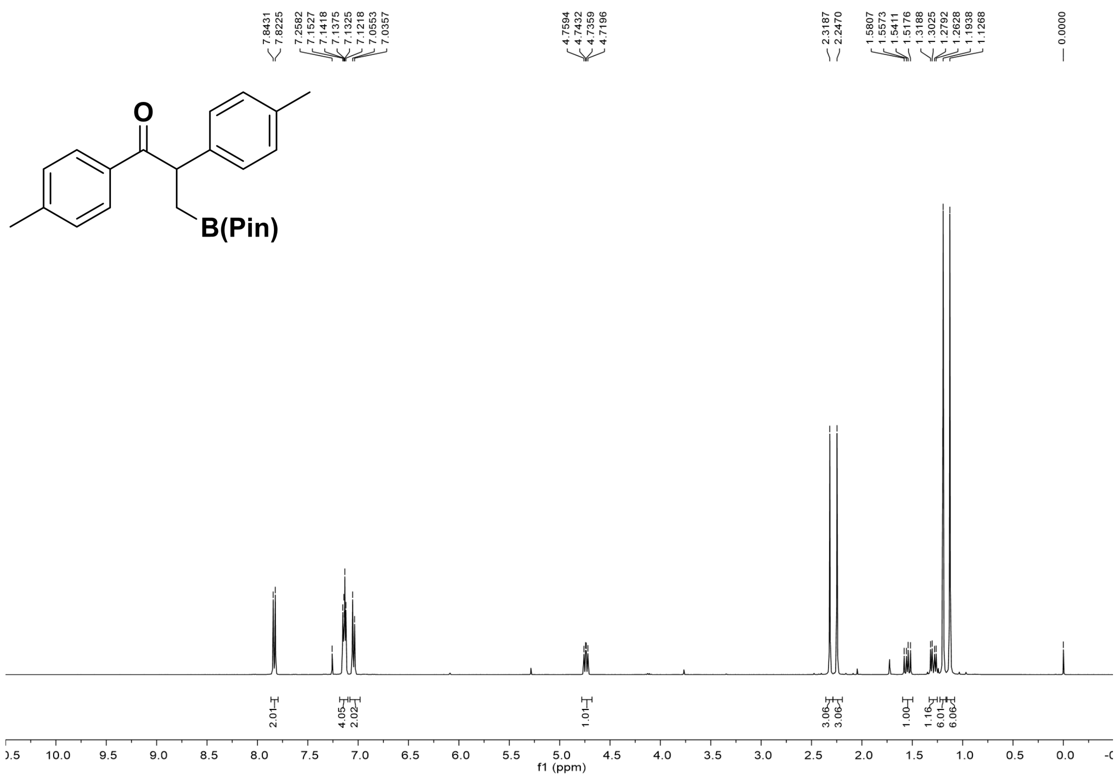

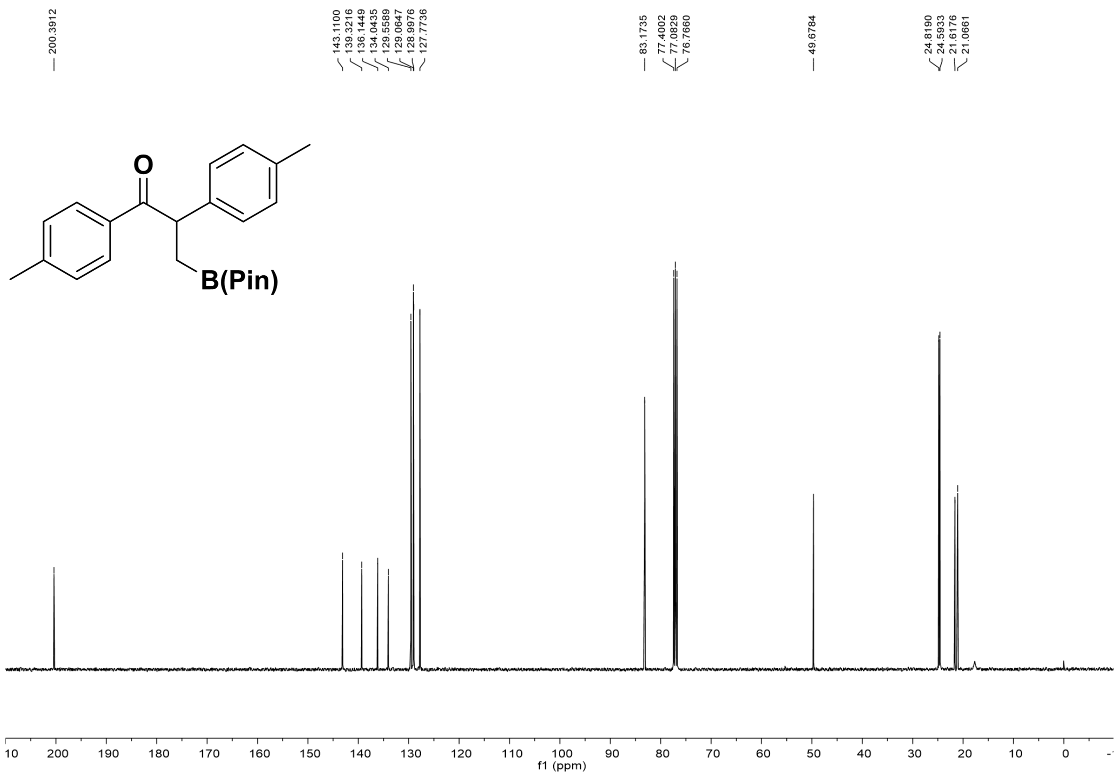

Figure S13 ^{1}H NMR and ^{13}C NMR spectra of **II-11a**

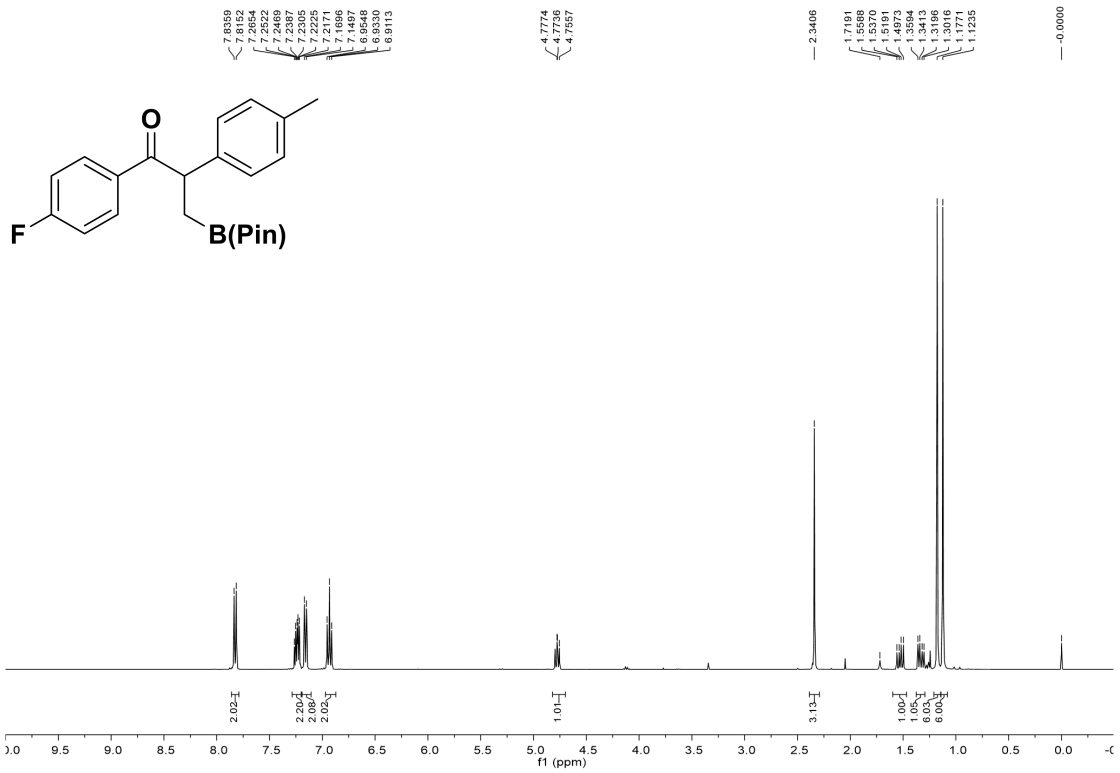

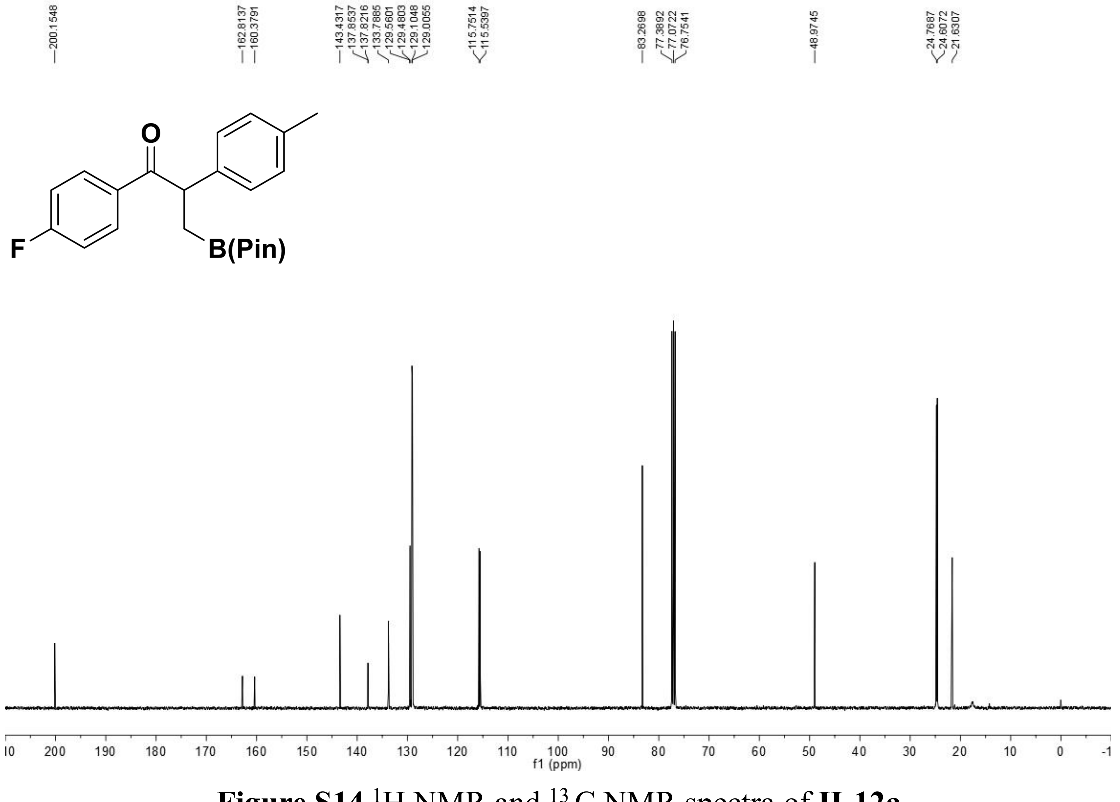

Figure S14 ^{1}H NMR and ^{13}C NMR spectra of **II-12a**

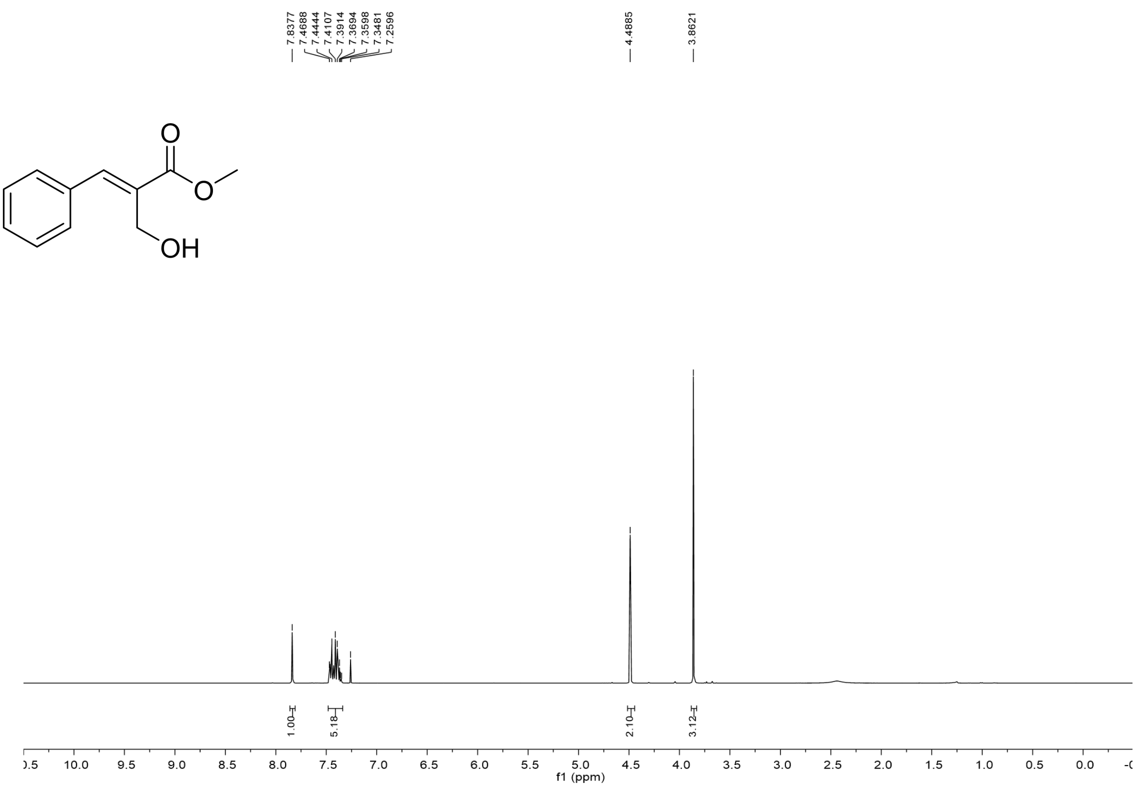

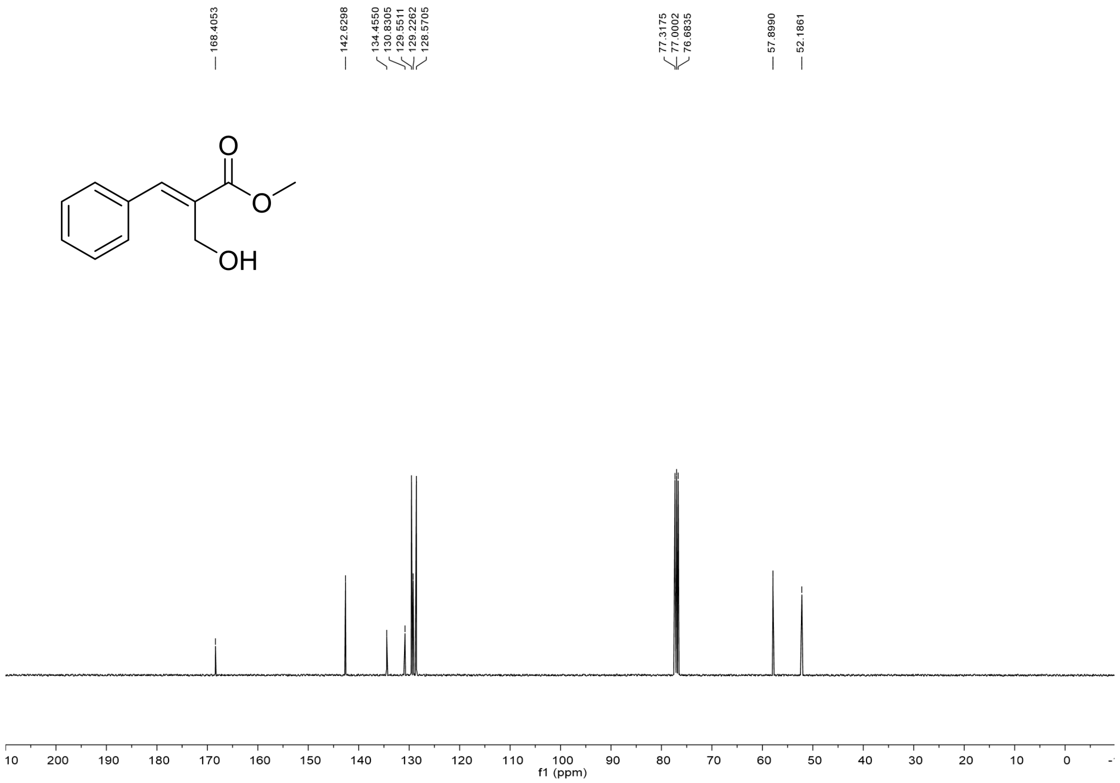

Figure S15 ^{1}H NMR and ^{13}C NMR spectra of **III-1b**

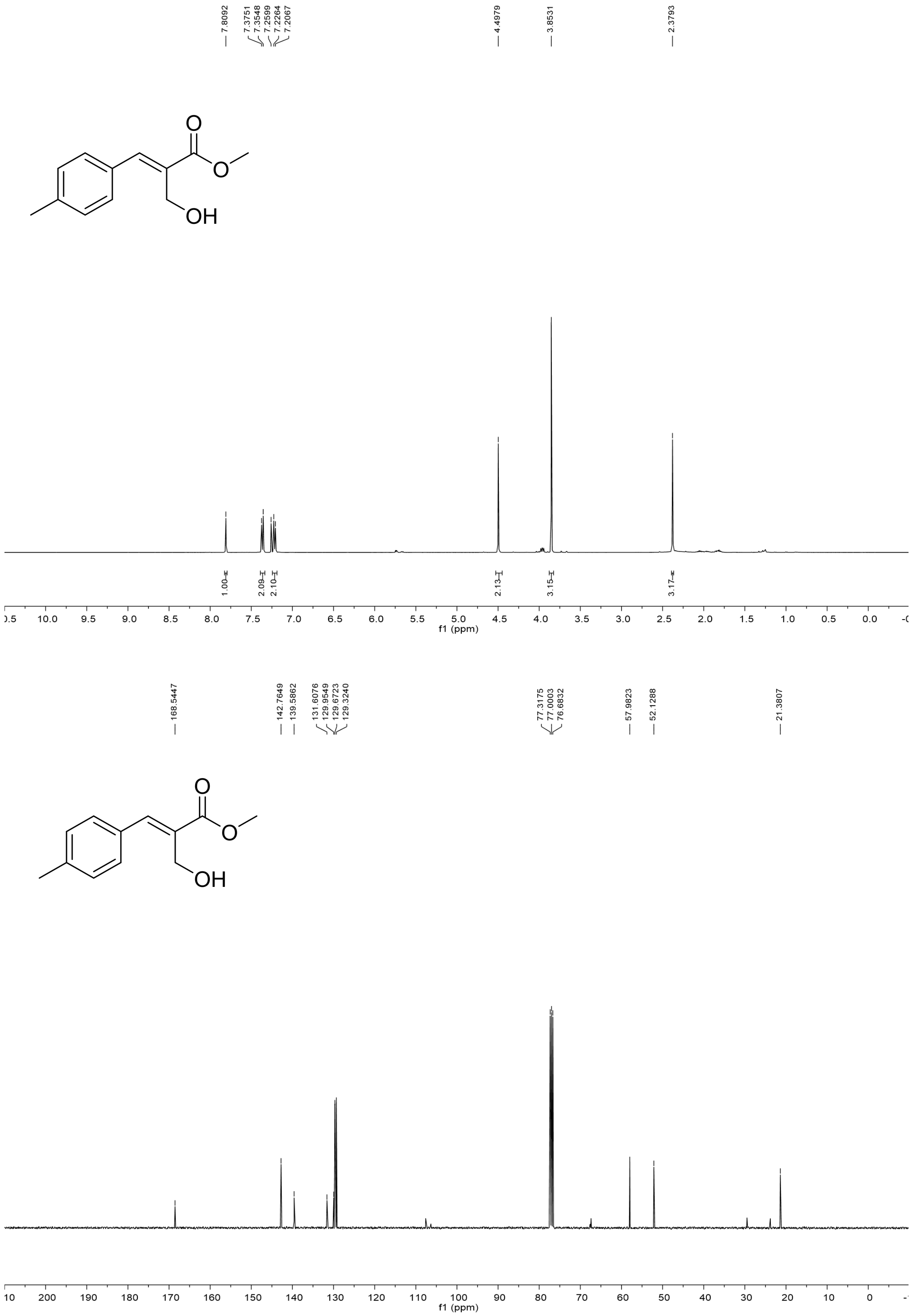

Figure S16 ¹H NMR and ¹³C NMR spectra of **III-2b**

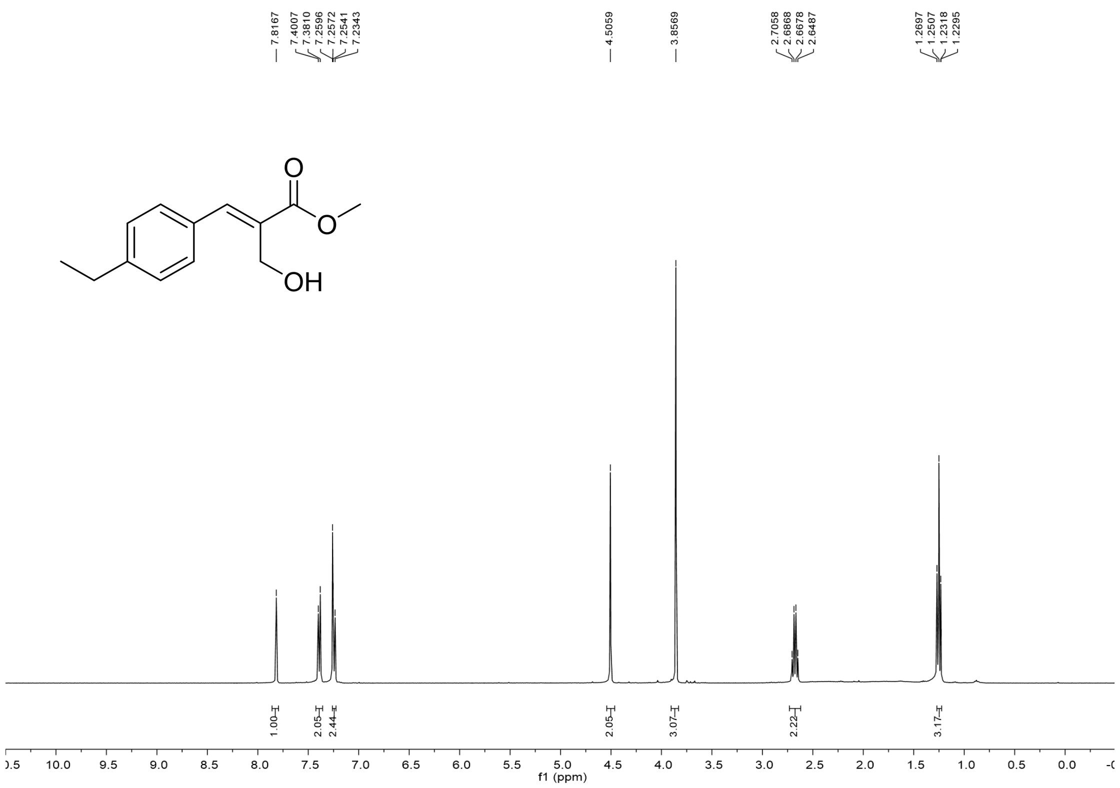

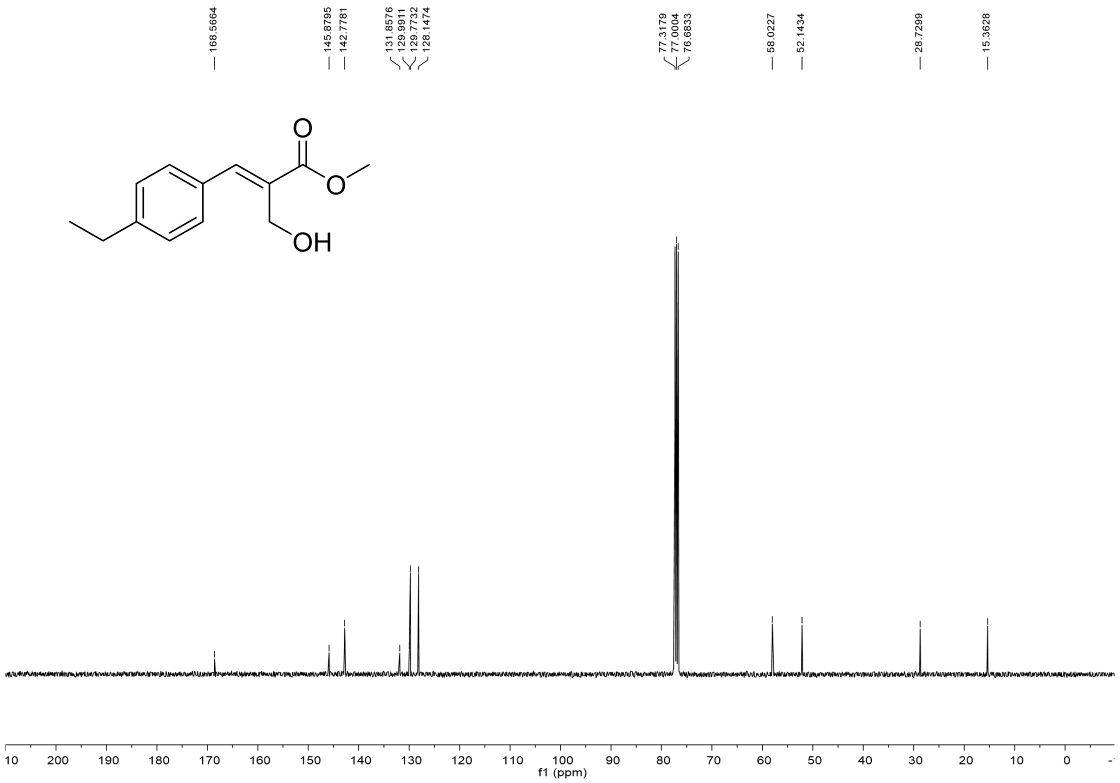

Figure S17 ^{1}H NMR and ^{13}C NMR spectra of **III-3b**

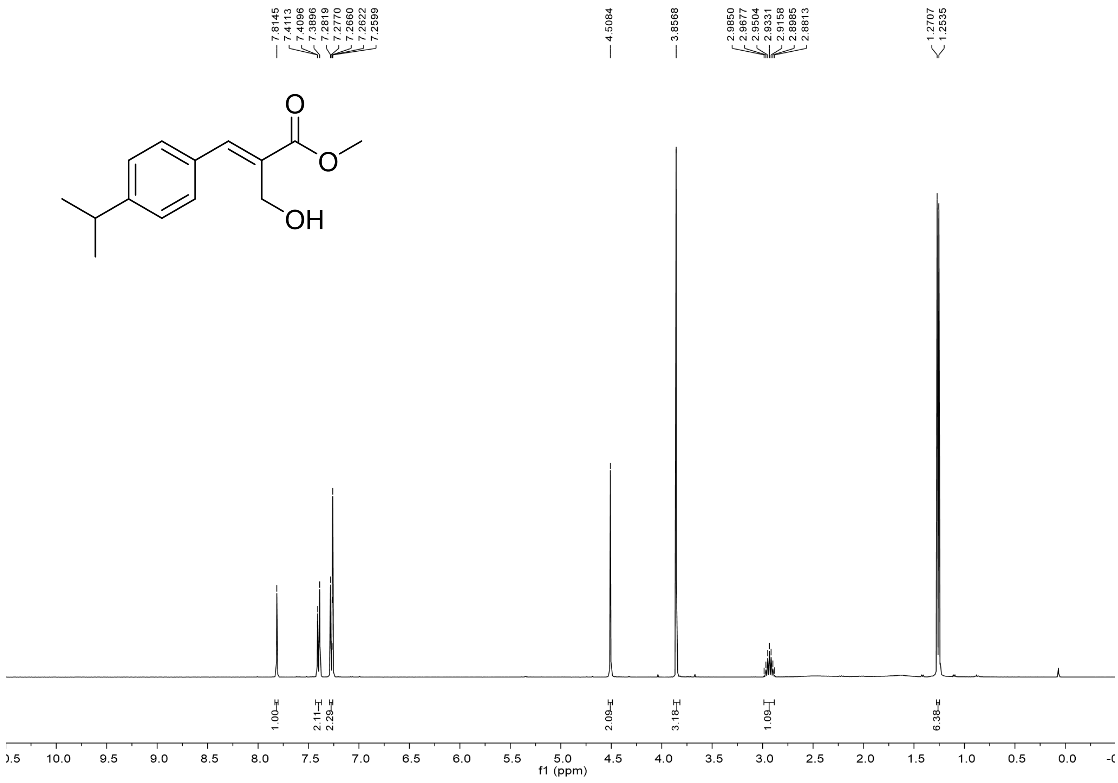

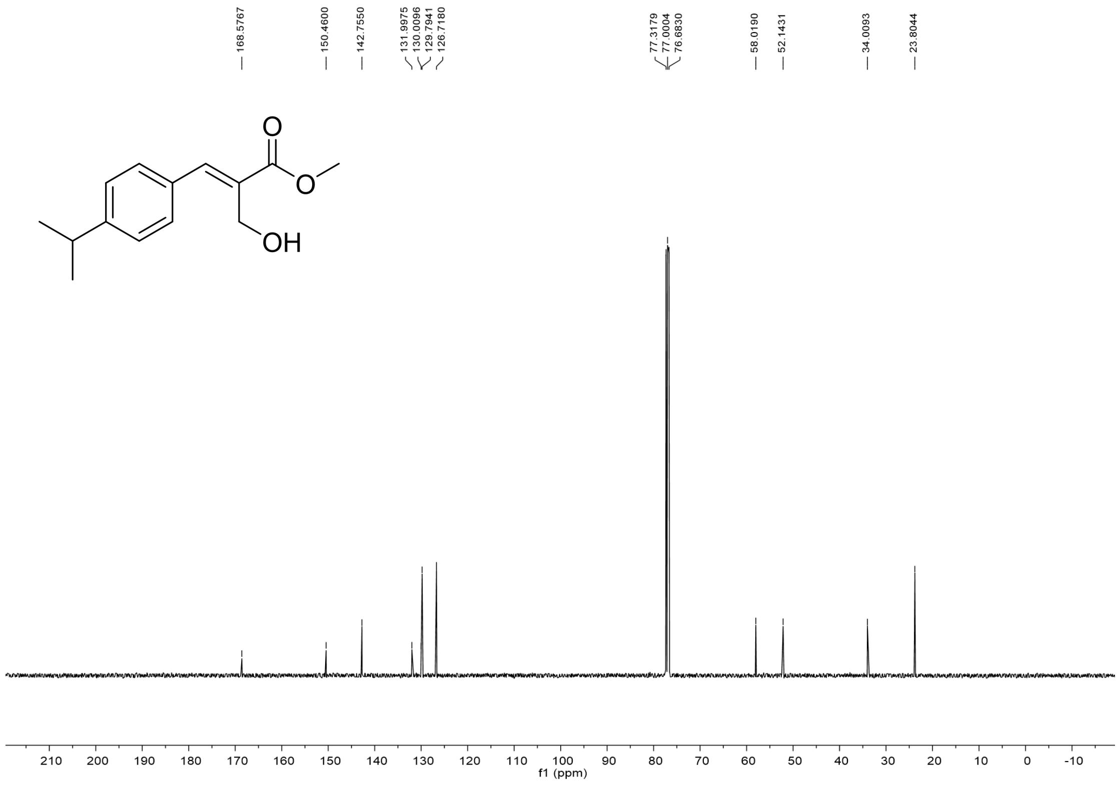

Figure S18 ^{1}H NMR and ^{13}C NMR spectra of **III-4b**

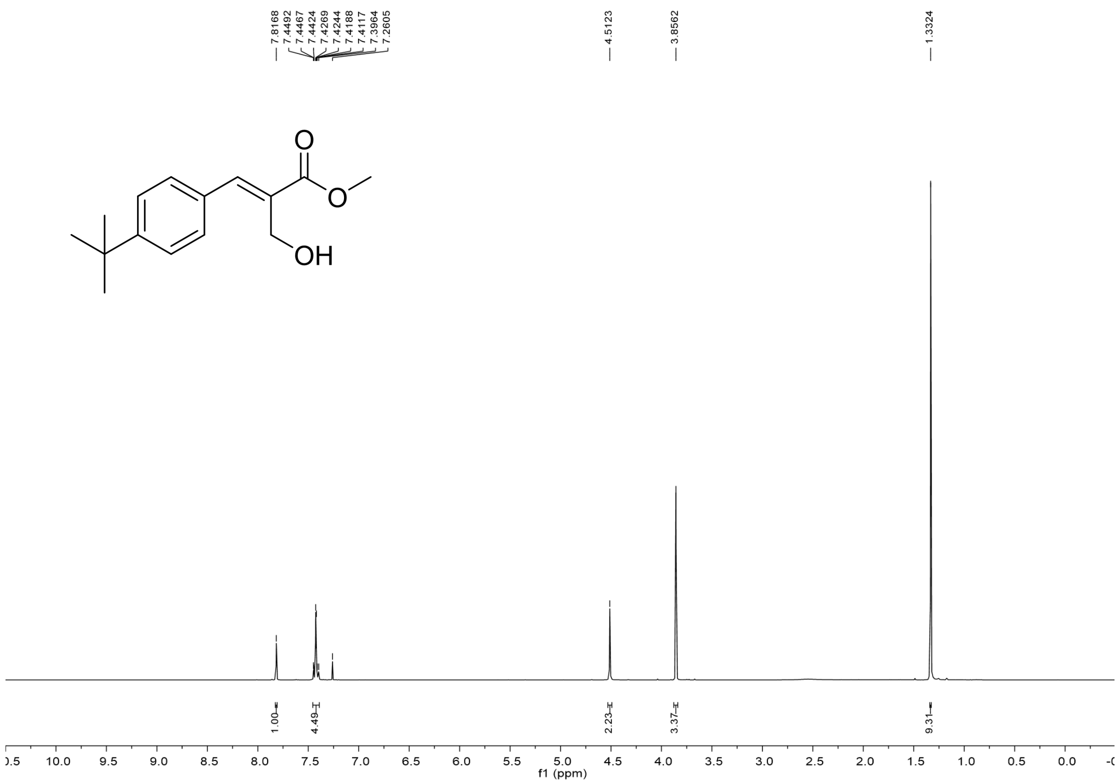

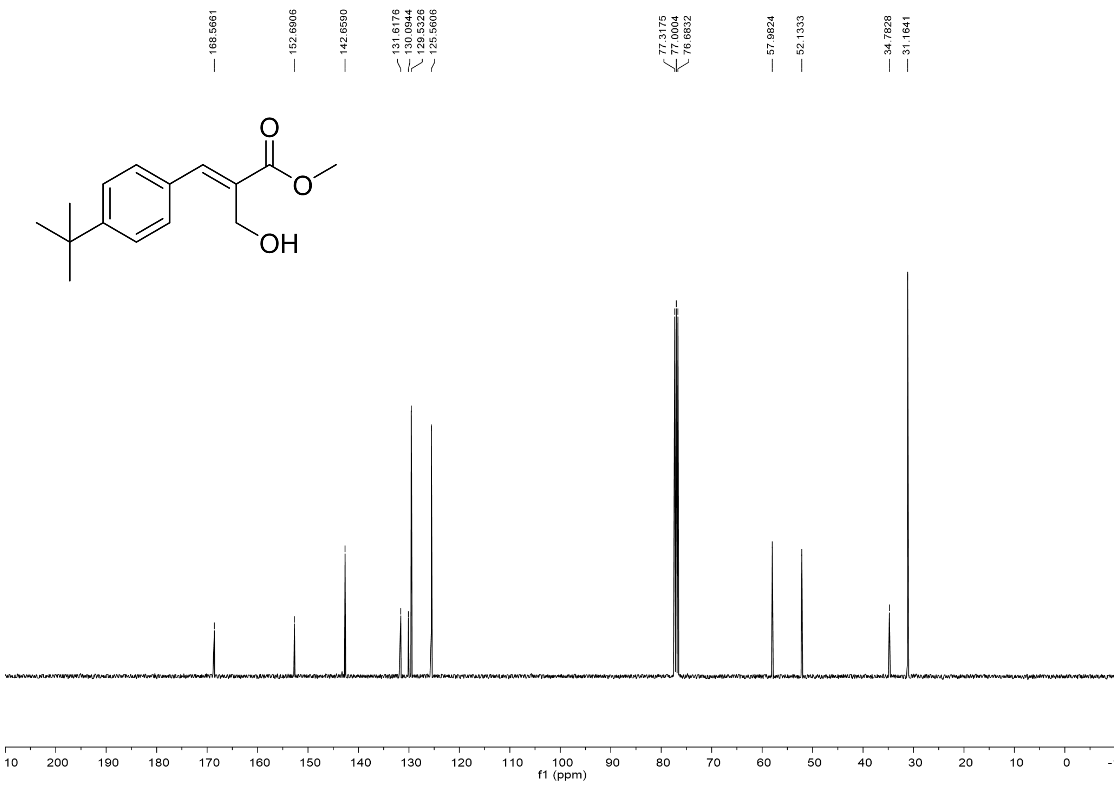

Figure S19 ^{1}H NMR and ^{13}C NMR spectra of **III-5b**

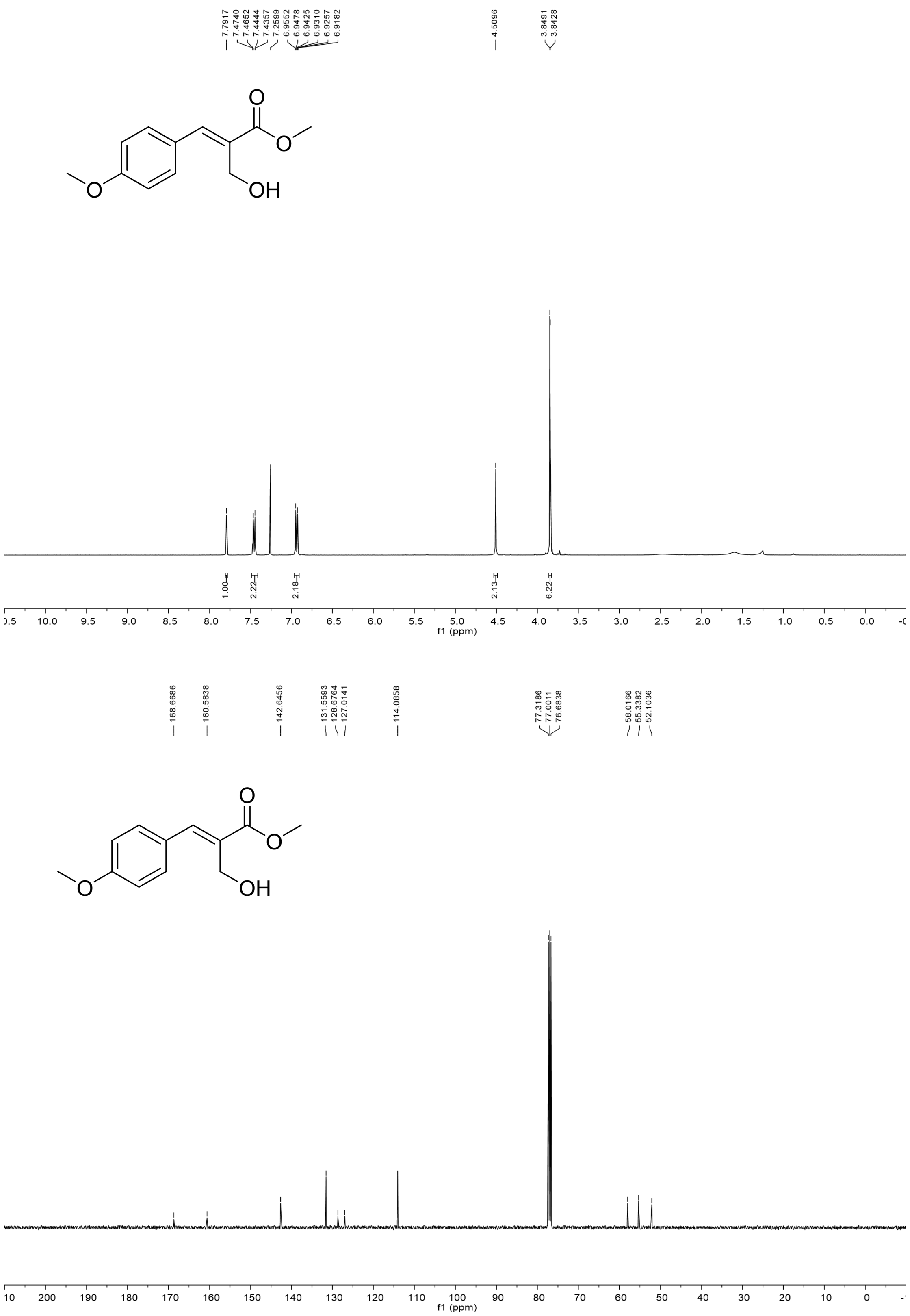

Figure S20 ¹H NMR and ¹³C NMR spectra of **III-6b**

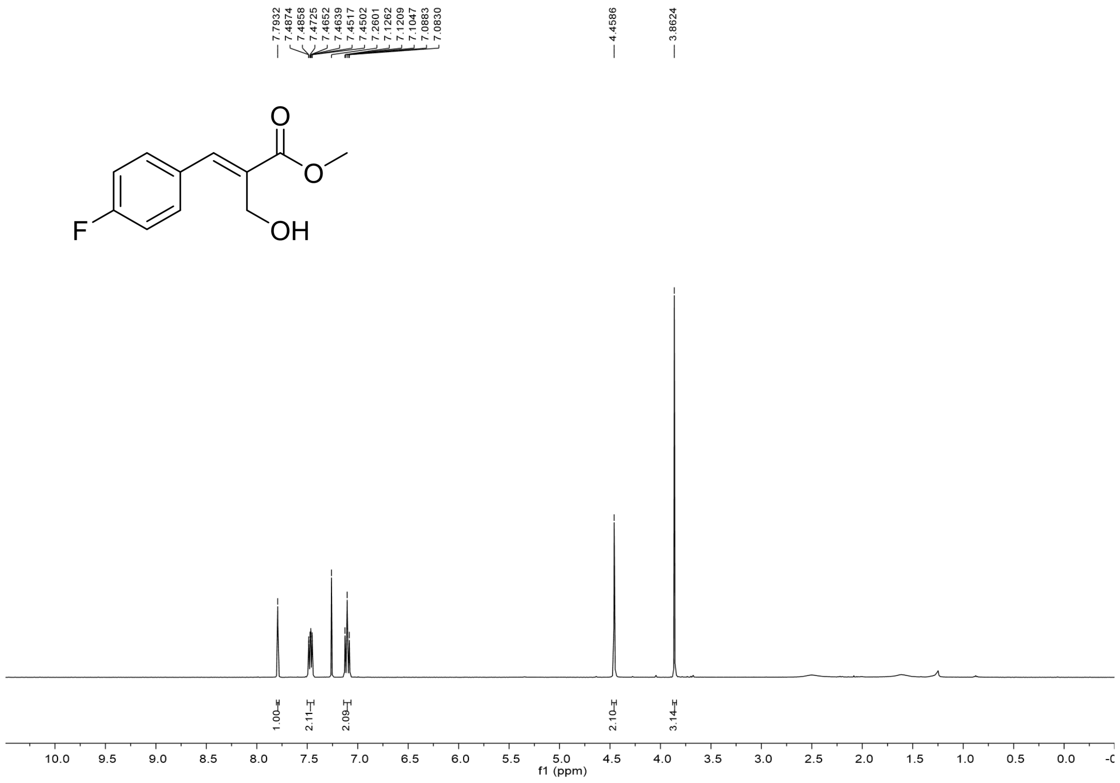

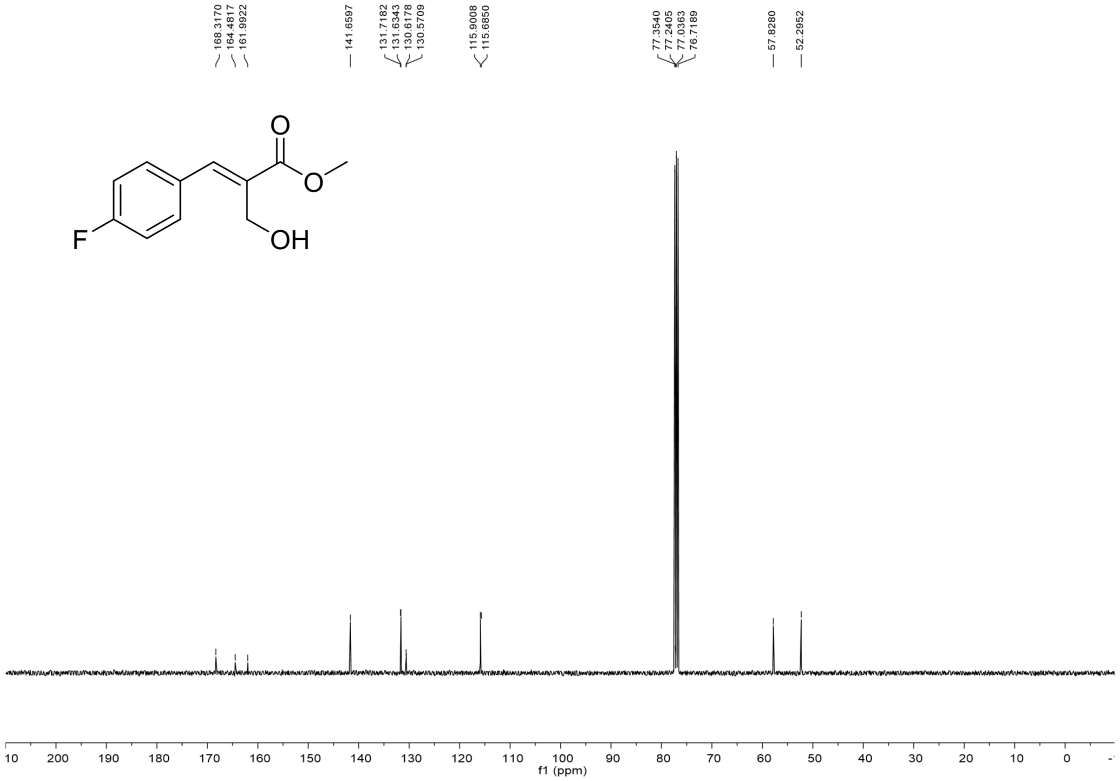

Figure S21 ^{1}H NMR and ^{13}C NMR spectra of **III-7b**

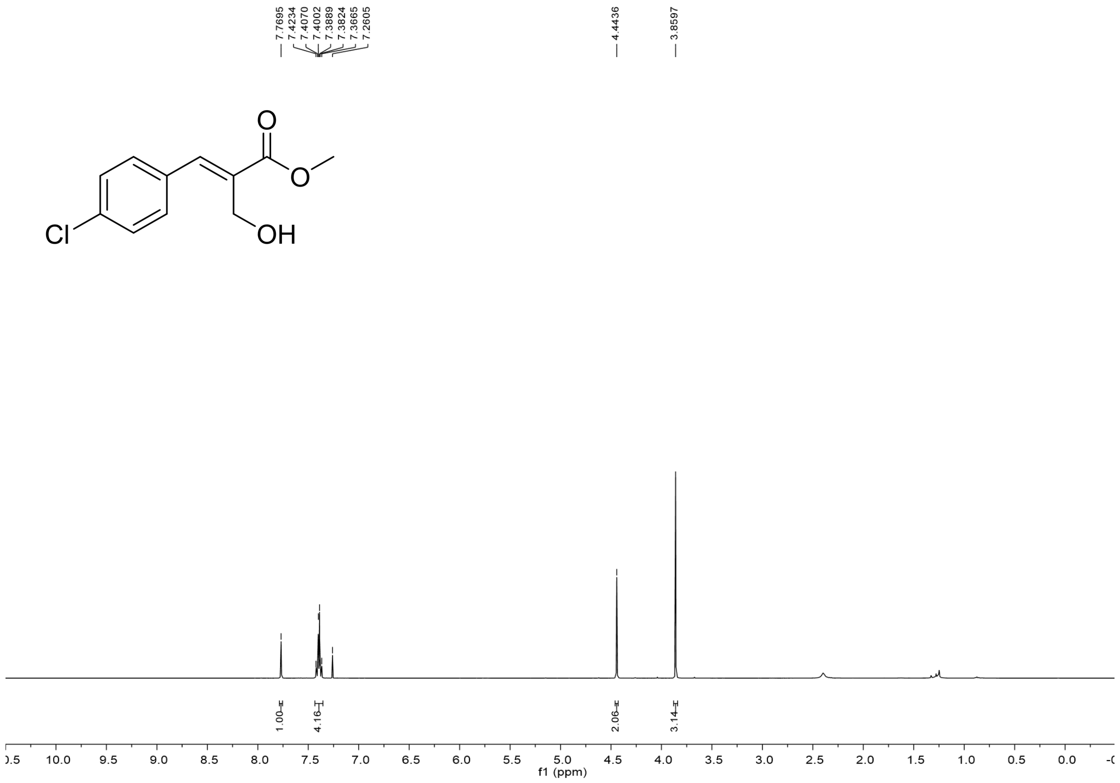

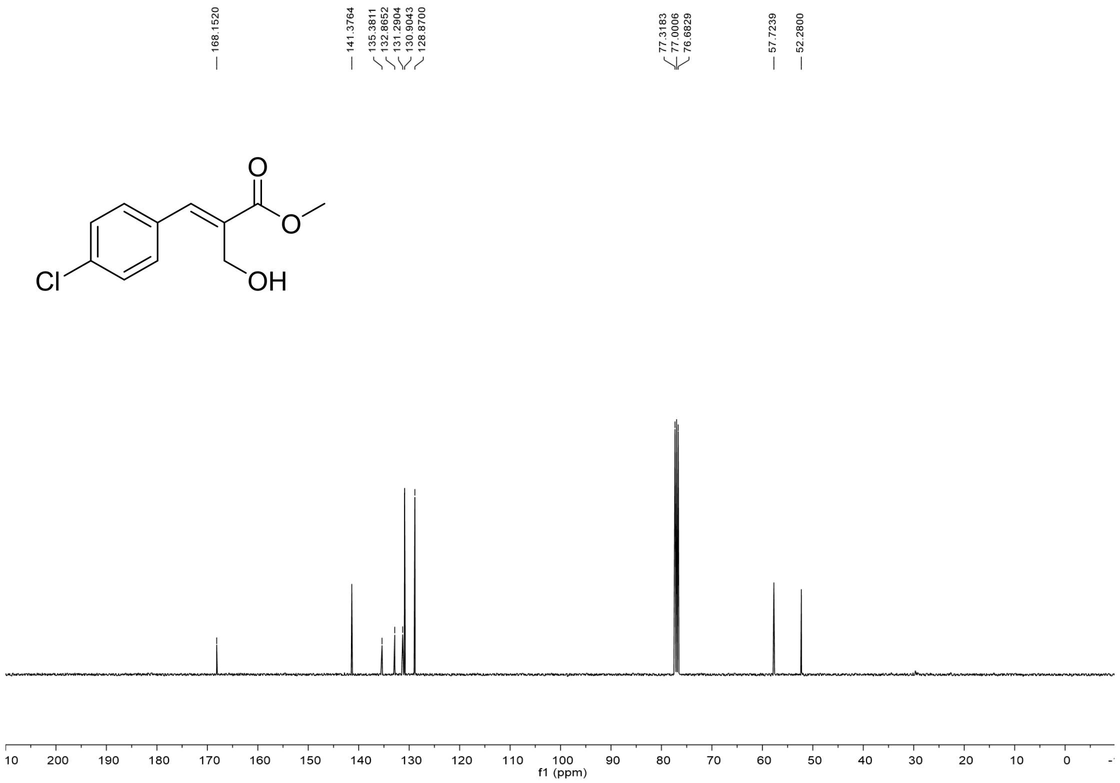

Figure S22 ^{1}H NMR and ^{13}C NMR spectra of **III-8b**

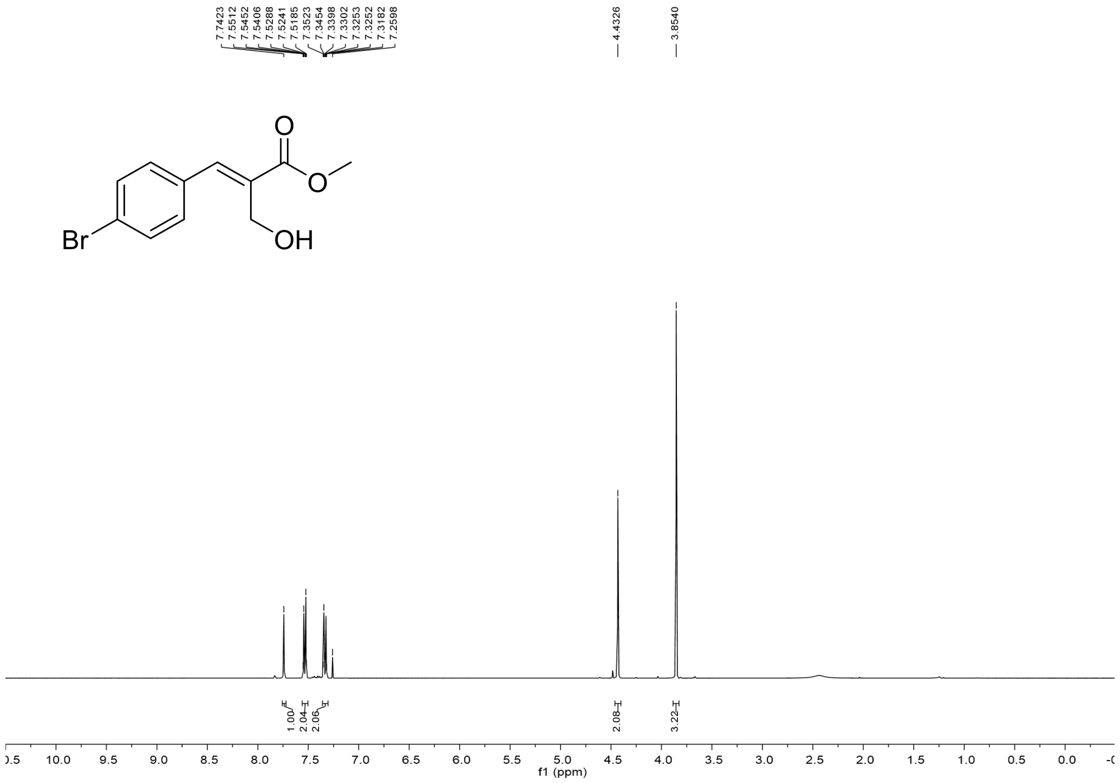

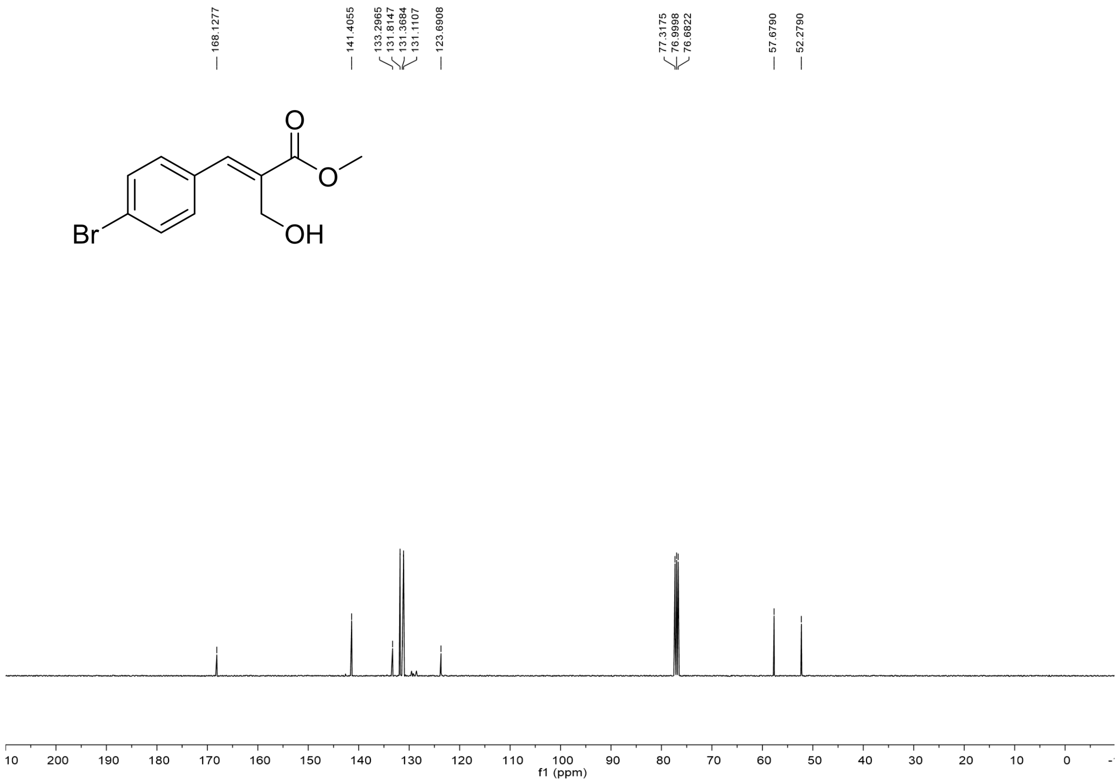

Figure S23 ^{1}H NMR and ^{13}C NMR spectra of **III-9b**

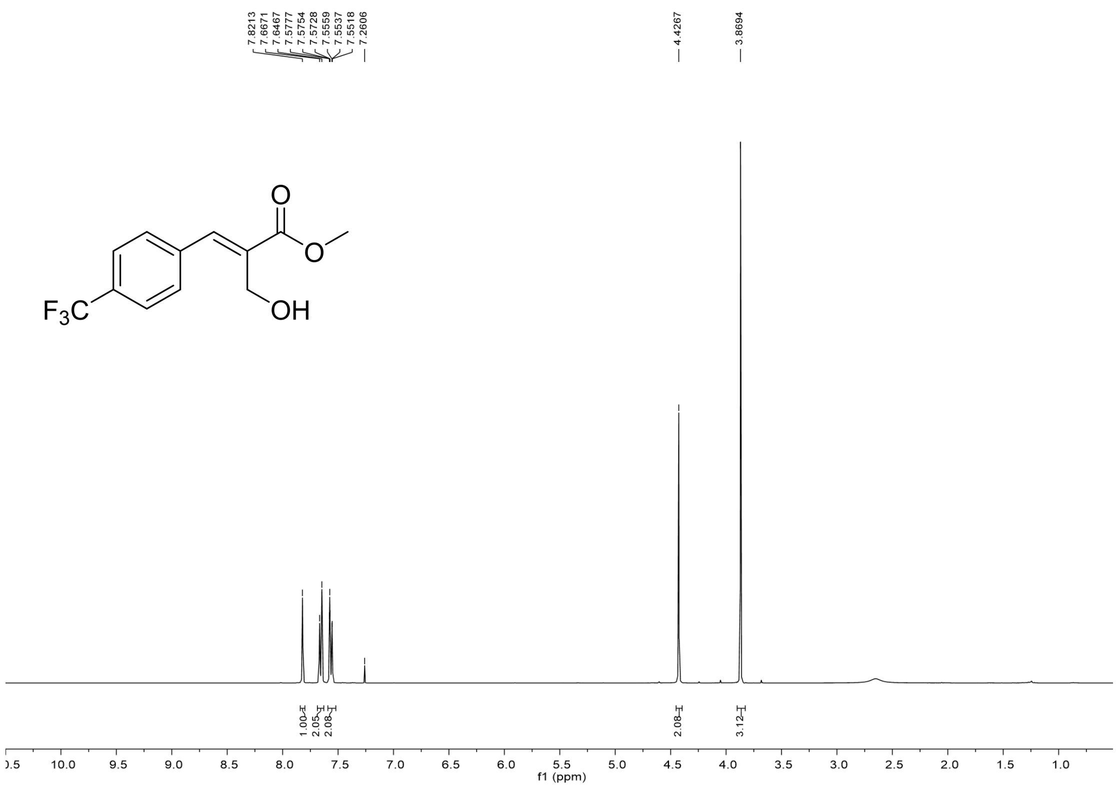

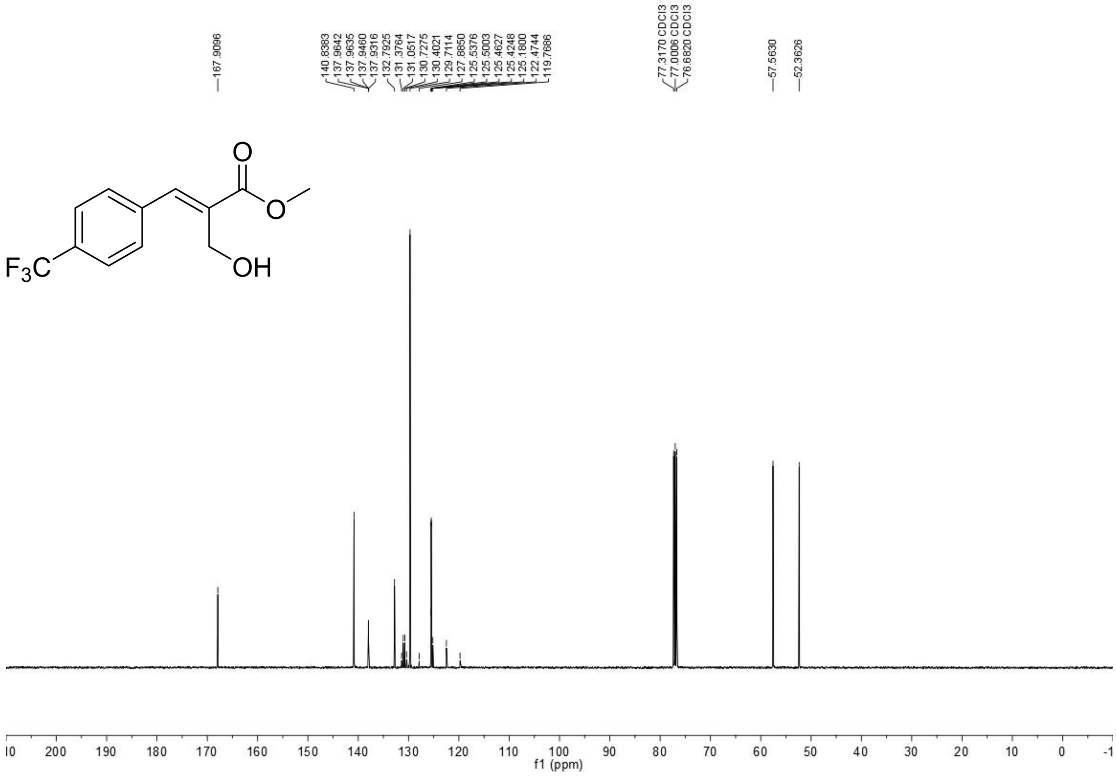

Figure S24 ^{1}H NMR and ^{13}C NMR spectra of **III-10b**

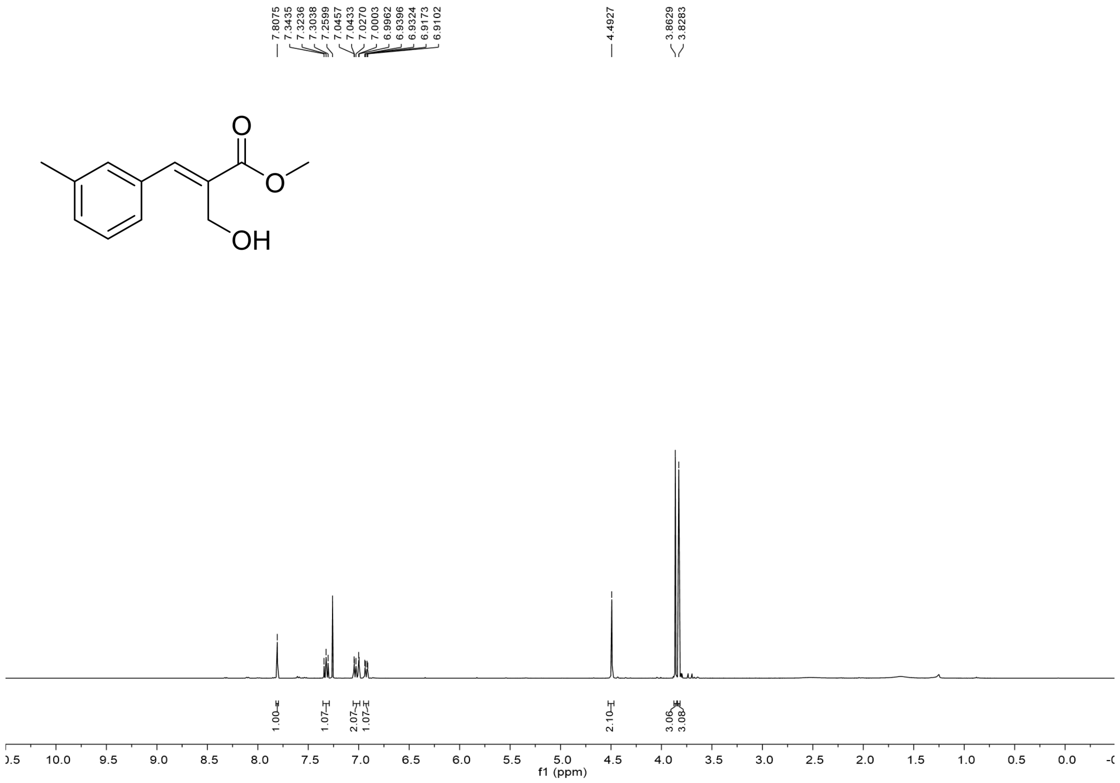

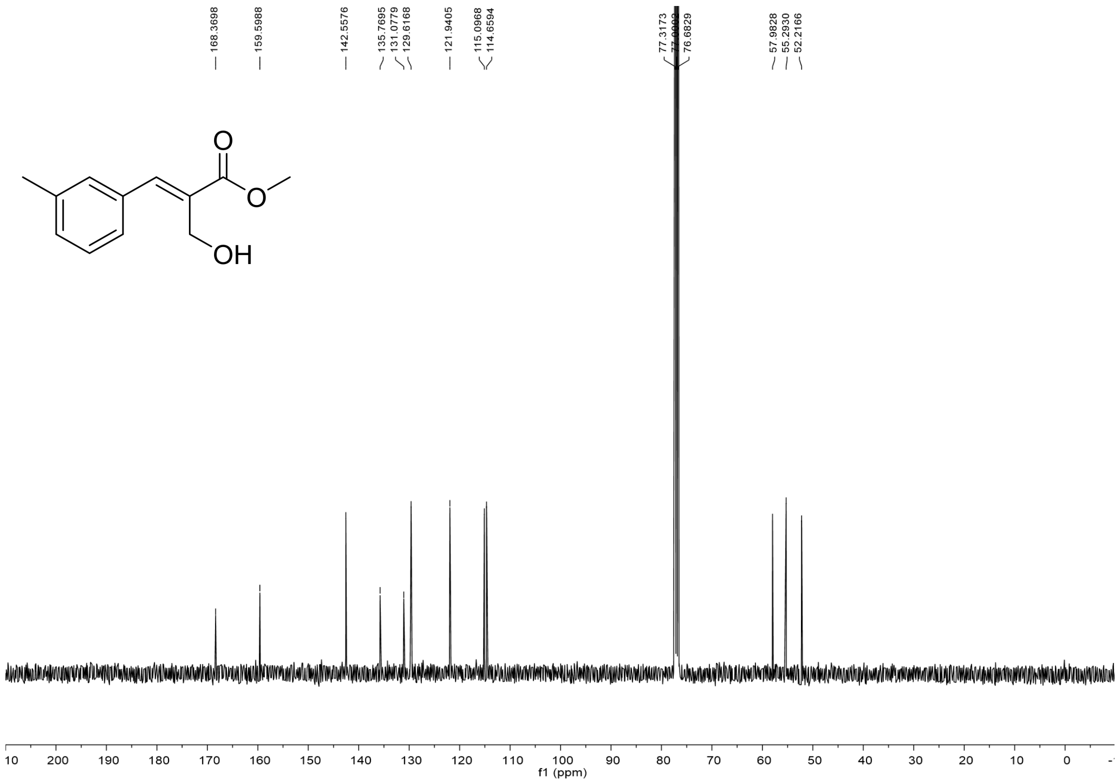

Figure S25 ^{1}H NMR and ^{13}C NMR spectra of **III-11b**

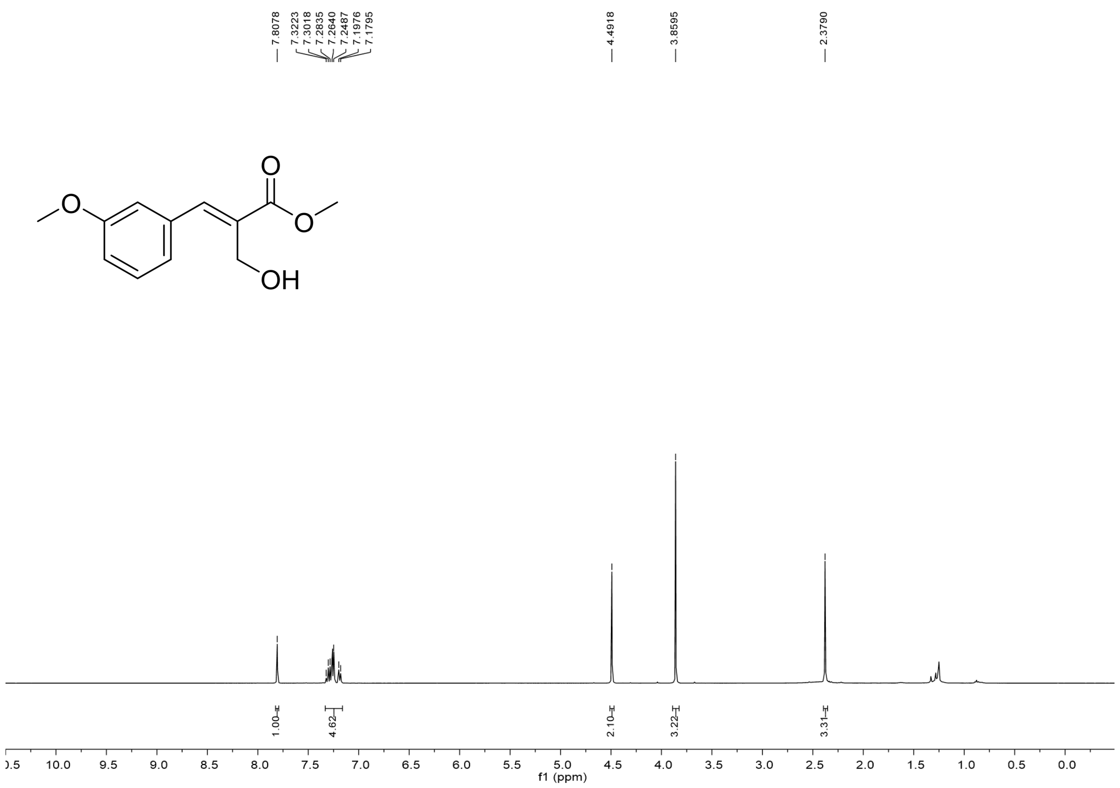

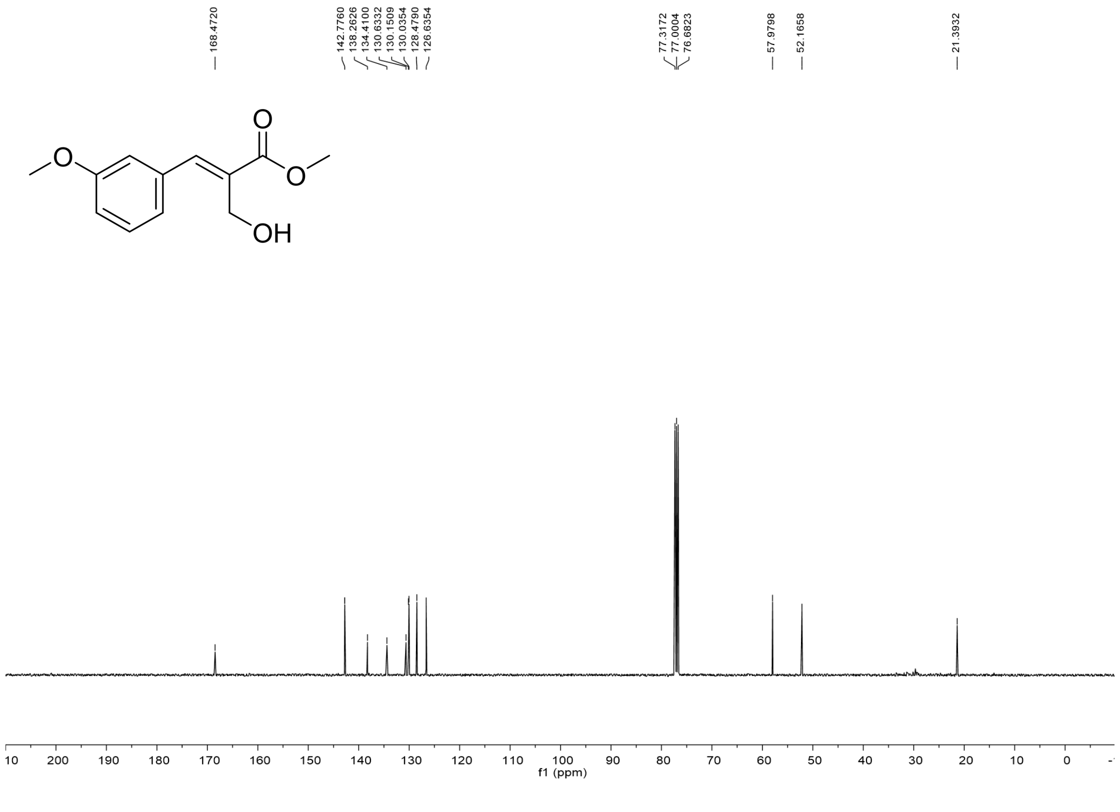

Figure S26 ^{1}H NMR and ^{13}C NMR spectra of **III-12b**

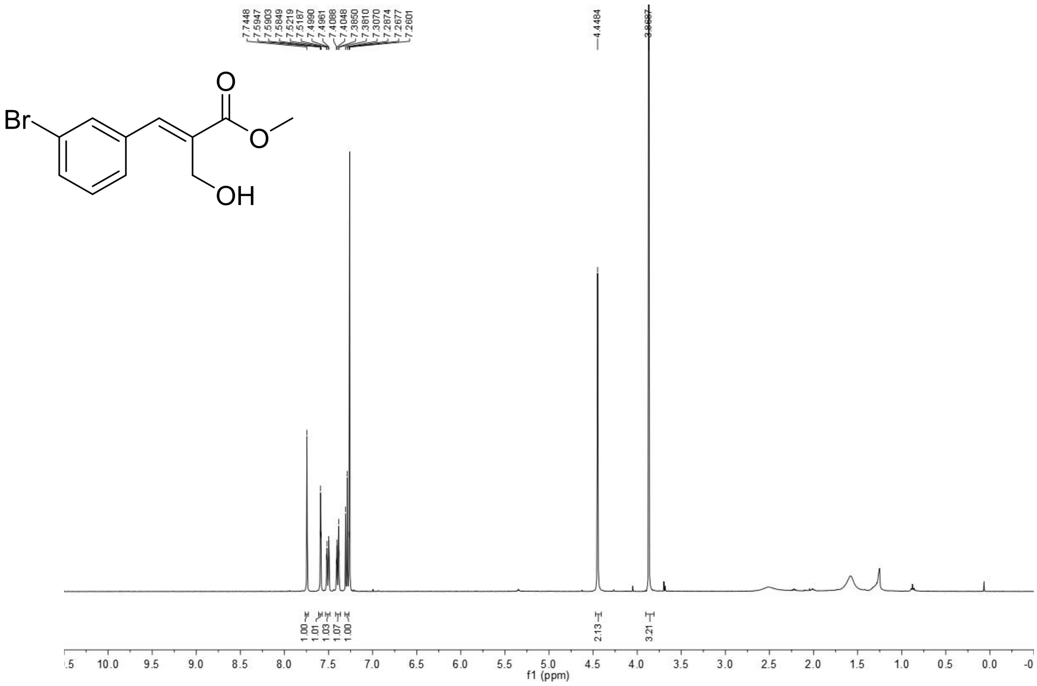

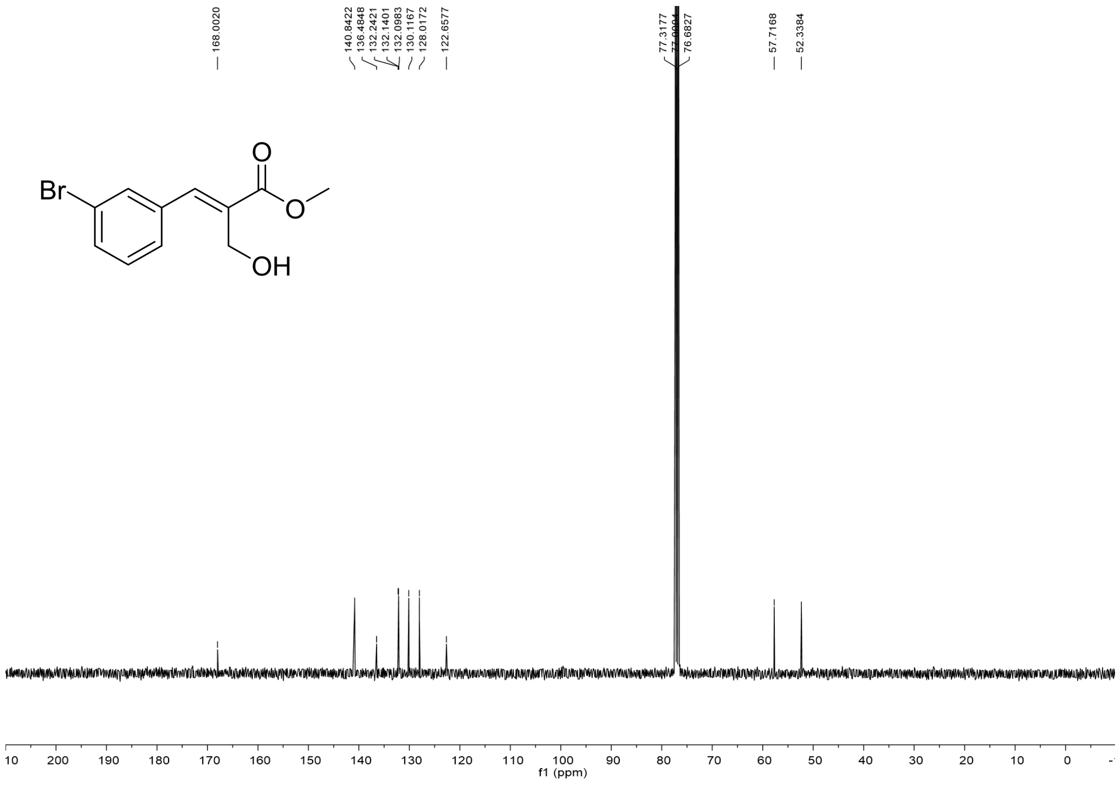

Figure S27 ^{1}H NMR and ^{13}C NMR spectra of **III-13b**

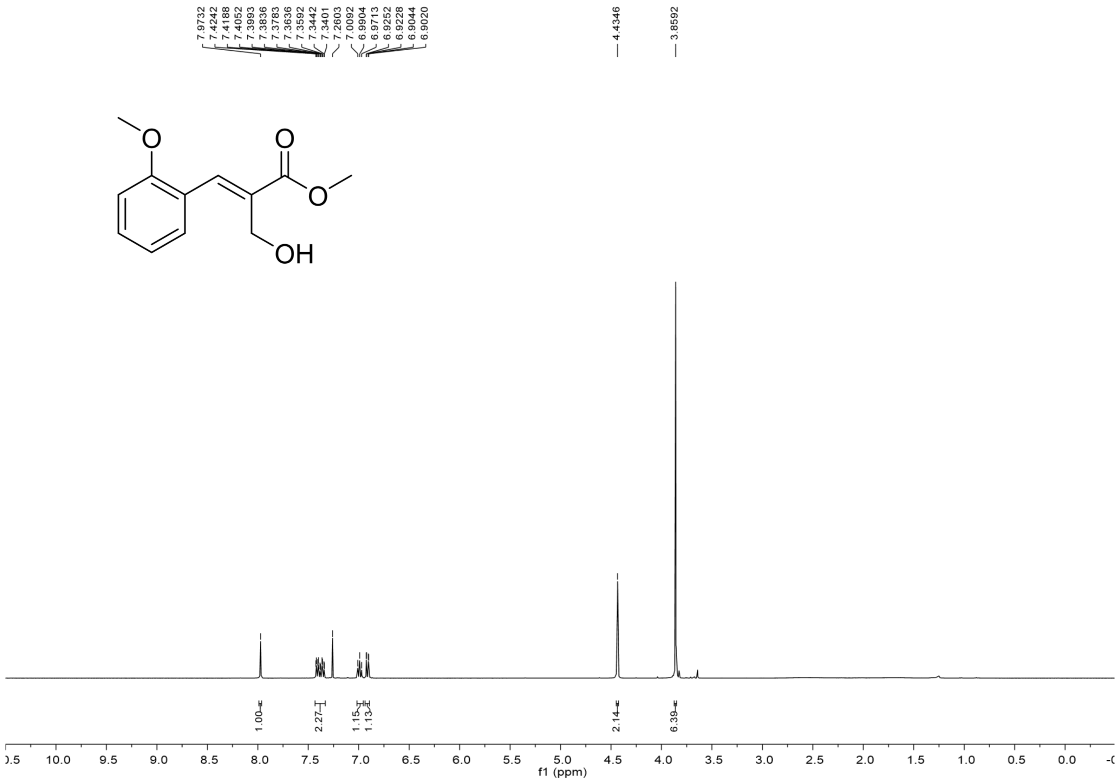

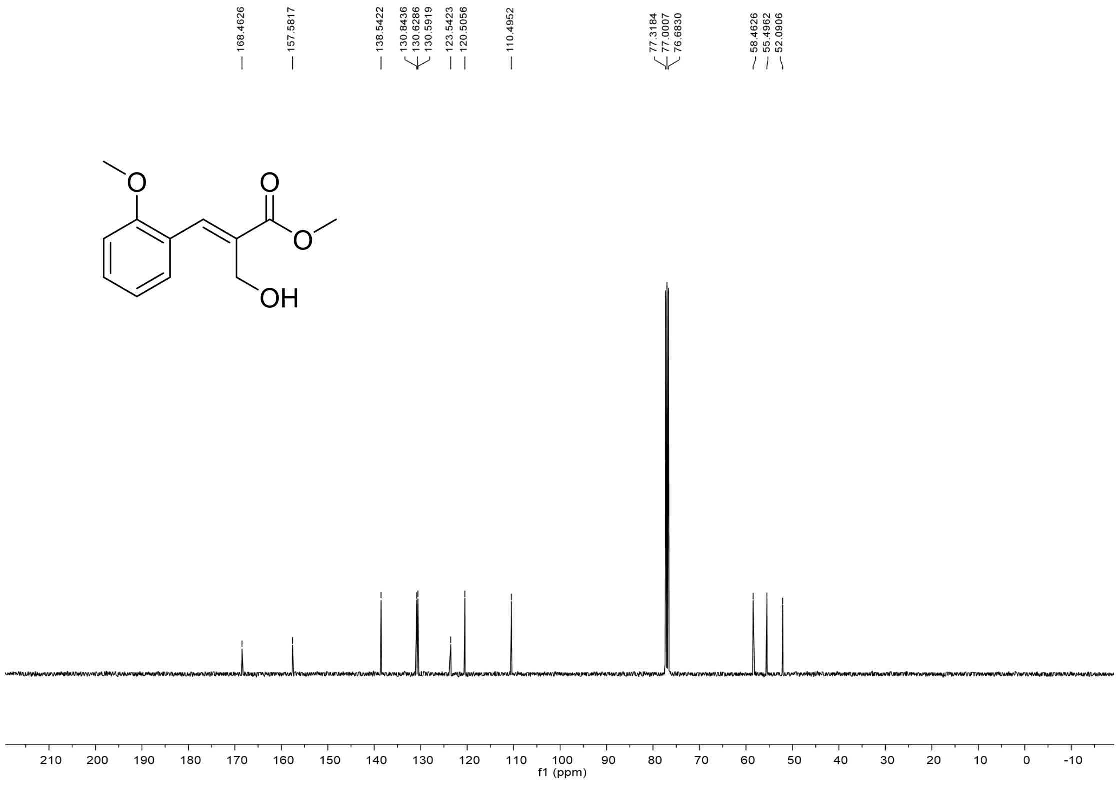

Figure S28 ^{1}H NMR and ^{13}C NMR spectra of **III-14b**

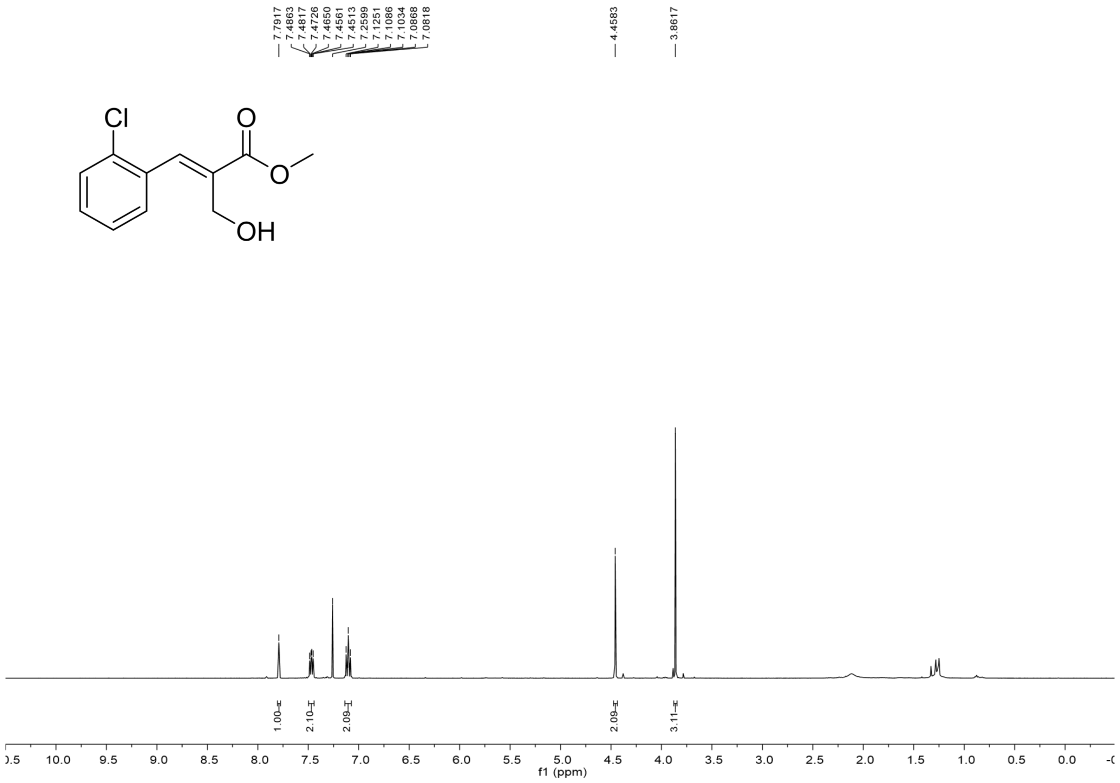

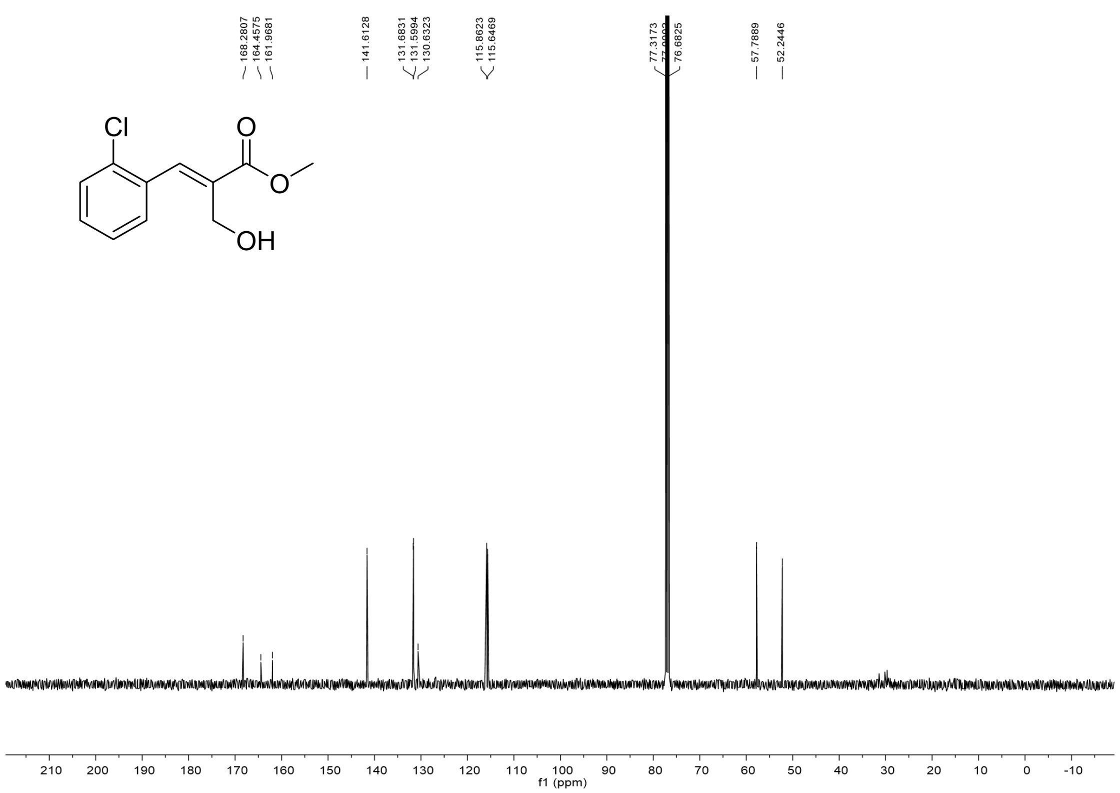

Figure S29 ^{1}H NMR and ^{13}C NMR spectra of **III-15b**

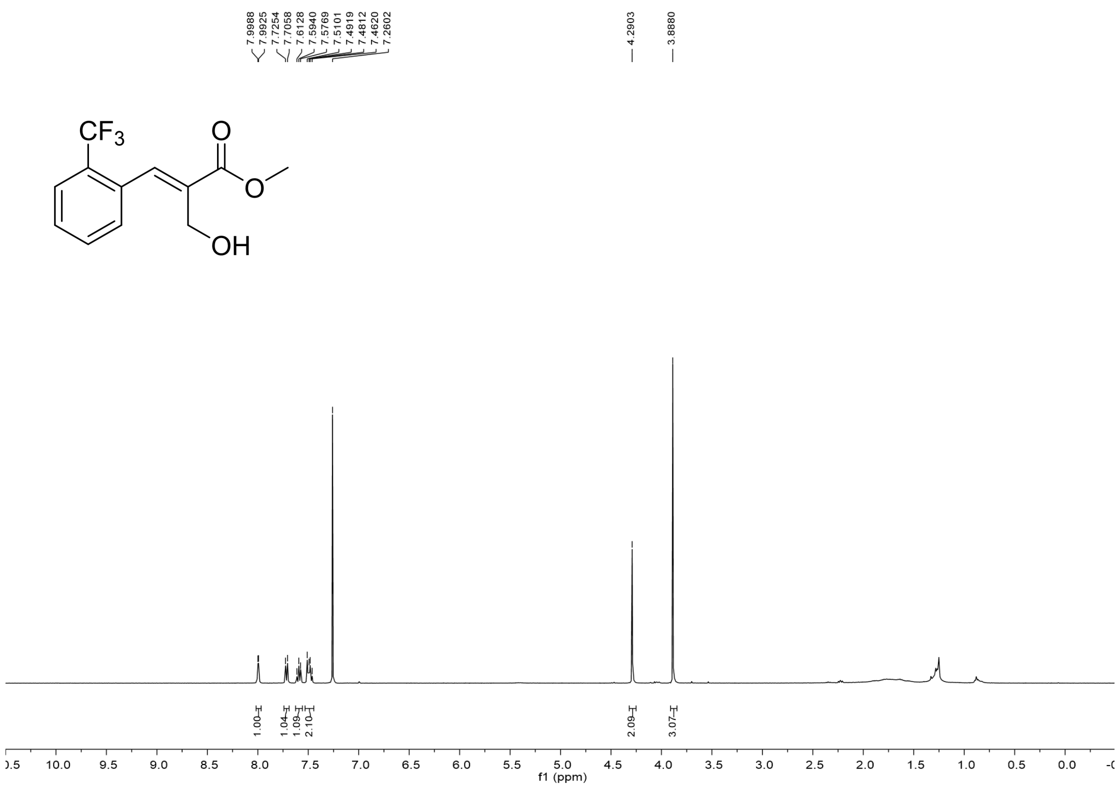

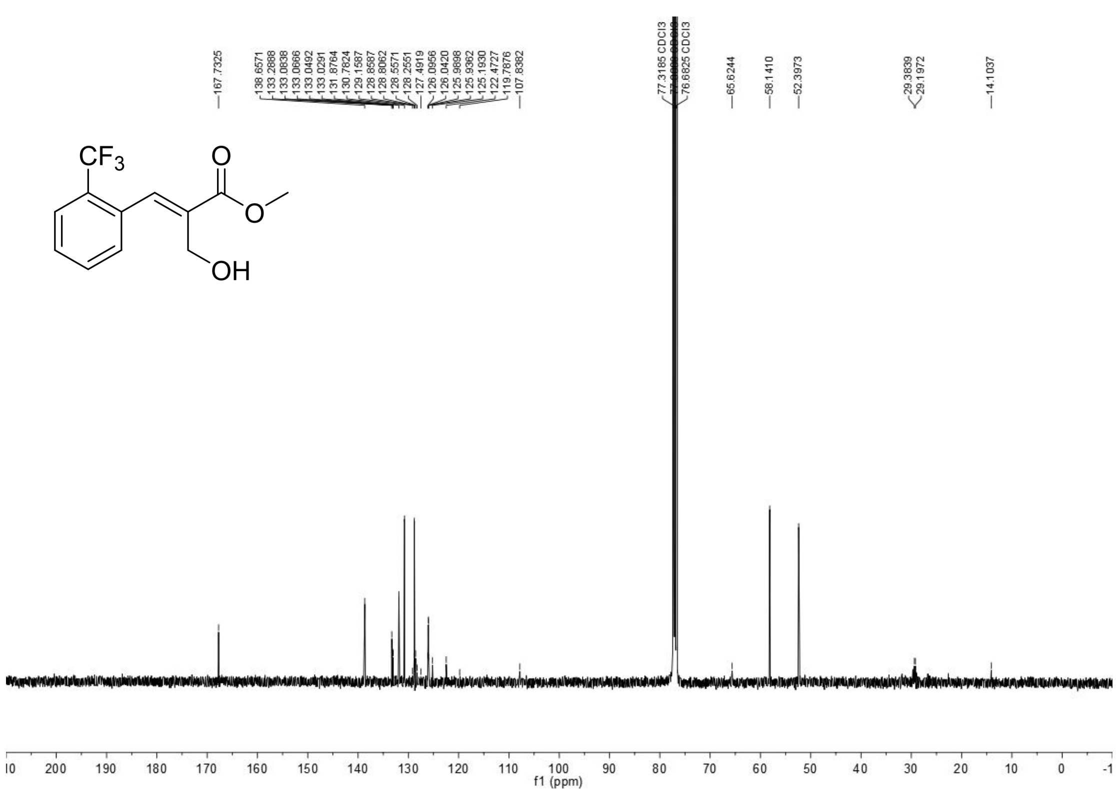

Figure S30 ^{1}H NMR and ^{13}C NMR spectra of **III-16b**

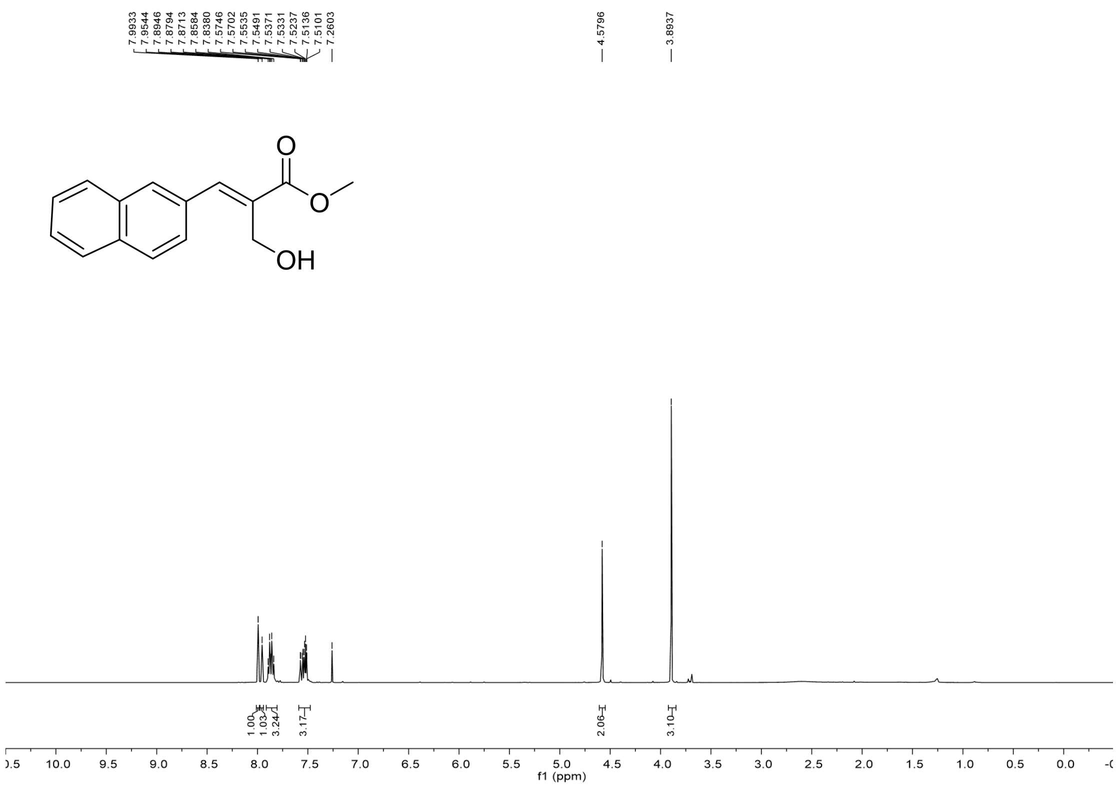

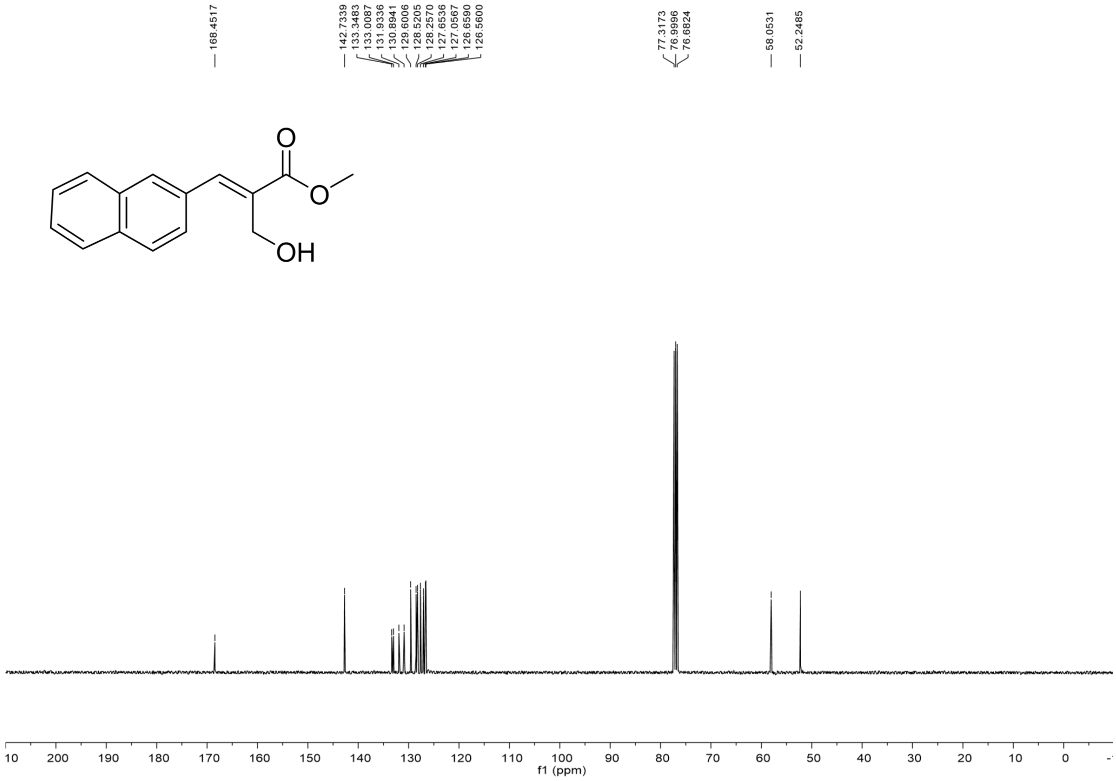

Figure S31 ^{1}H NMR and ^{13}C NMR spectra of **III-17b**

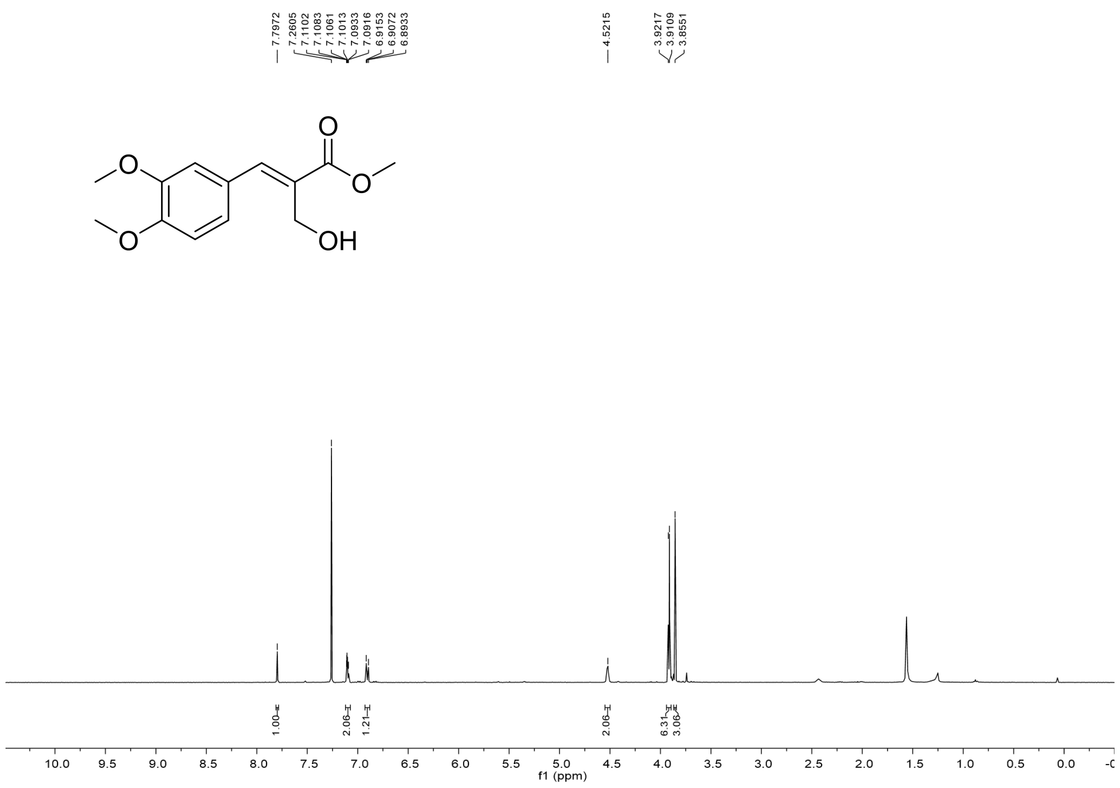

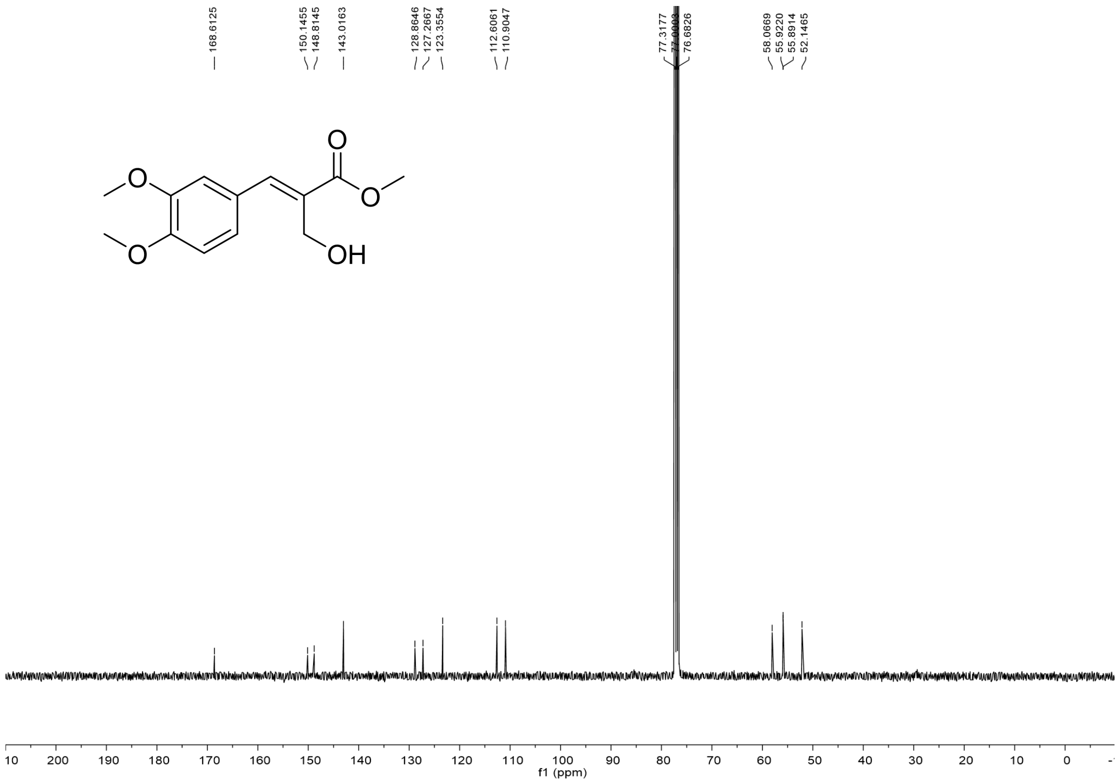

Figure S32 ^{1}H NMR and ^{13}C NMR spectra of **III-18b**

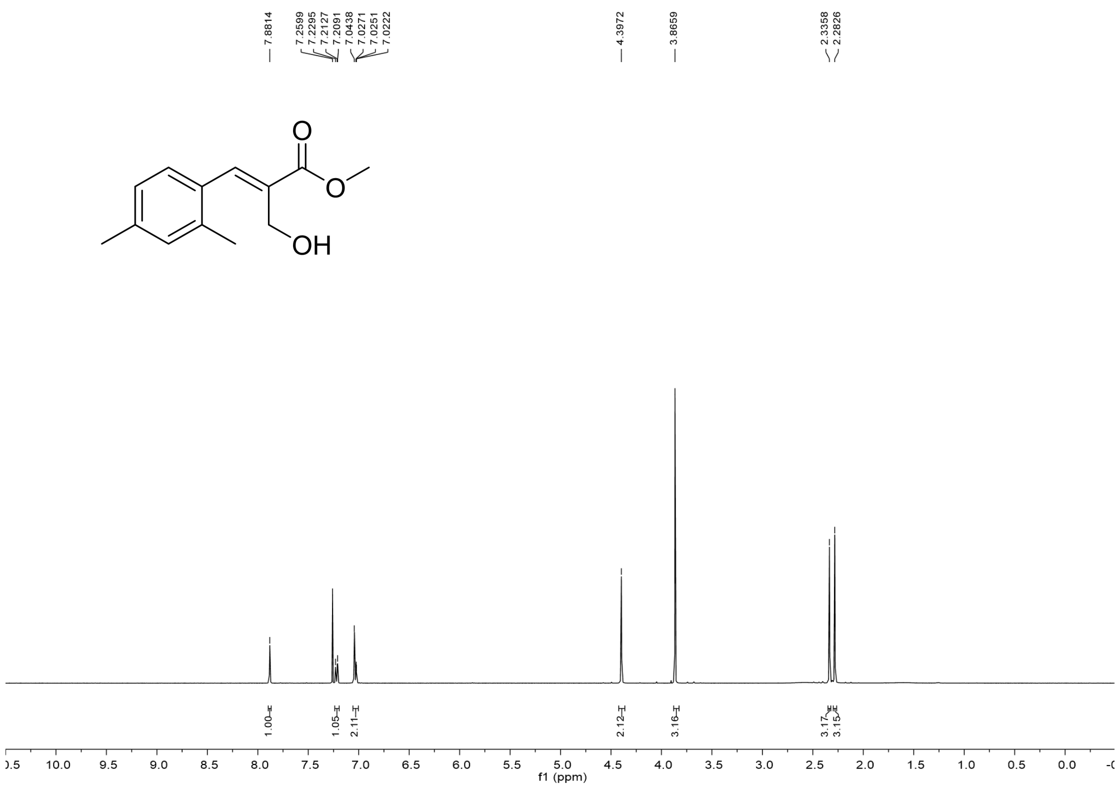

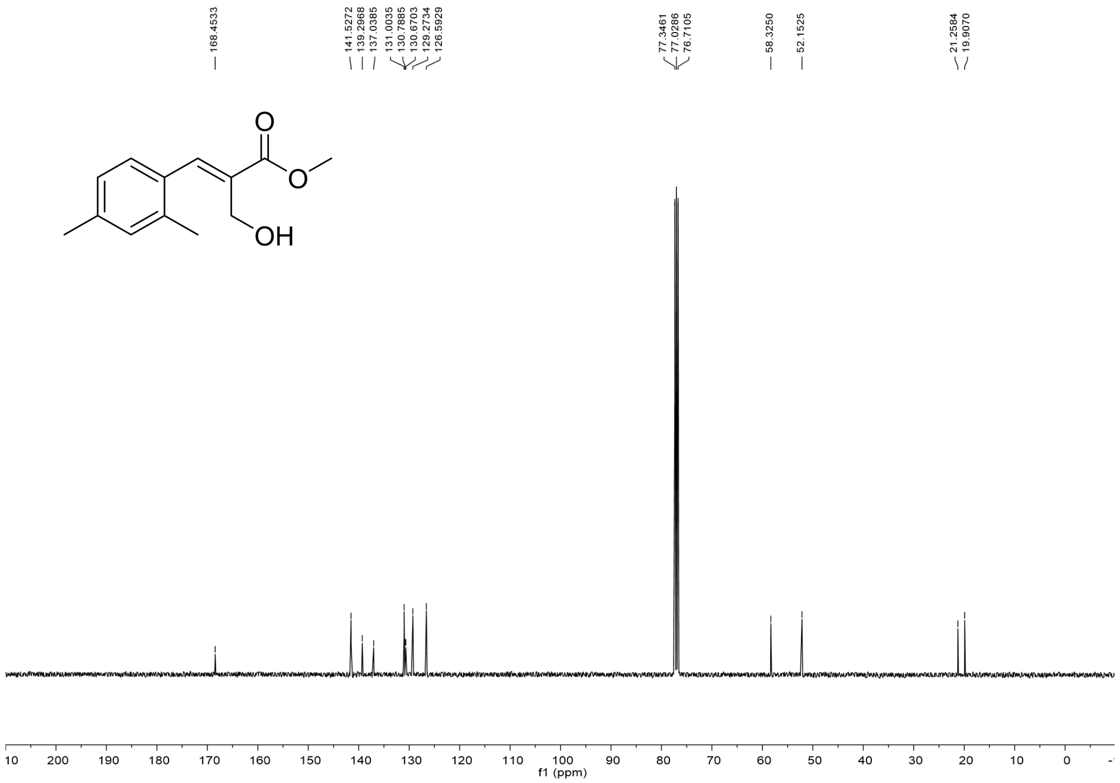

Figure S33 ^{1}H NMR and ^{13}C NMR spectra of **III-19b**

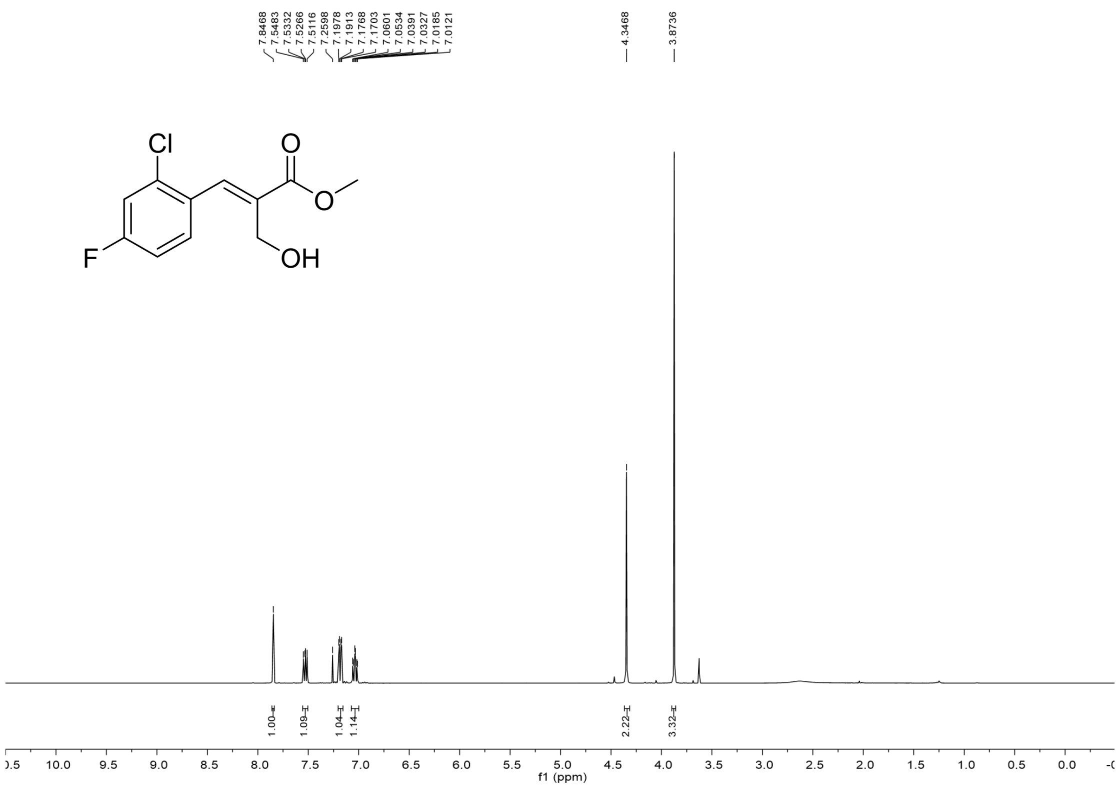

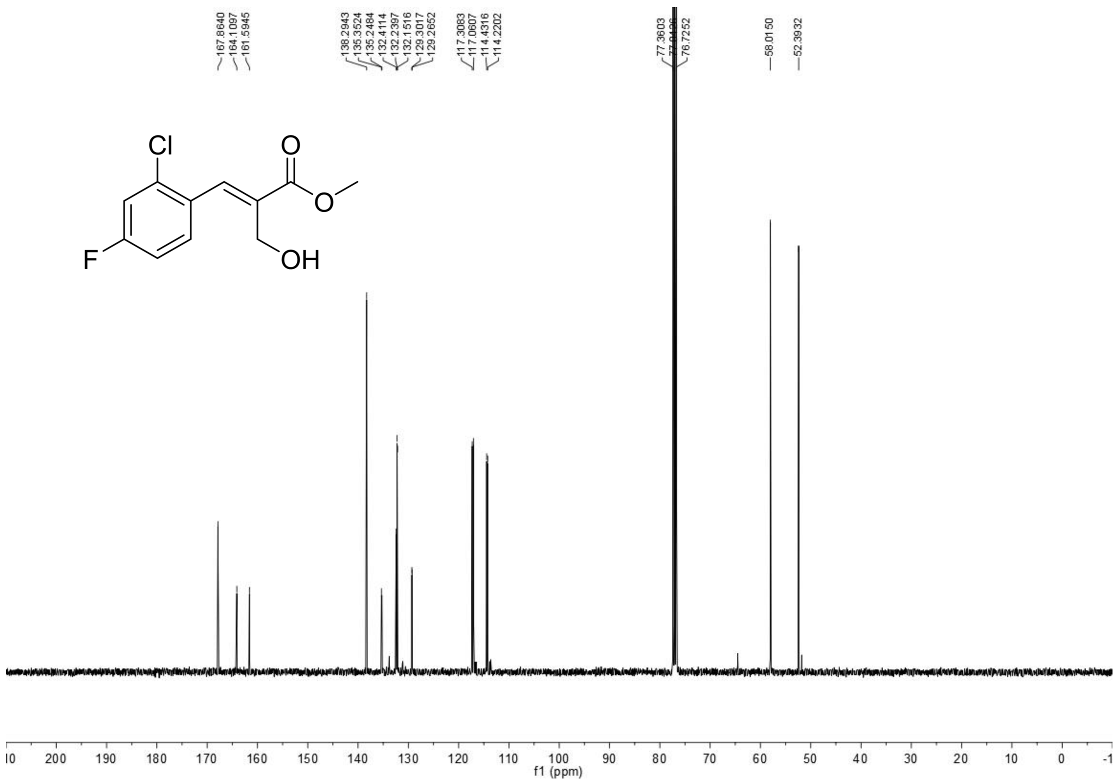

Figure S34 ^{1}H NMR and ^{13}C NMR spectra of **III-20b**

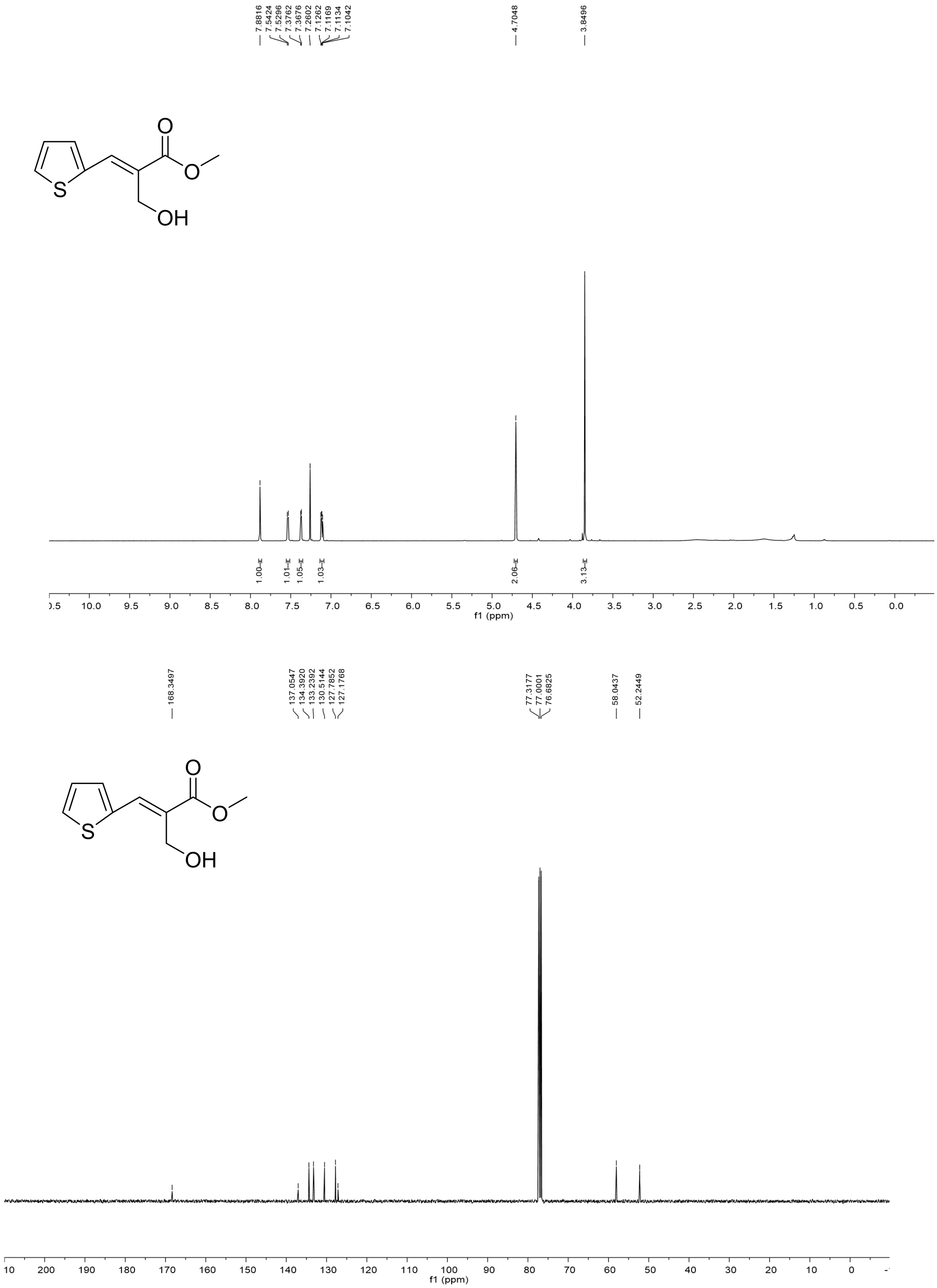

Figure S35 ¹H NMR and ¹³C NMR spectra of **III-21b**

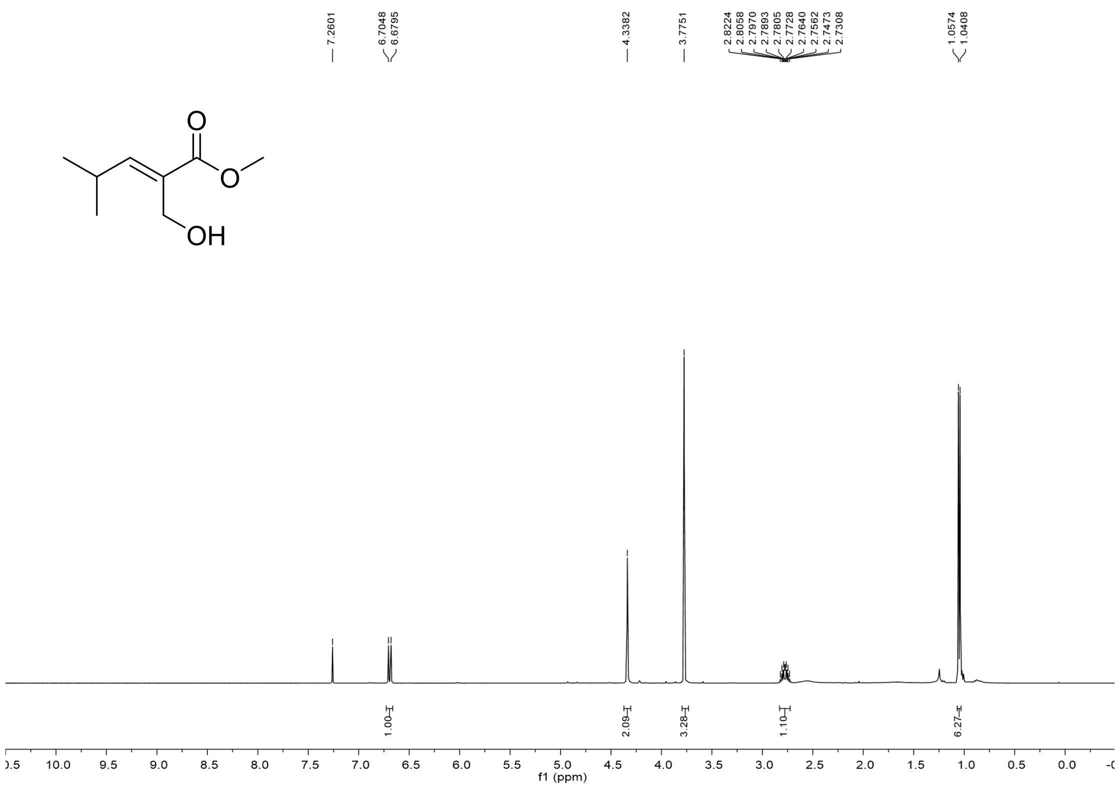

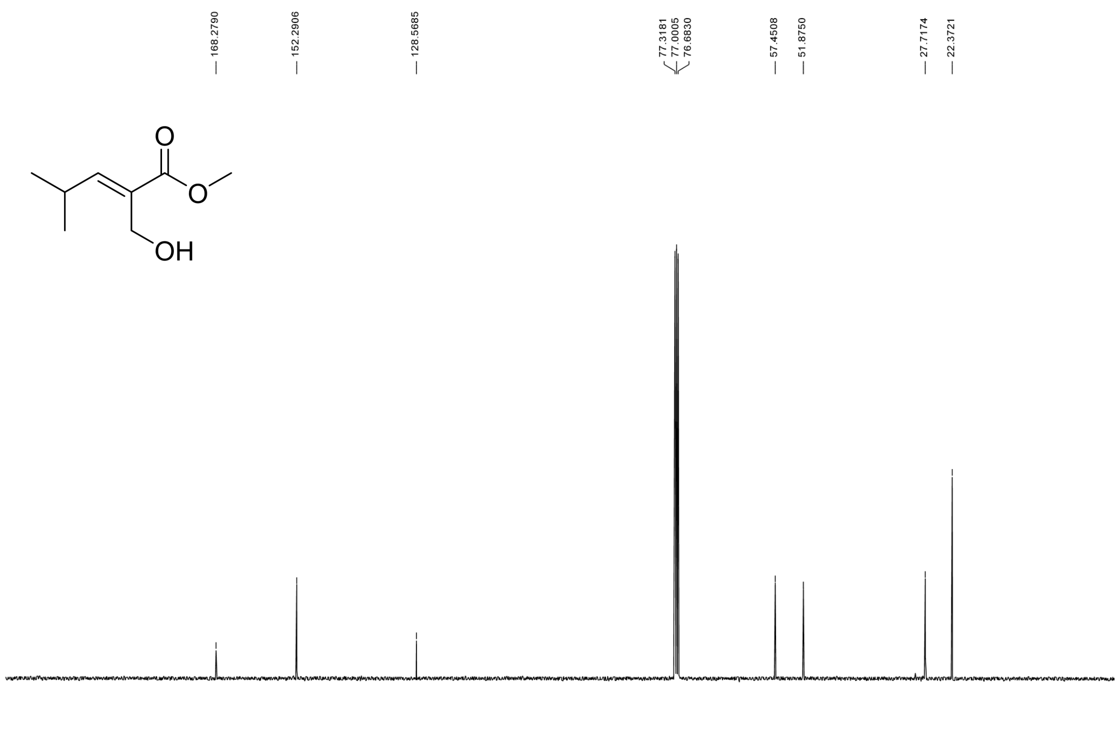

Figure S36 ^{1}H NMR and ^{13}C NMR spectra of **III-22b**

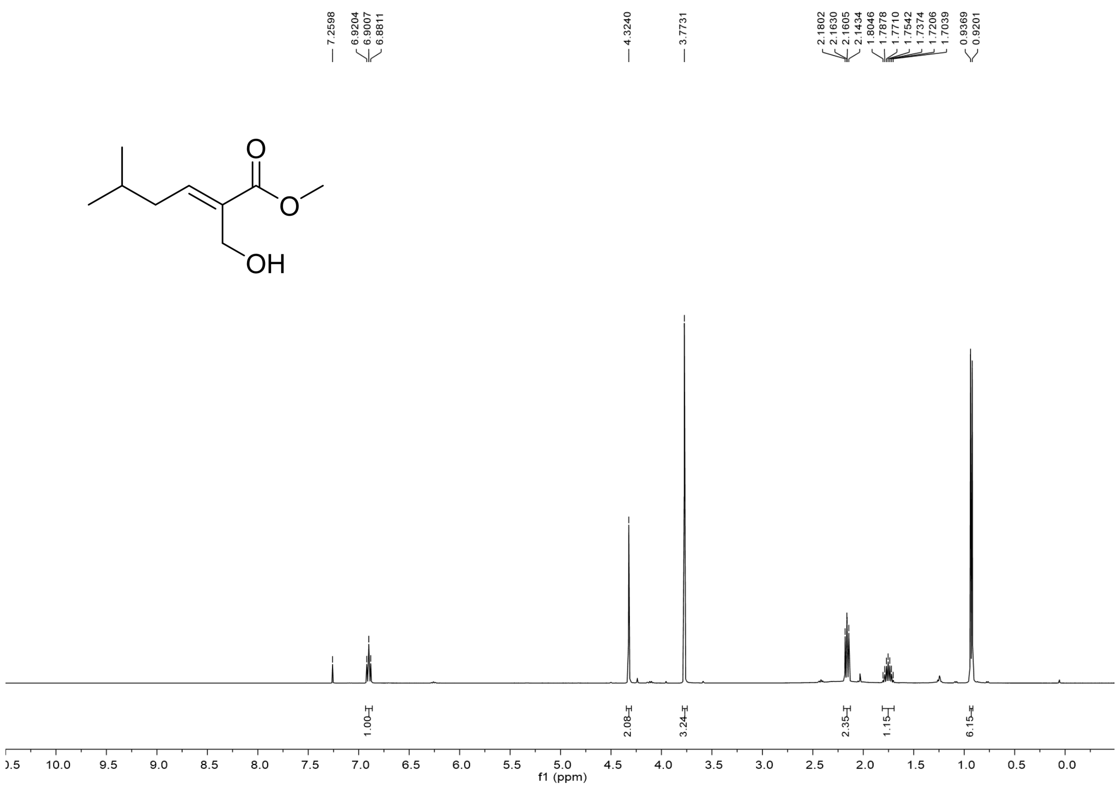

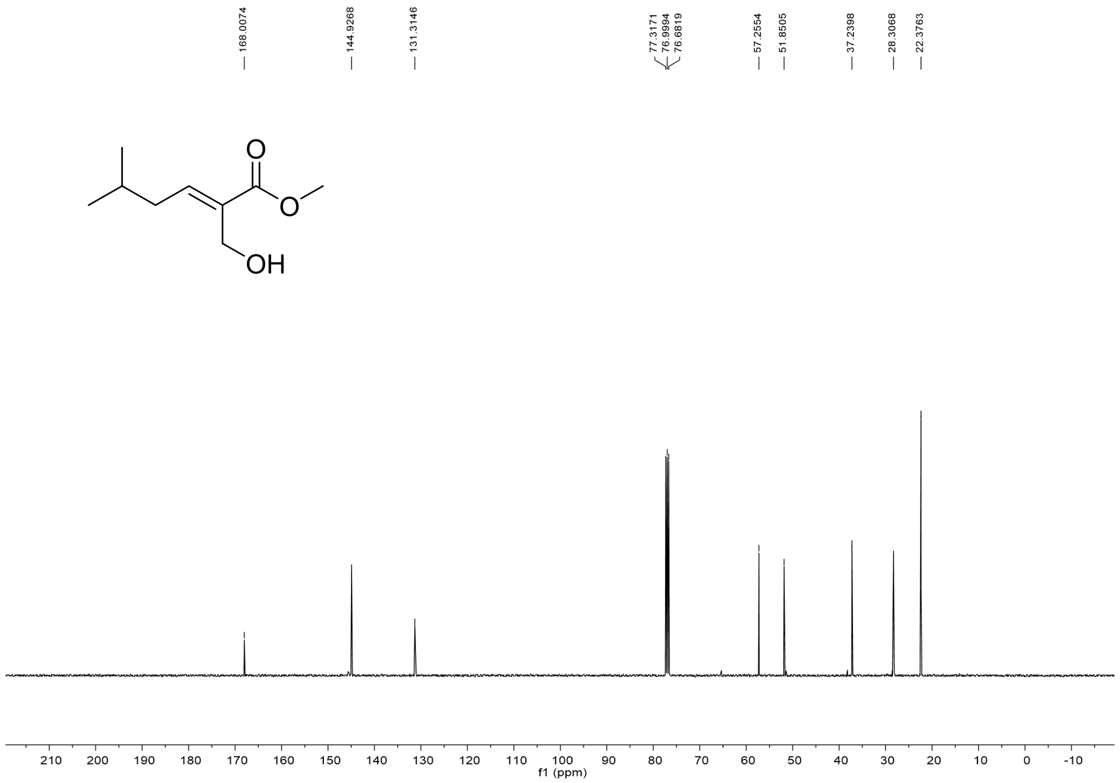

Figure S37 ^{1}H NMR and ^{13}C NMR spectra of **III-23b**

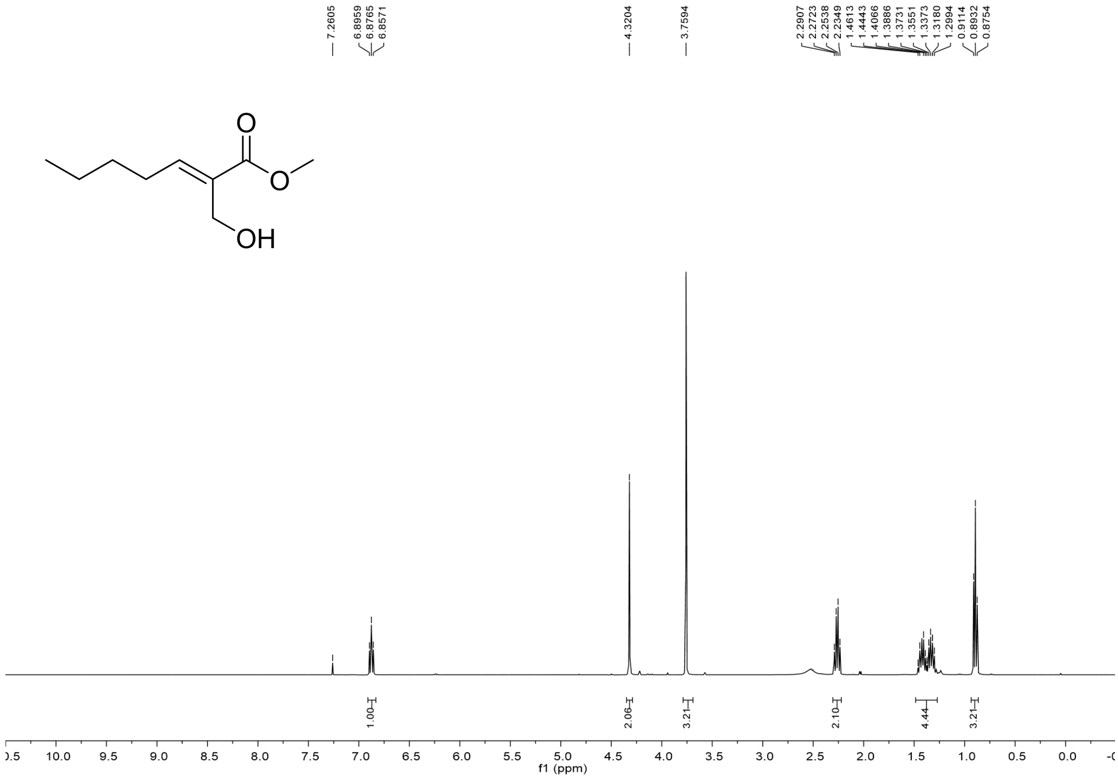

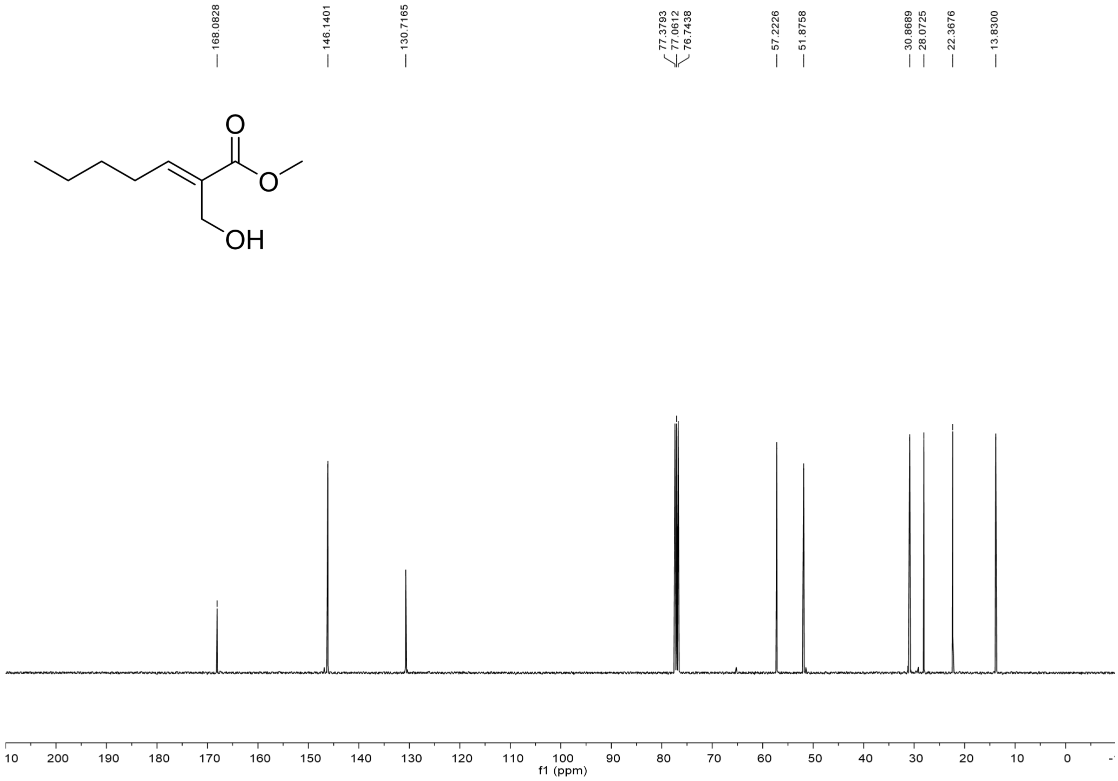

Figure S38 ^{1}H NMR and ^{13}C NMR spectra of **III-24b**

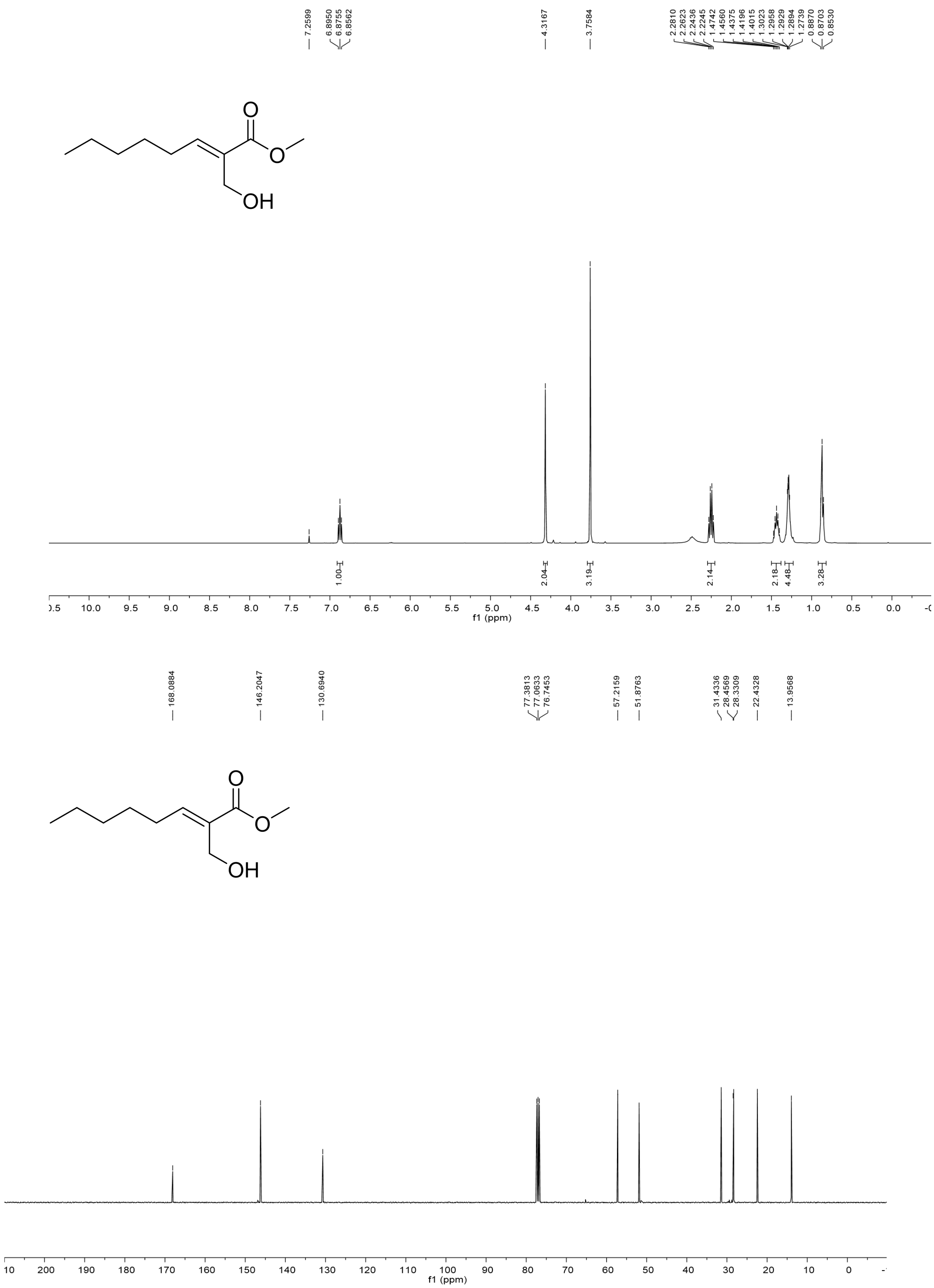

Figure S39 ^{1}H NMR and ^{13}C NMR spectra of **III-25b**

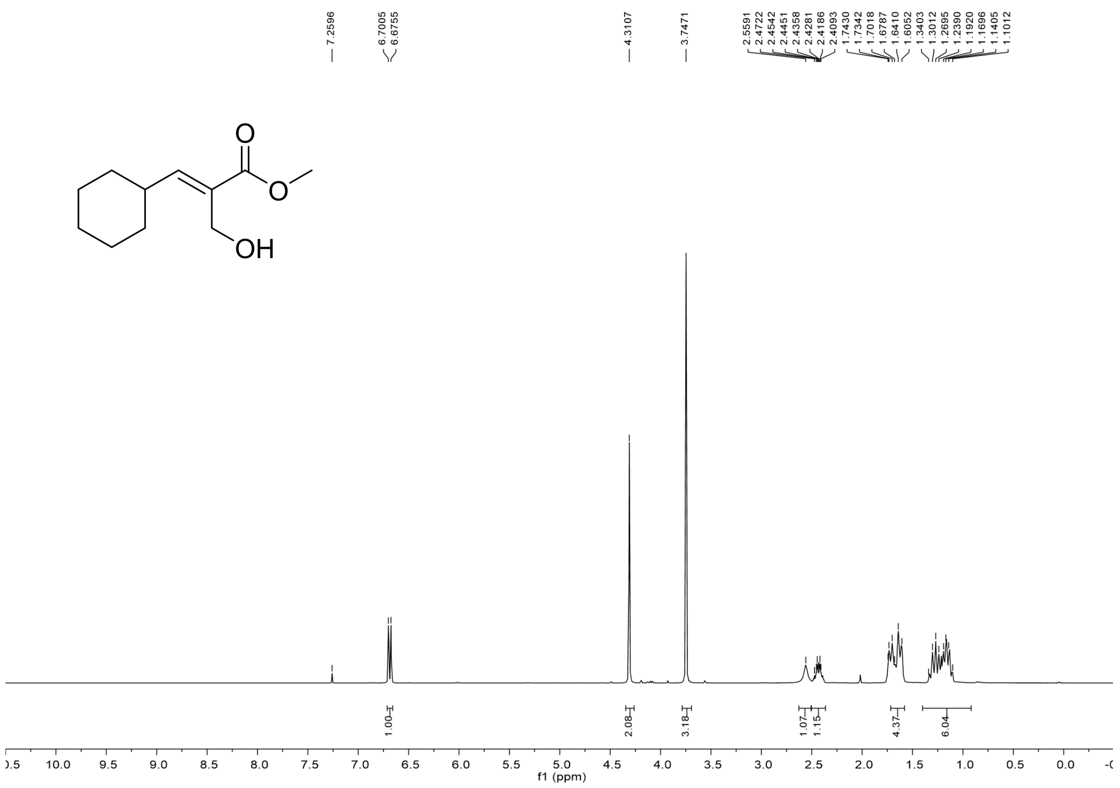

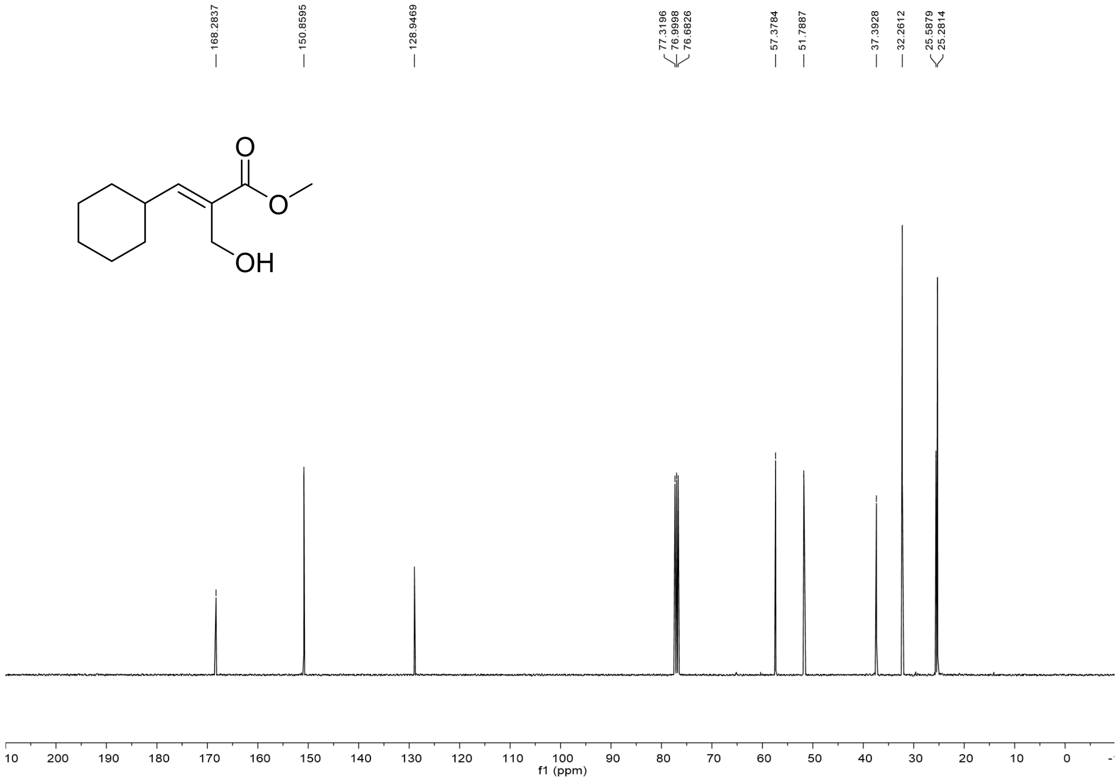

Figure S40 ^{1}H NMR and ^{13}C NMR spectra of **III-26b**

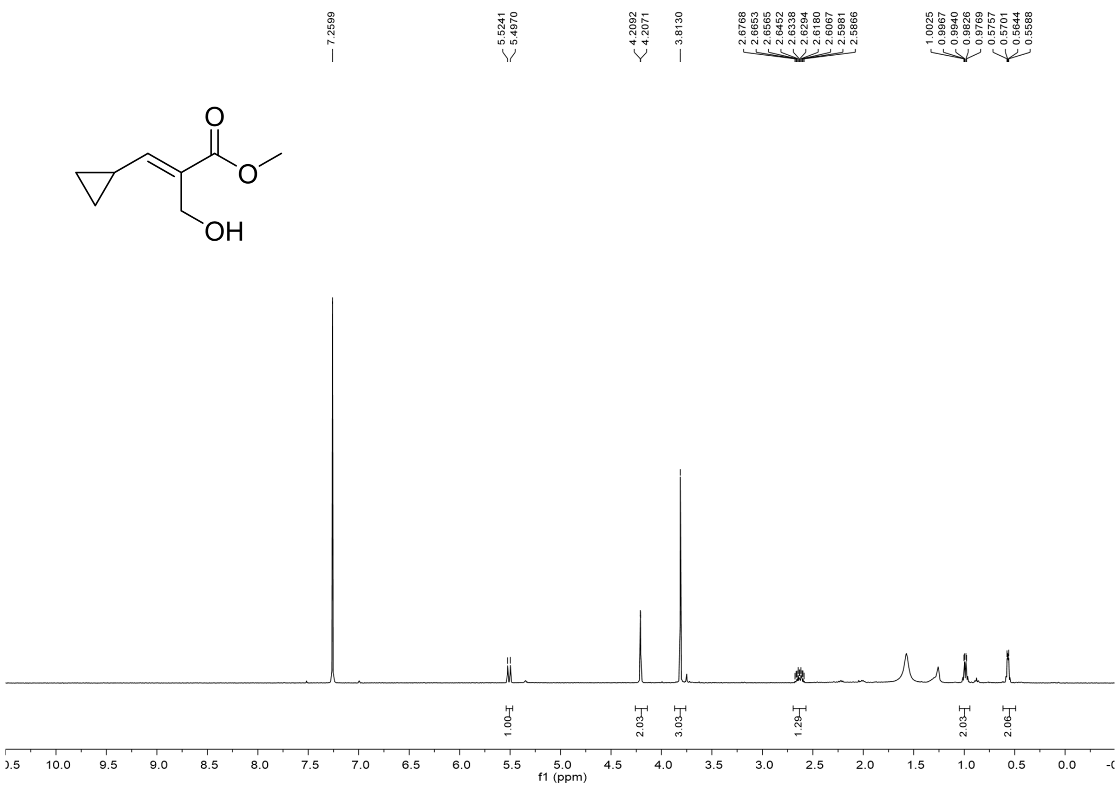

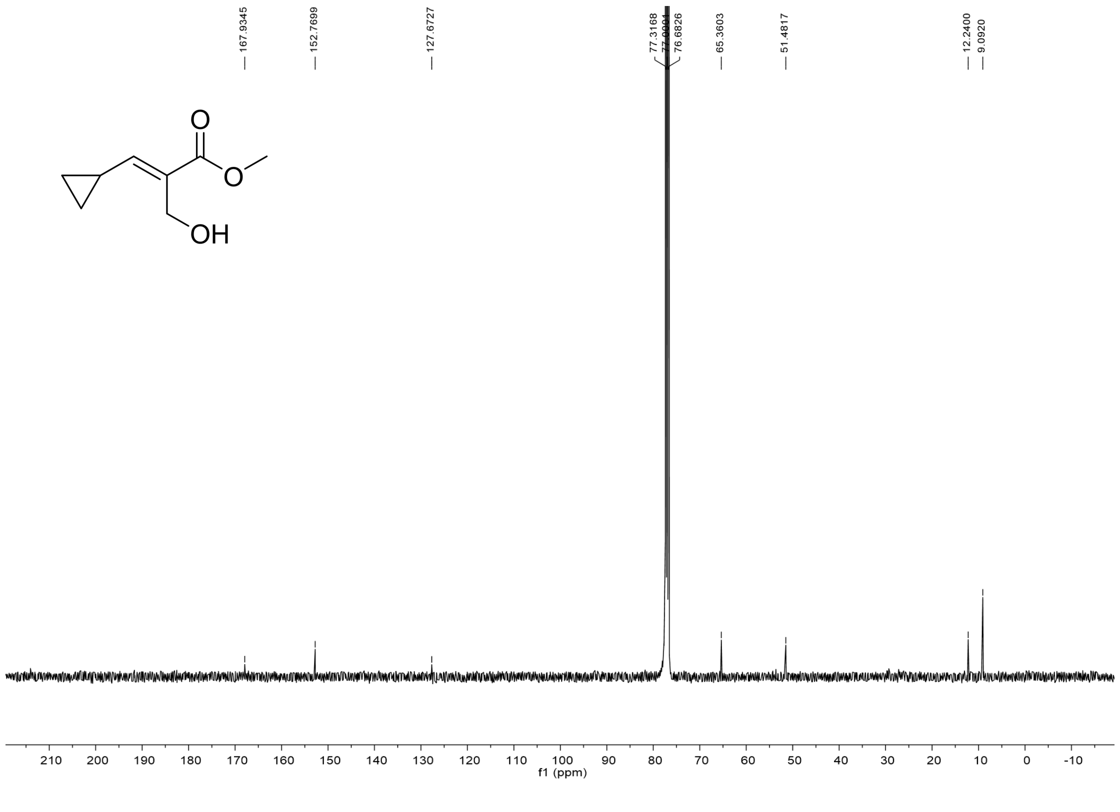

Figure S41 ^{1}H NMR and ^{13}C NMR spectra of **III-27b**

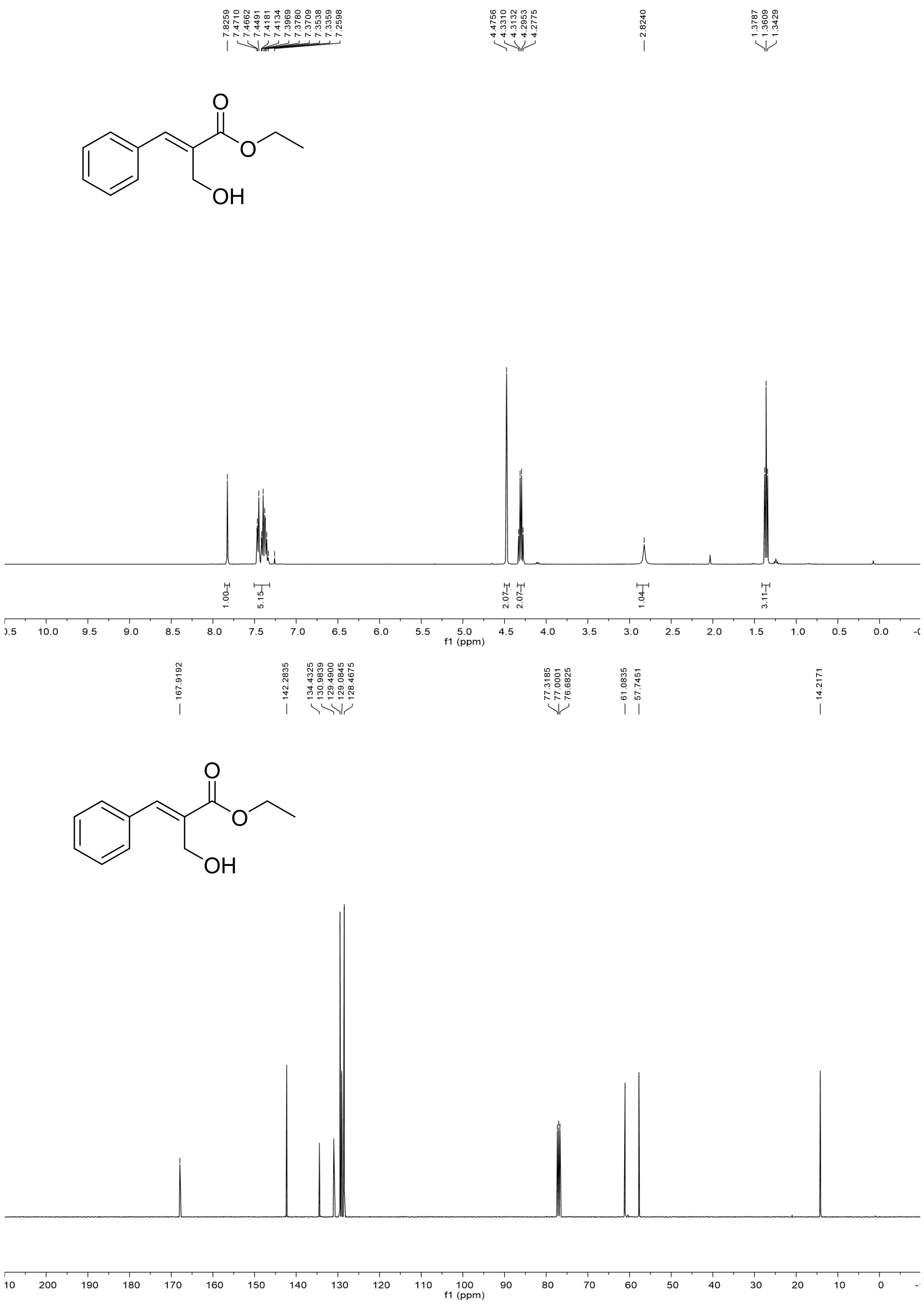

Figure S42 ^{1}H NMR and ^{13}C NMR spectra of **III-28b**
